# Temperature-dependent modulation by biaryl-based monomers of the chain length and morphology of biphenyl-based supramolecular polymers†

**DOI:** 10.1039/d1sc03974a

**Published:** 2021-09-06

**Authors:** Tomokazu Iseki, Mathijs F. J. Mabesoone, Mark A. J. Koenis, Brigitte A. G. Lamers, Elisabeth Weyandt, Lafayette N. J. de Windt, Wybren Jan Buma, Anja R. A. Palmans, E. W. Meijer

**Affiliations:** Institute for Complex Molecular Systems and Laboratory of Macromolecular and Organic Chemistry, Eindhoven University of Technology 5600 MB Eindhoven The Netherlands e.w.meijer@tue.nl; Material Science Research Laboratory, Kao Corporation Wakayama-shi Wakayama 640-8580 Japan; Institute of Microbiology, Eidgenössische Technische Hochschule Zürich Vladimir-Prelog-Weg 4 8093 Zurich Switzerland; Van't Hoff Institute for Molecular Sciences, University of Amsterdam Science Park 904 1098 XH Amsterdam The Netherlands; Institute for Molecules and Materials, FELIX Laboratory, Radboud University Toernooiveld 7c 6525 ED Nijmegen The Netherlands

## Abstract

Supramolecular copolymerizations offer attractive options to introduce structural and functional diversity in supramolecular polymer materials. Yet, general principles and structure–property relationships for rational comonomer design remain lacking. Here, we report on the supramolecular (co)aggregation of a phenylpyridine and bipyridine derivative of a recently reported biphenyl tetracarboxamide-based monomer. We show that both arylpyridines are poor monomers for supramolecular homopolymerizations. However, the two arylpyridines efficiently influence supramolecular polymers of a biphenyl-based polymer. The phenylpyridine derivatives primarily sequestrate biphenyl monomers, while the bipyridine intercalates into the polymers at high temperatures. Thereby, these two poorly homopolymerizing monomers allow for a fine control over the length of the biphenyl-based supramolecular polymers. As such, our results highlight the potential to control the structure and morphology of supramolecular polymers by tailoring the electronic properties of additives.

## Abstract 2

Supramolecular copolymerizations offer attractive options to introduce structural and functional diversity in supramolecular polymer materials.

## Graphical abstract

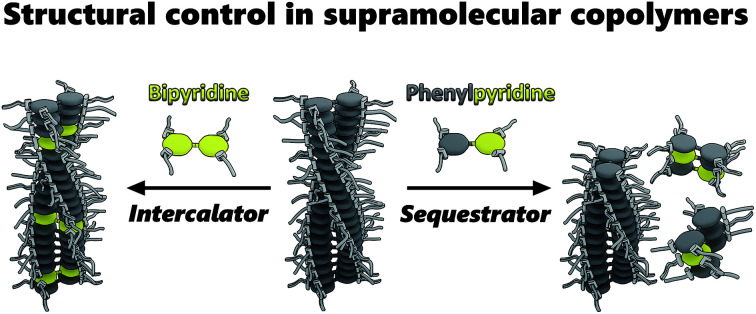

## Introduction

Additives play a central role in tailoring material properties in both synthetic and natural systems. In synthetic polymeric materials chain transfer agents^1–3^ or plasticizers^4–6^ alter the material at a microscopic and macroscopic level, respectively. A related role is played by additives that in some cases do not polymerize themselves but are great comonomers like in polystyrene-maleimide. In addition, copolymers, such as glycosaminoglycans,^7–9^ and repeating peptide sequences, as observed in *e.g.* silks,^10–12^ are important for many biomaterials.

Supramolecular polymers occupy a central role in this regard, as the dynamic nature of these materials holds great promise for integration into biological systems, while also providing attractive properties for optoelectronic applications and recycling of plastics. Seminal contributions are presented by Aida^13–15^ and Stupp^16–18^ for electronic and biological applications, respectively, while our group focused on mechanical properties.^19^ However, despite a few decades of research, the development of design principles to control the structure of supramolecular materials with small amounts of comonomers and/or additives is still in its infancy.^20–23^

From all the supramolecular polymers introduced so far, the cooperative polymerization of hydrogen-bonded disk-like molecules into helical one-dimensional aggregates, has been studied in most detail. The focus in supramolecular copolymerizations in this class was initially limited to fundamental stereochemical studies in so-called sergeant-and-soldiers^24–26^ and majority-rules^27–30^ experiments. Recent years have shown a strong interest in diversifying polymeric structure and the introduction of function through copolymerizations. Supramolecular block copolymers,^31–34^ as well as alternating^35^ and random copolymers^36^ have been reported for a wide range of discotic monomers. Complementing the experimental work with theoretical and computational methods, such as mass-balance^37^ and statistical mechanical models^35^ or molecular dynamics simulations,^38,39^ has shown to be crucial for a full interpretation of the results. In most of the systems reported, the employed comonomers are of comparable chemical nature, which facilitates incorporation of one monomer into aggregates of the other. Porphyrins form a notable exception to this observation, since complexation of small ligands to metallated porphyrins only occurs on free monomers or on chain ends of the supramolecular polymer.^40–44^ As a result, only a handful of chain cappers or sequestrators have been successfully designed^45–50^ and the current lack of general principles to rationally designed comonomers in supramolecular polymers impedes further application of these functionally diverse materials.

**Fig. 1:**
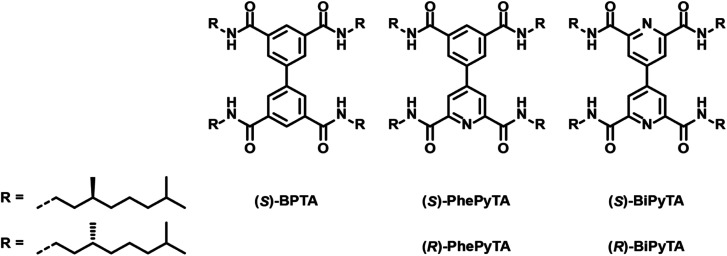
Chemical structures of the **(S)-BPTA**, **(S)** and **(R)-PhePyTA** and **(S)-BiPyTA** compounds used in this study.

In our search for fundamental principles that determine the role of comonomers and additives in supramolecular polymerizations, we were inspired by the potential for electronically diverse monomer scaffolds offered by the biaryl tetracarboxamide platform we reported recently.^51^ This biphenyl-tetracarboxamide-based monomer (**(S)-BPTA**, Fig. 1) forms supramolecular polymers in apolar solvents such as methylcyclohexane (MCH). Upon binding minute amounts of codissolved monomeric water,^52^**(S)-BPTA**-based supramolecular polymers show remarkable structural transformations. Moreover, we showed that small steric variations in the aliphatic side chain of **(S)-BPTA** can lead to various unforeseen, ‘abnormal’ sergeants-and-soldiers effects, highlighting the delicate energy balances that operate in supramolecular copolymerizations.^53^ We envision that further control over and diversity in the supramolecular architecture of **BPTA**-based polymers can be obtained, not by tuning peripheral steric interactions, but rather by controlling electronic interactions at the core of the polymer.^23^ To this end, we designed two new biaryl analogues, *i.e.* the tetracarboxamides of 4-phenylpyridine and 4,4′-bipyridine (**PhePyTA** and **BiPyTA**, respectively, see Fig. 1).

Here, we first disclose the scope and limitations of the homopolymerization of both **PhePyTA** and **BiPyTA**. We show that **PhePyTA** can assemble into two different types of supramolecular polymers at high concentrations, one of which is dependent on the presence of water. The less-soluble **BiPyTA** assembles into a single type of supramolecular polymer already at low concentration. Next, we investigate the effect of these electron-deficient analogues in the copolymerization of **(S)-BPTA** with **(S)-PhePyTA** and **(S)-BiPyTA**. We find that both **PhePyTA** and **BiPyTA** can act as temperature-dependent chain-length modulators of the **BPTA**-based supramolecular copolymers. As such, tailoring electronic properties of additives may pave the way towards supramolecular polymer architectures as diverse as seen in covalent polymer science.

## Results and discussion

Complete synthetic procedures and the full molecular characterization of the new monomers are given in the ESI.†

### Water modulates the length of PhePyTA-based aggregates

**Fig. 2:**
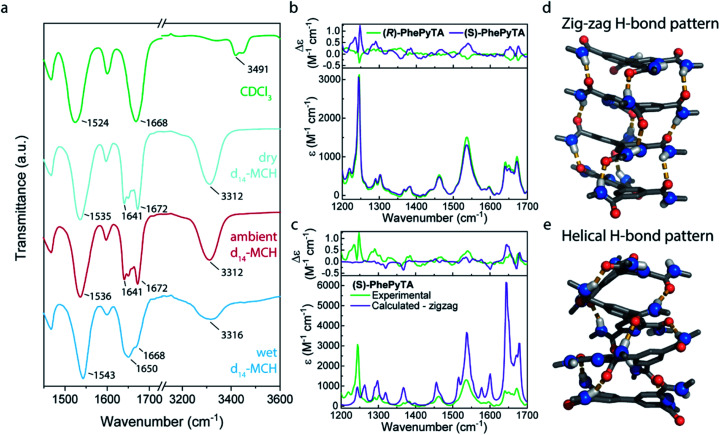
(a) FT-IR spectra of 2 mM solutions of **(S)-PhePyTA** in various solvents. (b) Experimental vibrational absorption (VA) (lower panel) and vibrational circular dichroism (VCD) (upper panel) spectra of 10 mM solutions of **(R)-** and **(S)-PhePyTA** in ambient MCH. (c) Comparison of experimental VA (lower panel) and VCD (upper panel) spectra of **(S)-PhePyTA** in ambient MCH with spectra calculated for ‘zig-zag’ aggregates. (d and e) Cartoons of the dominant ‘zig-zag’ (d) and minor helical (e) hydrogen bond pattern in **(S)-PhePyTA** aggregates.

Bulk samples of monomer **(S)-PhePyTA** show no birefringence in polarized optical microscopy (POM) images and no phase transitions in differential scanning calorimetry (DSC). Only minor shifts in the variable temperature infrared (IR) spectrum and the absence of ordered structures, as indicated by small angle X-ray scattering (SAXS) (Fig. S1–S4†), are observed. In contrast to the lack of supramolecular ordered structures in the bulk phase, solution-state SAXS experiments in ambient MCH solutions of **(S)-PhePyTA** show the formation of short cylindrical supramolecular polymers with a length of 54 nm and a radius of 6.6 nm. Upon increasing the concentration from 2 to 8 mM, the length of the polymers increases only slightly from 54 to 87 nm, indicating that the elongation constant of the polymerization might decrease with polymer length (Fig. S5†).^54^ The absence of long, one-dimensional polymers is further corroborated by AFM experiments (Fig. S6†). The weak increase in polymer length upon quadrupling the concentration suggests additional interactions that may truncate the supramolecular polymer. IR spectra show that, despite the absence of long, fibrillar structures, the structures formed by **(S)-PhePyTA** are stabilized by hydrogen bonding. Upon changing the solvent from CDCl_3_ to *d*_14_-MCH, a new band in the carbonyl stretch region emerges at 1641 cm^−1^ and the amide stretch vibrations move from 3491 to 3312 cm^−1^, respectively. The latter strongly indicates the formation of intermolecular hydrogen bonds (Fig. 2a). Interestingly, these IR bands show a dependency on the humidity of the MCH solvent, as was also observed in the strongly water-dependent polymerization of **(S)-BPTA**. However, the characteristic peak at 3558 cm^−1^, which is indicative of the incorporation of strongly hydrogen-bonded water in the supramolecular structure is absent. The broad feature between 3200 and 3600 cm^−1^ does suggest, however, that some hydrogen-bonded water may be present in the solution and around the substrate, but the water is not tightly bound to the supramolecular aggregates.

Further insights into the structure of the aggregates formed by **(S)-PhePyTA** and its enantiomer under ambient conditions at room temperature, are obtained with VCD spectroscopy in combination with DFT calculations (Fig. 2b). Under these conditions, the system is above the water-dependent transition temperature (Fig. S7†). The VCD spectra show strong features arising from coupled oscillators, thereby indicating that **(S)-PhePyTA** aggregates in an oligomeric or polymeric form of at least 2–4 molecules. A reasonable mirror symmetry between the spectra of **(S)-** and **(R)-PhePyTA** is obtained. The smaller signal intensity of **(R)-PhePyTA** can be explained by subtly different degrees of aggregation and/or enantiomeric excess. In contrast to other VCD experiments on supramolecular polymers,^55–58^ in which the bisignate VCD bands pointed to helical polymers, DFT calculations of VCD spectra of helically hydrogen-bonded **(S)-PhePyTA** did not give a good agreement with the spectra experimentally observed (Fig. 2c, e and S8†). Remarkably, VCD spectra calculated for optimized geometries in which the amides are oriented in a ‘zig-zag’ fashion with stacks in a slight helical twist (Fig. 2d) showed considerably better overlap with the observed spectra (Fig. 2c). Our calculations under vacuum conditions show that this ‘zig-zag’ pattern is considerably lower in energy (17.6 kJ mol^−1^ for the trimer structures) than the helical hydrogen bonding pattern (Fig. 2d). Combined with the experimental results, the calculations indicate that in the water-free **(S)-PhePyTA** aggregates, the classical, helical organization of the hydrogen bonds is only a minor population. Rather, most hydrogen bonds in these aggregates are organized in the ‘zig-zag’ orientation.

This dominant ‘zig-zag’ conformation is corroborated by NOESY NMR experiments of **(S)-PhePyTA** at 80 °C in *d*_14_-MCH (Fig. S9†). Here, the absence of a NOE peak between the pyridyl protons and the amides on the pyridine ring suggests the presence of an intramolecular hydrogen bond between the amides and the pyridine nitrogen.

**Fig. 3:**
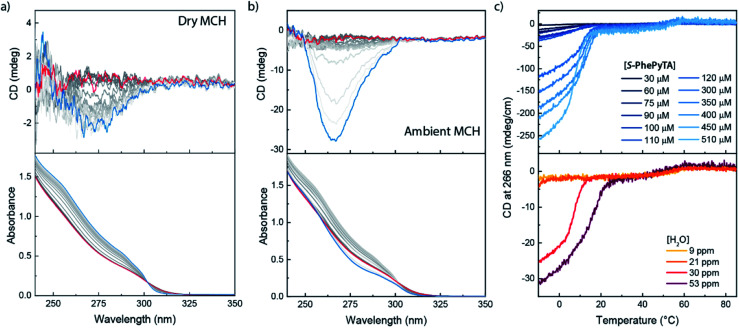
VT-CD and UV spectra recorded upon cooling 300 μM solutions of **(S)-PhePyTA** in dry (a) and ambient (b) MCH. Spectra are recorded at 5 °C intervals, with the red spectra being recorded at 85 °C and the blue spectra at −10 °C. The grayscale indicates the temperature gradient from high (light) to low (dark) temperature. (c) VT-CD traces at 266 nm for solutions containing various amounts of **(S)-PhePyTA** in MCH containing 21–31 ppm H_2_O (top panel) and 510 μM solutions containing various amounts of water (bottom panel). The sample containing 510 μM **(S)-PhePyTA** in the top panel contained 30 ppm water.

We further investigated the supramolecular architectures formed by **(S)-PhePyTA** in MCH and their dependency on the water content by variable temperature circular dichroism (VT-CD) and UV-Vis (VT-UV) spectroscopy. At 85 °C in dry MCH, 510 μM solutions of **(S)-PhePyTA** show a broad, CD-silent absorption band below 350 nm with a maximum below 230 nm (Fig. 3a). This spectrum is similar to the spectrum observed for **(S)-BPTA**, but slightly redshifted. Upon cooling the solution below 60 °C, a weak CD spectrum showing a minimum around 275 nm emerges, while the absorption spectrum narrows slightly, indicating the formation of weakly ordered helical structures, which we label **APhePyTA**. Further cooling to 5 °C does not lead to further changes in the CD or UV spectra, indicating that the aggregates do not undergo further structural changes. In contrast, when ambient MCH solutions, containing 21–32 ppm H_2_O, are cooled below 20 °C, the CD spectrum strongly increases in intensity, while retaining its shape (Fig. 3b). Concurrently, the absorption spectrum shows further sharpening, indicating a transition from the **APhePyTA** state to a second aggregate state, which we label as **BPhePyTA**.

Similar to the water-dependent supramolecular polymerization of **(S)-BPTA**, the elongation temperature of monomeric **(S)-PhePyTA** into **APhePyTA** gradually decreases as the concentration is decreased but does not show a dependency on water content (Fig. 3c, top panel). In contrast, the transition between **APhePyTA** and **BPhePyTA** is independent of concentration, yet strongly affected by the water content (Fig. 3c, bottom panel). The temperature-dependent CD intensity of **(R)-PhePyTA** gives a mirror-image cooling curve with identical transition temperatures (Fig. S10†). A van't Hoff analysis of the concentration-dependent elongation temperatures of **(S)-PhePyTA** extracted from the VT-UV traces reveals that the enthalpy and entropy changes upon formation of **APhePyTA** are −94 kJ mol^−1^ and −220 J mol^−1^ K^−1^, respectively (Fig. S11†). The enthalpy released by **(S)-PhePyTA** upon aggregation is slightly more negative than the enthalpy released by **(S)-BPTA**,^51^ indicating that the dipolar character of the phenylpyridine core may stabilize the water-free **APhePyTA** state with respect to the A state of **(S)-BPTA**. During the transition from **APhePyTA** to **BPhePyTA**, all VT-CD intensities of the samples measured increase as the solutions are cooled to temperatures as low at −10 °C, indicating that the system does not fully transform from **APhePyTA** to **BPhePyTA** in the temperature range probed. Unfortunately, the incomplete transition to **BPhePyTA** does not allow us to extract the molar ellipticity of **BPhePyTA**, which prevents meaningful fitting to a solvent-dependent mass-balance model as was done for **(S)-BPTA**.^51,53^ Thus, the VT-CD and UV results show that the monomers in the competing **APhePyTA** and **BPhePyTA** states have similar molecular arrangements, but further insights into the water-dependent competition between **APhePyTA** and **BPhePyTA** are necessary for a complete description of the aggregation.

**Fig. 4:**
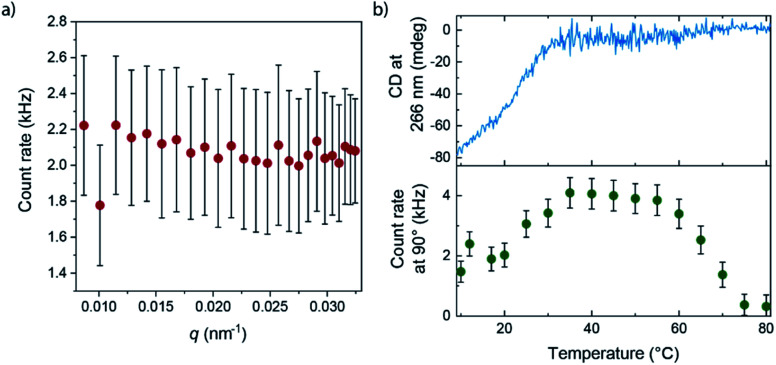
(a) Dependency of the SLS count rate at 20 °C on the scattering vector, *q*, of a 2 mM **(S)-PhePyTA** solution MCH that is saturated with water. (b) VT-CD intensity at 266 nm (top panel) and count rates at a 90° scattering angle as observed by SLS (bottom panel) of a 2 mM **(S)-PhePyTA** solution in MCH saturated with water.

More details of the differences between **APhePyTA** and **BPhePyTA** were obtained with static light scattering techniques. For this, we probed the angle and temperature-dependent-scattering of an MCH solution saturated with water and containing 2 mM **(S)-PhePyTA**. The scattering profile below 20 °C, where **BPhePyTA** is stable, does not show any significant dependency on the angle-dependent scattering vector, indicating the presence of spherical or very short aggregates (Fig. 4a and S12†). Upon heating from 20 to 40 °C, the count rate increases twofold and reaches a plateau between 40 and 60 °C. At this plateau, the angle independency is preserved over the entire *q*-range measured and is in line with the plateau observed at *q*-values below 0.05 nm^−1^ in the solution state SAXS measurements on **APhePyTA** (Fig. 4b and S12†). Upon heating above 60 °C, the sample shows concomitant decreases in scattering intensity and CD intensity and above 80 °C, the sample hardly scatters and shows no CD signal (Fig. 4b).

The concomitant changes in scattering and CD intensity provide valuable clues on the structures of the various **(S)-PhePyTA** aggregates. The initial increase in scattering intensity and decrease in CD signal upon heating the sample from 20 to 40 °C, together with the plateau observed in SAXS, indicates that the spherical or very short fibrillar structures present at room temperature, **BPhePyTA**, elongate into larger, but more disorganized fibrillar aggregates, **APhePyTA**. Interestingly water stabilizes the short fibrillar structures present at low temperature (Fig. 3c). Upon heating the samples, the water is released from the solvated **BPhePyTA** state. As **BPhePyTA** transitions to **APhePyTA**, an increase in the polymer length occurs, as has been observed before in several systems containing hydrogen-bond competitors, such as water and alcohols.^59–64^ In contrast to the previously reported systems, the solvation of the aggregates by water does not lead to strong changes in the aggregates at the molecular level, since the shape of the IR, UV and CD spectra remain the same, but only change in intensity. Upon further heating from 60 to 80 °C, the **APhePyTA** aggregates are destabilized and the scattering and CD intensities decrease as the result of the depolymerization of **APhePyTA**.

The combined VT-CD and UV, IR and scattering results show that water is acting as a chain capper for the supramolecular aggregates and thereby decreases the length of the supramolecular polymers at low temperature and high humidity. A similar effect was also observed for porphyrin-based, supramolecular polymers, in which complexation of water at helix macrodipoles at the polymer chain ends reinforces the binding of the additives to the chain end of the polymer, leading to a decrease in length that is not directly observable by spectroscopy.^44^

The combined SAXS, VCD, CD and SLS results provide us with key leads to propose a molecular picture of the aggregation behaviour of **(S)-PhePyTA**. We hypothesize that water assembles at the polymer–solvent interface and thereby subtly changes the polarity of the environment of the **(S)-PhePyTA** supramolecular polymer. The presence of water stabilizes the dipoles of the electron-poor **(S)-PhePyTA** core that would otherwise not be stabilized. As a result of this stabilization, the aggregates break up and decrease their size. Concurrently, the increased degrees of freedom in the smaller particles allow the molecules to organize better, leading to an increase of the intensity of the CD spectrum at low temperatures and high-water contents. Thus, the combination of spectroscopy and scattering results reveal that water decreases the size and disorder of the supramolecular aggregates of **(S)-PhePyTA**, while the morphology in the aggregates remain relatively unchanged.

### BiPyTA assembly is dominated by kinetic traps

We next investigated **(S)-BiPyTA**, the bipyridyl analogue of **(S)-BPTA** (Fig. 1); a biaryl with an even lower electron density in the aromatic core. The bulk properties of **(S)-BiPyTA** are completely different from those of its **PhePyTA** analogues. DSC traces show a melting temperature around 165 °C while SAXS and POM analyses show the presence of crystalline, columnar rectangular mesophases that are stable up to at least 125 °C (Fig. S1–S3†). Similarly, VT-IR measurements in the bulk indicate the presence of a hydrogen-bonded amide, which is disrupted around 165 °C (Fig. S4†). These results show, in contrast to the amorphous bulk morphology of **(S)-PhePyTA**, that the bipyridine core with its four amides directs the bulk organization of **(S)-BiPyTA** into ordered, crystallization-driven structures (Fig. S13†).

**Fig. 5:**
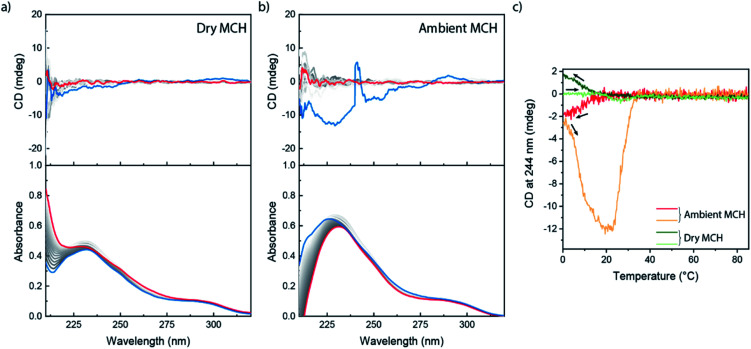
VT-CD (top panels) and VT-UV (bottom panels) spectra of 15 μM solutions of **(S)-BiPyTA** in MCH containing 10 (a) and 23 (b) ppm water. The spectra were collected with a cooling rate of 1 °C min^−1^ at 5 °C intervals, with the red spectra recorded at 85 °C and the blue spectra at 5 °C. The grayscale indicates the temperature gradient from high (light) to low (dark) temperature. (c) VT-CD traces of 15 μM solutions of **(S)-BiPyTA** in ambient (red traces) and dry (green traces) MCH upon cooling (dark shades) and subsequent heating (light shades).

The study of the self-assembly of **(S)-BiPyTA** in solution was considerably impaired by its low solubility in alkanes, such as MCH, heptane and decalin. Saturated solutions, which contain approximately 20 μM **(S)-BiPyTA**, readily precipitated at low temperatures, making solution-state SAXS, IR and SLS measurements as well as VT-CD and UV spectroscopy difficult. The UV spectrum of 15 μM **(S)-BiPyTA** solutions in dry MCH at 85 °C displays a CD-silent absorption maximum at 228 nm with a shoulder around 295 nm (Fig. 5a). Upon cooling, a very weak CD spectrum emerges, displaying a slightly positive peak around 300 nm and a slightly negative peak below 265 nm. In ambient MCH, a more intense, but highly irreproducible CD spectrum was obtained (Fig. 5b). This indicates a better solubility of the compound in the presence of water in MCH, but the irreproducibility also illustrates the presence of kinetic traps in the **(S)-BiPyTA** assembly process. The trapping of **(S)-BiPyTA** in dry and ambient MCH was also apparent in the VT-CD traces that show trends in hysteresis typically observed for kinetically trapped states (Fig. 5c).^65^ Unfortunately, slower cooling did not resolve the kinetic trapping and the formation of stable and ordered supramolecular polymers was impossible (Fig. S14†). **(S)-BiPyTA** appears to have a strong tendency to crystallize. Nonetheless, as was also observed for an insoluble achiral derivative of **(S)-BPTA**, in which the chiral alkyl tails were substituted by a linear octyl tail, the insolubility of the pure compound does not preclude coassembly with other compounds to form supramolecular copolymers.^53^

### Copolymerizations

Although both **(S)-PhePyTA** and **(S)-BiPyTA** have a low tendency to form ordered helical supramolecular polymers of considerable length, the electron-poor nature of their aromatic cores may drive copolymerization with **(S)-BPTA**. The latter polymerizes at concentrations as low as 10 μM and remains soluble up to mM concentrations.^51^ As such, the poorly polymerizing additives may modulate the morphology of the **(S)-BPTA** copolymers. The copolymerizations were conducted in dry solvents to exclude interference from water, which can considerably hinder copolymer analyses.^51^ To this end, we first investigated the effect of phenylpyridine derivative **(S)-PhePyTA** as an additive to the polymerization of **(S)-BPTA**.

### **(S)-PhePyTA** modulates the length of **(S)-BPTA**-based polymers

**Fig. 6:**
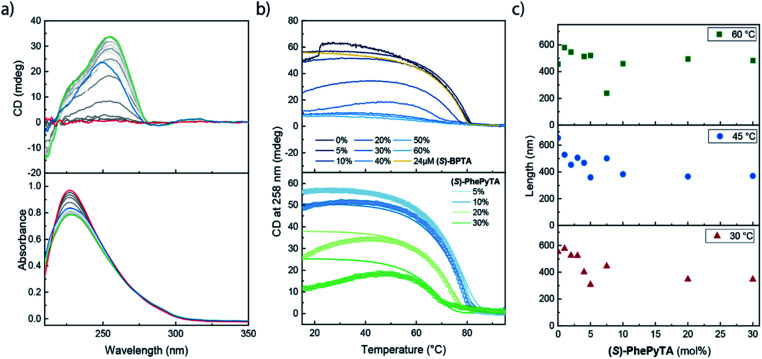
(a) Spectra of a sample containing 24 μM **(S)-BPTA** and 6 μM **(S)-PhePyTA** in dry MCH. The spectra were recorded at 5 °C intervals with the red, green and blue spectra collected at 95, 40 and 5 °C. Spectra are colour coded on a grayscale going from light (95 °C) to dark (5 °C). (b) Top panel: VT-CD traces of samples containing various ratios of **(S)-BPTA** and **(S)-PhePyTA** at a total concentration of 30 μM (blue traces) and a 24 μM sample of **(S)-BPTA** (yellow trace) in MCH containing less than 10 ppm H_2_O. Bottom panel: experimental (symbols) and fitted (lines) VT-CD traces of 30 μM mixtures of **(S)-BPTA** and **(S)-PhePyTA** in dry MCH. (c) SLS-derived lengths of the **(S)-BPTA**:**(S)-PhePyTA** copolymers in MCH containing less than 10 ppm H_2_O upon addition of varying amounts of **(S)-PhePyTA** at a fixed **(S)-BPTA** concentration of 100 μM.

VT-CD and UV measurements on 30 μM samples containing various ratios of **(S)-BPTA** and **(S)-PhePyTA** all showed a CD-silent UV maximum at 227 nm above 80 °C (Fig. 6a and S15†). Upon cooling below 80 °C, a CD spectrum reminiscent of water-free homopolymers of **(S)-BPTA** emerges, showing a maximum at 258 nm. Further cooling does not lead to considerable changes in the spectrum, except for the amplitude. Solutions containing low fractions (between 0 and 10 mol%) of **(S)-PhePyTA** additive show no significant hysteresis and an elongation temperature around 81 °C is observed (Fig. 6b, top panel and S16†). Upon further cooling the CD intensities observed for solutions of copolymers containing up to 10 mol% **(S)-PhePyTA** reach a maximum between approximately 20 and 40 °C. Below this temperature, the intensity gradually decreases, indicating that the helical organization in the polymers weakens or less monomers are polymerized. In contrast, for solutions containing more than 10 mol% **(S)-PhePyTA**, the elongation temperature increasingly shifts to lower temperature, while the maximum CD intensity decreases considerably. For samples that contain an **(S)-PhePyTA** mole fraction of 40 mol% or more, hardly any ordered polymers are formed, while the absorption spectra at room temperature retain a relatively low intensity. As no scattering in the UV spectrum at higher wavelengths is observed (Fig. S15†), the low absorbance most likely indicates that the biaryl monomers are not molecularly dissolved but are aggregated in a CD-silent aggregate.

A comparison of solutions containing 30 μM biaryl-based mixtures consisting of 80 mol% **(S)-BPTA** and 20 mol% **(S)-PhePyTA** with solutions containing only 24 μM **(S)-BPTA** sheds further light on the role of the phenylpyridine additive. The latter solution shows an elongation temperature of 80 °C and has a CD intensity of 53 mdeg, in line with our previous report.^51^ The 30 μM copolymer solution, which also contains 24 μM **(S)-BPTA** shows, however, an elongation temperature of approximately 78 °C and reaches a maximum CD value of only 34 mdeg around 40 °C. With the van't Hoff equation and the thermodynamic parameters of the **(S)-BPTA** polymerization, we calculate that **(S)-BPTA** homopolymers elongate at 78 °C at a monomer concentration of 20 μM. This concentration is very close to the excess of **(S)-BPTA** in the copolymerization, strongly suggesting that **(S)-PhePyTA** sequesters **(S)-BPTA** from the polymerization in an equimolar ratio. This role of **(S)-PhePyTA** as a sequestrator explains the VT-CD of all samples containing up to 30 mol% of **(S)-PhePyTA** (Fig. 6b), which further corroborates the equimolar sequestration of **(S)-BPTA** by **(S)-PhePyTA**.

Additional thermodynamic insight into the sequestration of **(S)-BPTA** by **(S)-PhePyTA** was obtained by fitting a mass-balance model that describes the respective homopolymerization and sequestration to the VT-CD traces (full details in the ESI†). The fits of the mixtures containing up to 30 mol% **(S)-PhePyTA** are in good agreement with the experimental data (Fig. 6b, lower panel). From the fits, upper and lower bounds on the value of the enthalpy and entropy of the sequestration event are obtained. The model is in good agreement with the data for several sets of thermodynamic values with enthalpy values between −80 and −107 kJ mol^−1^ and entropy values between −89 and −169 J mol^−1^ K^−1^, (Fig. S17†). Unfortunately, the model is not able to give clear indications on enthalpic differences between the respective homo- and hetero-interactions between **(S)-BPTA** and **(S)-PhePyTA**. However, the upper bound for the entropic penalty of −169 J mol^−1^ K^−1^ indicates that the entropic penalty of the hetero-interaction is less than the entropic penalty of ordering the monomers in the homopolymers, suggesting that the sequestered complex is rather disorganized. This disorganized nature of the sequestered species is in agreement with the lack of a strong CD signal. Interestingly, when we tried to fit the entire dataset, which includes the VT-CD traces of samples containing 40, 50 and 60 mol% **(S)-PhePyTA**, the model incorrectly predicts an elongation of **(S)-BPTA** at low temperatures, rather than around 60 °C (Fig. S18†). The model's lower elongation temperatures indicate that at higher **(S)-PhePyTA** ratios and low temperatures, there may be a sufficiently large thermodynamic driving force for **(S)-PhePyTA** to act as an intercalator or chain capping agent. Thus, while **(S)-PhePyTA** may act as chain capper or intercalator at low temperatures, the VT-CD results show that, at 60 °C, the phenylpyridine-based additive acts primarily as a sequestrator of **(S)-BPTA**.

To confirm the role of an additive in supramolecular copolymerizations, spectroscopic studies were complemented by scattering techniques to reveal information on the size of the copolymers.^23^ To this end, we conducted multi-angle SLS measurements at 30, 45 and 60 °C on dry MCH solutions containing 100 μM **(S)-BPTA** and varying amounts of **(S)-PhePyTA** (Fig. 6c). After fitting the scattering results to a cylindrical model, the experiments show that at all temperatures the length of the supramolecular polymers gradually decreases as the phenylpyridine additive is added. This gradual decrease in polymer length upon addition of **(S)-PhePyTA** is a hallmark of the sequestrating role of **(S)-PhePyTA**. In contrast, a sharp decrease would be indicative of a chain capper, whereas a subtle length increase would indicate copolymerizing or intercalating additives.^23^ Furthermore, when more than 10 mol% **(S)-PhePyTA** is present in the system, the slope of the scattering profiles decreases at high *q*-values (Fig. S19–S21†). This change in slope suggests the presence of a second population of non-cylindrical aggregates next to the cylindrical supramolecular polymers, further corroborating the predominantly sequestrating role of **(S)-PhePyTA**. Surprisingly, the phenylpyridine core of the **(S)-PhePyTA** does not lead to the efficient formation of copolymers with **(S)-BPTA**. Rather, the phenylpyridyl core directs the formation of small, sequestrating dimers or oligomers through which the chain length of the **(S)-BPTA** polymers can be tuned in a controlled manner.

### **(S)-BiPyTA** modulates **(S)-BPTA**-based polymers as a function of temperature

**Fig. 7:**
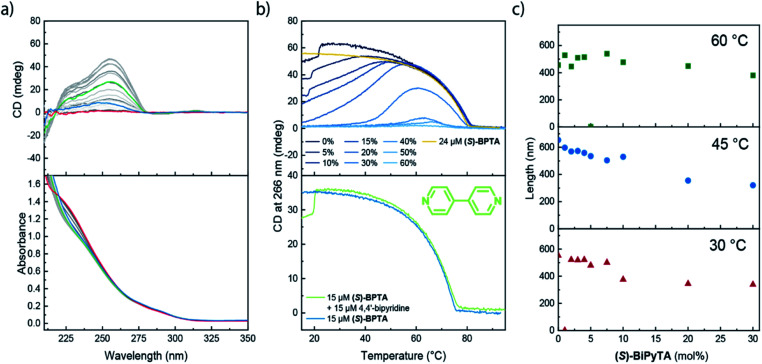
(a) Spectra of a sample containing 24 μM **(S)-BPTA** and 6 μM **(S)-BiPyTA** in dry MCH. The spectra were recorded at 5 °C intervals with the red, green and blue spectra collected at 95, 40 and 5 °C. Spectra are colour coded on a grayscale going from light (95 °C) to dark (5 °C). (b) Top panel: VT-CD traces of samples containing various ratios of **(S)-BPTA** and **(S)-BiPyTA** at a total concentration of 30 μM (blue traces) and a 24 μM sample of **(S)-BPTA** (yellow trace) in MCH containing less than 10 ppm H_2_O. Bottom panel: VT-CD traces of MCH solutions containing 15 μM **(S)-BPTA**, and 15 μM of both **(S)-BPTA** and 4,4′-bipydirine. (c) SLS-derived lengths of the **(S)-BPTA**:**(S)-BiPyTA** copolymers in MCH containing less than 10 ppm H_2_O upon addition of varying amounts of **(S)-BiPyTA** at a fixed **(S)-BPTA** concentration of 100 μM.

Next, we investigated the role of **(S)-BiPyTA** in the polymerization of **(S)-BPTA** using an identical approach as employed for **(S)-PhePyTA**. The VT-UV and CD spectra show that the absorption spectra gradually change upon increasing the molar fraction of **(S)-BiPyTA** in 30 μM solutions of **(S)-BPTA** and **(S)-BiPyTA**. The VT-CD spectra for all copolymerizations show identical spectral shapes, which indicates that the microscopic structure of the ordered aggregates present in solution remains identical to the structure of the **(S)-BPTA** homopolymers (Fig. 7a and S22†). The VT-CD intensity at 258 nm shows that at 30 μM total monomer concentration, the copolymers containing up to 30 mol% of **(S)-BiPyTA** all elongate at 81 °C and minimal hysteresis and water dependency is observed (Fig. 7b, S23 and S24†). Together with the CD intensities around the elongation temperature, which are similar to **(S)-BPTA** homopolymers, these results show that **(S)-BiPyTA** readily copolymerizes with **(S)-BPTA**. When the temperature is further decreased, the CD intensity reaches a maximum, which occurs at higher temperatures for higher molar ratios of **(S)-BiPyTA** in the copolymers. After reaching a maximum, the CD intensity gradually decreases as the temperature is further decreased and solutions containing 20 mol% or more **(S)-BiPyTA** do not show any CD intensity at 20 °C anymore. For solutions containing more than 30 mol% **(S)-BiPyTA**, the elongation temperatures become increasingly lower, while solutions containing 60 mol% do not show any considerable formation of supramolecular polymers at all. In contrast to the results obtained for **(S)-PhePyTA**, the VT-CD traces of the **(S)-BPTA**:**(S)-BiPyTA** solutions suggest that **(S)-BiPyTA** copolymerizes with **(S)-BPTA** at high temperatures, while at low temperatures a similar destabilization of the polymers occurs as observed for **(S)**-**PhePyTA**.

The incorporation of small fractions of **(S)-BiPyTA** into supramolecular polymers of **(S)-BPTA** at high temperatures is corroborated by SLS measurements. At 60 °C, the supramolecular polymers do not show a noticeable decrease in polymer length when **(S)-BiPyTA** is added to 100 μM solutions of **(S)-BPTA** (Fig. 7c). In addition, the scattering profiles of the samples at 60 °C containing **(S)-BiPyTA** fractions can be described with a single cylindrical model (Fig. S25†), suggesting that only a single polymer species is present. Together with the spectroscopic data, this result points to a predominant intercalating role of **(S)-BiPyTA** in which the additive readily incorporates into the **(S)-BPTA**-based polymers at temperatures of 60 °C. However, due to destabilizing dipolar interactions, **(S)-BiPyTA** does not form contacts with other **(S)-BiPyTA** monomers, and this results in only a modest decrease in polymer length at low additive ratios. At lower temperatures, where the CD intensity of the **(S)-BPTA**:**(S)-BiPyTA** mixtures decreases, SLS shows a gradual decrease in the polymer length, while the scattering profiles of samples containing 10 mol% **(S)-BiPyTA** or more show a change in slope at high *q*-values (Fig. 7c, S26 and S27†), indicating that other aggregates are formed. Thus, the SLS results confirm that at low temperatures, **(S)-BiPyTA** most likely functions as a sequestrator of **(S)-BPTA**.

**Fig. 8:**
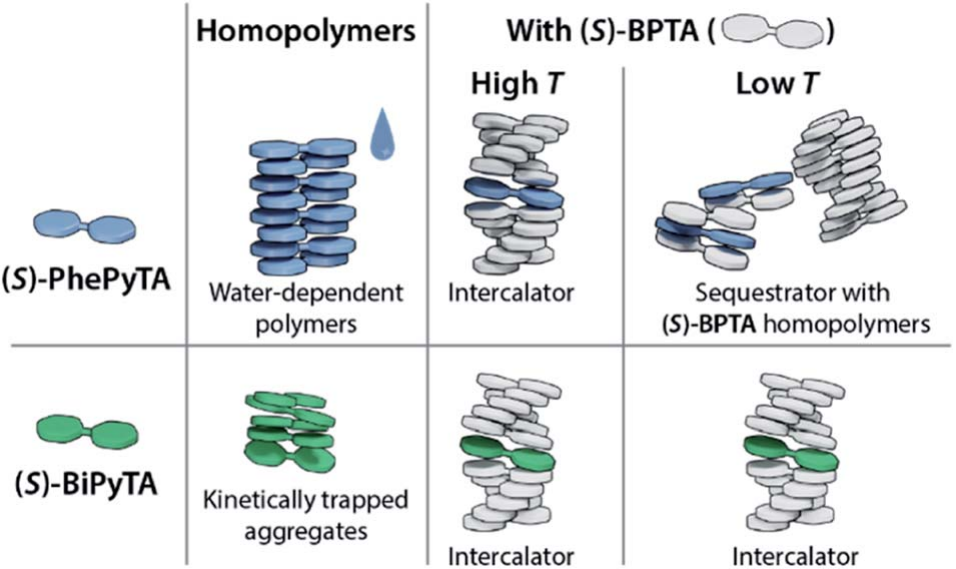
Cartoon representation of the aggregation behaviour of **(S)-PhePyTA** and **(S)-BiPyTA** in the absence and presence of **(S)-BPTA**.

To gain a more detailed insight into the driving forces leading to copolymerization of **(S)-BiPyTA** with **(S)-BPTA** at higher temperatures, we also studied a solution containing 15 μM **(S)-BPTA** and 15 μM 4,4′-bipyridine (Fig. 7b, lower panel). The 4,4′-bipyridine additive features a similar electron-poor aromatic core but does not possess the ability to form intermolecular hydrogen bonds with the amides of **(S)-BPTA**. When comparing the VT-CD traces of this mixture with the VT-CD trace of a 15 μM **(S)-BPTA** homopolymerization, no significant differences can be observed apart from changes in intensity that are due to different levels of humidity in the solution. Thus, dipolar interactions alone are not sufficient for the intercalation into the **(S)-BPTA**-based polymers. Instead, the hydrogen-bonding ability of the additive appears key to further drive the incorporation of **(S)-BiPyTA** into the polymers. As such, we conclude that the subtle changes in hydrogen bonding interactions and dipolar character of the **(S)-PhePyTA** and **(S)-BiPyTA** additives can lead to profound changes in supramolecular behaviour (Fig. 8).

## Conclusions

In this work, we have studied the supramolecular aggregation in methylcyclohexane (MCH) of phenylpyridine- and bipyridine-based analogues of the previously studied biphenyltetracarboxamide **(S)-BPTA**. Unlike the long and ordered supramolecular polymers formed by **(S)-BPTA**, the phenylpyridine **(S)-PhePyTA** forms short, disorganized fibers, while the bipyridine **(S)-BiPyTA** forms kinetically trapped, ill-defined supramolecular aggregates. With a combination of spectroscopic and scattering techniques, it was found that water controls the chain length of the **(S)-PhePyTA** aggregates in ambient MCH and is responsible for the small size of these aggregates. With the decreasing solubility and ordering in the supramolecular polymerization upon increasing the electron deficiency of the aromatic core, the biaryl monomers highlight the balance between solubility and aggregation driving force that is required to form ordered supramolecular polymers.

Although the pyridine-based molecules do not form long, ordered supramolecular polymers by themselves, copolymerization experiments show that they can be used to control the length and stability of **(S)-BPTA**-based polymers. By sequestrating **(S)-BPTA** monomers at high temperatures, **(S)-PhePyTA** suppresses the length and elongation temperature of **(S)-BPTA** homopolymers. At lower temperatures, the weak decrease of the polymer length suggests that the additive further destabilizes the polymers through intercalation. In contrast, **(S)-BiPyTA** intercalates into the polymers already at high temperatures, giving subtle control over the length of the **(S)-BPTA**-based copolymers.

These observations illustrate the delicate energetic balances encountered in these systems, as was also observed for other biphenyl systems.^51^ With this, we provide further insights into the dramatic effects that small structural variations can have on supramolecular systems. To gain a further understanding of these phenomena, a combination of thorough experimental and computational analyses is crucial. We envision that such systematic studies will lead to further control over supramolecular materials for a wide range of biomedical and opto-electronical applications. By acting as synthetic analogues of biochemical chaperones, such additive molecules are thereby a valuable tool in the expanding toolbox of non-covalent synthesis.

## Data availability

All relevant data is presented in the paper and ESI.† Raw data is available upon request by email to E. W. M. The Matlab code used for fitting the mass-balance model can be found at https://www.github.com/mathijs-m/BPTA_polymerization_sequestration.

## Author contributions

E. W. M. and T. I. devised the project. M. F. J. M. and T. I. developed the research and performed the majority of the experiments and writing. M. F. J. M. carried out all the calculations. M. A. J. K. analyzed the stacking fashion of PhePyTA aggregates *via* DFT calculation and VCD experiments. B. A. G. L. measured X-ray diffraction and DSC. E. W. collected VT-IR spectra. L. N. J. W. performed SAXS experiment. All authors have given approval to the final version of the manuscript.

## Conflicts of interest

There are no conflicts to declare.

## Supplementary Material

SC-012-D1SC03974A-s001

SC-012-D1SC03974A-s002

SC-012-D1SC03974A-s003

SC-012-D1SC03974A-s004

SC-012-D1SC03974A-s005

SC-012-D1SC03974A-s006

SC-012-D1SC03974A-s007
