# Supplementary material for: Temperature-dependent modulation by biaryl-based monomers of the chain length and morphology of biphenyl-based supramolecular polymers
Source: Chem Sci. 2021 Sep 6;12(39):13001–12. doi: 10.1039/d1sc03974a (PMC8513997; doi:10.1039/d1sc03974a)
Supplement: SC-012-D1SC03974A-s007 [file SC-012-D1SC03974A-s007.pdf]

Electronic Supplementary Material (ESI) for Chemical Science.
This journal is © The Royal Society of Chemistry 2021

Temperature-dependent modulation by biaryl-based monomers of the chain length and morphology of biphenyl-based supramolecular polymers

Tomokazu Iseki[1,2]†, Mathijs F.J. Mabesoone[1,3]†, Mark A. J. Koenis[4], Brigitte A.G. Lamers[1], Elisabeth Weyandt[1], Lafayette N.J. de Windt[1], Wybren Jan Buma[4,5], Anja R.A. Palmans[1], E.W. Meijer[1*]

[1]Institute for Complex Molecular Systems and Laboratory of Macromolecular and Organic Chemistry, Eindhoven, University of Technology, 5600 MB Eindhoven, The Netherlands
[2]Material Science Research Laboratory, Kao Corporation, Wakayama-shi, Wakayama 640-8580, Japan
[3]Institute of Microbiology, Eidgenössische Technische Hochschule Zürich, Vladimir-Prelog-Weg 4, 8093 Zurich, Switzerland
[4]Van't Hoff Institute for Molecular Sciences, University of Amsterdam, Science Park 904, 1098 XH Amsterdam, The Netherlands
[5]Institute for Molecules and Materials, FELIX Laboratory, Radboud University, Toernooiveld 7c, 6525 ED Nijmegen, The Netherlands
†Equal contributions
* Corresponding author: e.w.meijer@tue.nl.

Supplementary Information

General methods and materials

All starting materials were purchased from commercial suppliers and used as received, unless noted otherwise. All solvents were purchased from Biosolve, spectroscopy grade methylcyclohexane (MCH) was supplied from Acros Organics, deuterated methylcyclohexane was purchased from abcr GmbH and the deuterated solvents were purchased from Cambridge Isotopes Laboratories. (*S*)- and (*R*)-3,7-Dimethyloctan-1-amine were prepared as previously reported[1,2] from (*S*)-citronellol (*ee*=98.4%) and (*R*)-citronellol (*ee*≥99%) that were purchased from Takasago and Sigma-Aldrich, respectively. Dry solvents were obtained with an MBRAUN Solvent Purification System (MB-SPS). All chemicals were purchased from commercial sources and used as received unless otherwise specified. Spectroscopy grade MCH was used in the UV-Vis and CD experiments. Flash column chromatography was performed on a Biotage Isolera One system equipped with an ultraviolet detector.

^{1}H and ^{13}C NMR measurements were performed on a Bruker Avance 3 HD NanoBay spectrometer (^{1}H NMR 400 MHz, ^{13}C NMR 100 MHz) and measured in $CDCl_3$. The chemical shifts are reported relative to the residual $CHCl_3$ signals at 7.26 and 77.23 ppm for ^{1}H and ^{13}C, respectively. Abbreviations used are d = doublet, dd = double doublet, m = multiplet, p = pentet, s = singlet, t = triplet and q = quartet. NMR measurements in d_{14}-MCH were performed on an Oxford NMR AS500 spectrometer equipped with a 5 mm ID AutoX ID PFG Probe operating at 80 °C.

MALDI-TOF-MS was acquired using a Bruker Autoflex Speed MALDI-TOF using α-cyano-4-hydroxycinnamic acid (CHCA) or *trans*-2-[3-(4-tert-butylphenyl)-2-methyl-2-propenylidene]malononitrile (DCTB) as matrices. UV-Vis and CD spectra were recorded on a Jasco J-815 spectrometer equipped with a Jasco MPTC-490S Peltier-type or PFD-425S/15 temperature controller. The sample holder was purged with nitrogen. Quartz cuvettes (Hellma Analytics) with path lengths of 10 mm and 1 mm equipped with a tightly sealable screw cap were used, unless stated otherwise. Before all cooling and heating traces were acquired, samples were equilibrated at the initial temperature for 15 min. Water concentrations were determined directly before sample preparation through Karl-Fisher titrations with Mettler Toledo C30 Coulometric KF Titrator loaded with CombiCoulomat Frit KF reagent. All titrations were performed in triplicate on approximately 0.5 gram of the solvent. After sample preparation, the samples were tightly screwed in screw-capped, Teflon lined vials to ensure minimal exchange of moisture between the solvent and the surroundings. Dry MCH was prepared in a nitrogen-filled glovebox after leaving neat MCH in the box overnight, yielding MCH with <10 ppm water, as determined by Karl-Fisher titration. Wet MCH was prepared by layering MCH (around 20 ml) over water (around 1 ml) that was purified with an EMD Milipore Mili-Q Integral Water Purification System. After allowing to set for at least 2 h, wet MCH was carefully withdrawn with a syringe from the top layer without disturbing the bottom water phase. Wet samples were prepared on the benchtop; care must be taken to minimize exposure of the solvent to the atmosphere by quickly sealing the sample vials. Wet MCH transferred in this way contained approximately 60 ppm water, as determined by Karl-Fisher titration.

Infrared spectroscopy was measured using a PerkinElmer FT-IR Spectrum Two equipped with a Perkin-Elmer UATR Two. Solution FT-IR measurements were performed using a liquid cell with an optical path length of 0.5 mm. For the solution samples, first a background of the appropriate solvent was measured. All spectra were measured at room temperature from 400 cm^{-1} to 4000 cm^{-1} and were averaged over 64 scans. Variable Temperature IR (VT-IR) spectra were recorded on a Bruker Tensor 27 spectrometer with a Pike GladiATR 210 module. The samples were heated from room temperature to 205 °C with a heating ramp of 5 °C/min and full spectra recorded every 5 °C. DSC was performed on a TA Instruments Q2000 system. About 2–4 mg of material was prepared in hermetically sealed aluminum pans and characterized.

For AFM measurements, a sample solution containing 15 μM of the biaryl derivative was prepared using dry MCH in the glovebox. The sample prepared with dry MCH was drop casted on ZYA grade HOPG in the glovebox. AFM was performed using an Asylum Research MFP-3D system in non-contact tapping mode. Images were processed using Gwyddion.

POM images were obtained with a Jenaval polarization microscope equipped with a Linkam THMS 600 temperature controller. Images were captured with crossed polarizers. The samples were positioned on a coverslip and mounted directly on the Linkam heating stage. Micrographs were taken at specific temperature which was starting from the molten (isotropic) state.

DSC data was collected on a DSC Q2000 from TA instruments, calibrated with an indium standard. The samples (4–8 mg) were weighed directly into aluminum pans and hermetically sealed. The samples were initially heated to 180 °C and then subjected to two cooling/heating cycles from −50 °C to 180 °C with a rate of 10 K min^{-1}. The data that is presented, represents the second heating and/or cooling cycle.

SAXS measurements were performed on an instrument from Ganesha Lab. The flight tube and sample holder are all under vacuum in a single housing, with a GeniX-Cu ultralow divergence X-ray generator. The source produces X-rays with a wavelength (λ) of 0.154 nm and a flux of 1 × 108 ph s^{-1}. Samples were put inside 1 mm diameter glass capillaries, and annealed by heating above the (expected) melting point and slow cooling to room temperature. Scattered X-rays were captured on a 2-dimensional Pilatus 300K detector with 487 × 619 pixel resolution. Samples were measured in MAXS mode for 1200 seconds and WAXS mode for 300 seconds. The sample-to-detector distance was 0.084 m (WAXS mode) or 0.431 m (MAXS mode). The instrument was calibrated with diffraction patterns from silver behenate. The raw data files were calibrated and reduced to 1-D data with the SAXSGui software. MAXS and WAXS regions were merged into a single data file using the SAXSutilities software package.

SLS measurements were recorded on an ALV/CGS-3 MD-4 compact goniometer system equipped with a multiple tau digital real time correlator (ALV-7004) and a solid-state laser (λ = 532 nm; 40 mW). Scattering intensity was detected over an angular range of 30° to 150° with steps of 5°, and averaged over at least 10 runs of 2 seconds per angle. The samples were measured in light scattering tubes with an outer diameter of 1 cm. As a reference, samples of only the corresponding solvent and only toluene were measured. Solvents were filtered with 0.1 μm syringe filter, **(*S*)-PhePyTA** solution was filtered with 0.2 μm syringe filter, and corresponding **(*S*)-BPTA** blend solutions were filtered with 1.0 μm syringe filter (PTFE membrane). The data was filtered using a modified Z-score, in which datapoints with a modified Z-score above 2 were discarded. A refractive index of 1.422 was used for the MCH solvent. Lengths of the supramolecular polymers were obtained by fitting the angle-dependent count rates with a cylindrical model using the SasView program.[3] The scale, background and length parameters were used as fit parameters, while the radius was fixed at 65.8 Å, the value obtained from solution state SAXS. Several initial starting values in the optimization of the polymer length were used and the optimized value with lowest fitting error is reported.

Vibrational Absorption (VA) and Vibrational Circular Dichroism (VCD) spectra were obtained using a Biotools ChiralIR-2X spectrometer. The two PEMs were set to a central frequency of 1400 cm^{-1}. The samples of both enantiomers were prepared by making a solution of 10 mM **(*S*)-PhePyTA** in MCH-d_{14}. Subsequently the sample was injected into a sealed BaF_2 cell with a path length of 205 μm and were measured for 48 hours in blocks of 30 minutes that were afterwards averaged. Outlying measurement blocks were omitted in this averaging. Baselines were obtained from spectra of pure MCH-d_{14}.

Density Functional Theory (DFT) calculations were performed with the ADF software suite of programs[4] at a BP86/TZP level of theory. The calculations have been performed using the ExactDensity setting to improve the computation of intermolecular interactions. Several possible stacking structures of 3 and 4 **(*S*)-PhePyTA** molecules have been drawn by hand which were then optimized using the Quild optimization routines.[5] Subsequently the VA and VCD intensities[5] calculated at the optimized geometry were broadened with a Lorentzian function with a full width at half maximum of 8 cm^{-1}. To aid the direct comparison to the experimental spectrum the frequencies have been corrected using the scaling method developed by Shen *et al.*[7]

Synthetic procedures

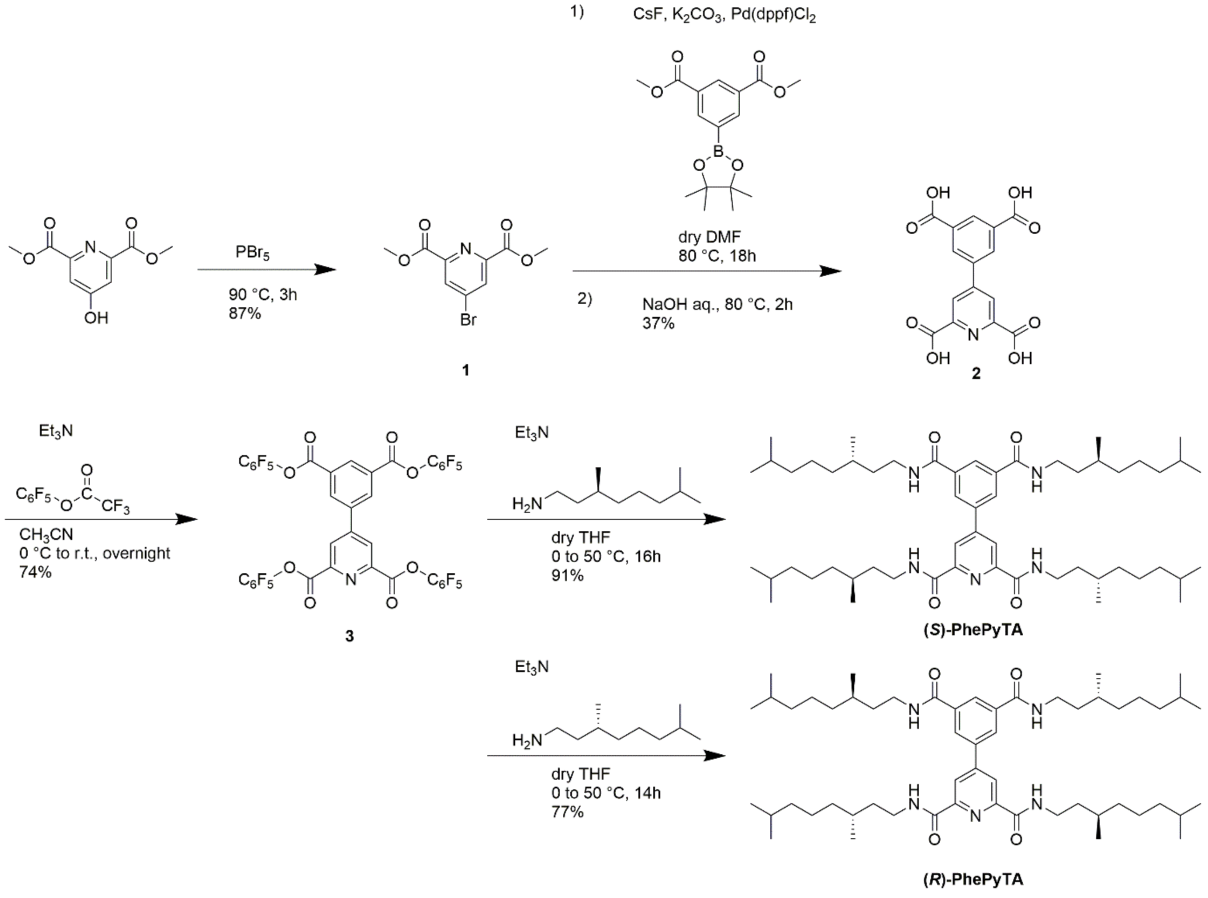

Scheme S1 Synthetic route to **(*S*)-PhePyTA**.

4-Bromopyridine-2,6-dicarboxylic acid dimethyl ester (1)
4-Hydroxypyridine-2,6-dicarboxylate dimethyl ester (1.20 g, 5.7 mmol) and phosphorus pentabromide (3.62 g, 8.4 mmol) were charged in a Schlenk flask and heated for 3 h at 90 °C. The dark brown solution was cooled to 0 °C. Then, $CHCl_3$ (20 mL) and MeOH (30 mL) were added. The precipitated solid was filtered off. After the solvents were evaporated, this solid was dissolved in $CHCl_3$ and washed with 1M Na_2CO_3 followed by brine. The organic phase was dried over $MgSO_4$ and filtered. The solvent was removed by rotary evaporator and the residue was recrystallized from MeOH. The needle-like white crystals were collected by vacuum filtration and dried under vacuum at room temperature for at least 2 h to afford compound **1** (1.35 g, yield: 87%). ^{1}H NMR (400 MHz, $CDCl_3$): δ 8.47 (s, 2H), 4.04 (s, 6H). ^{13}C NMR (100 MHz, $CDCl_3$): δ 164.0, 149.1, 135.1, 131.3, 53.5.

4-(3,5-Dicarboxyphenyl)pyridine-2,6-dicarboxylic acid (2)

Compound **1** (0.54 g, 2.0 mmol) and 5-(4,4,5,5-tetramethyl-1,3,2-dioxaborolan-2-yl)isophthalate (0.76 g, 2.4 mmol) were dissolved in dry DMF (20 mL). After stirring for 10 min, CsF (0.60 g, 3.9 mmol), K_2CO_3 (0.54 g, 3.9 mmol) and dry DMF (10 mL) were added to the solution. The mixture was stirred for 25 min at room temperature. Then Pd(dppf)Cl_2 (4.0 mg, 5.5 µmol) was added and the mixture was heated at 80 °C for 18 h. The reaction mixture was filtered and washed with THF. After the THF in the filtrate was evaporated, water (150 mL) was poured into the remaining DMF solution. The formed white precipitate was collected by vacuum filtration. The crude product was dissolved in $CHCl_3$ and purified with silica gel column chromatography (eluent: $CHCl_3$/AcOEt = 92/8 – 80/20vol%) to afford dimethyl 4-(3,5-bis(methoxycarbonyl)phenyl)pyridine-2,6-dicarboxylate as a white solid. In the last step, the dimethyl esters were hydrolyzed by stirring a suspension of the diester in 1 M NaOH (40 mL) at 80 °C for 2 h. The solution was cooled with an ice bath and acidified to pH 1 by adding 1 M HCl. The formed precipitate was collected by vacuum filtration and dried under vacuum at 50 °C for at least 12 h to afford compound **2** (0.24 g, yield: 37%).
^{1}H NMR (400 MHz, DMSO): *δ* 8.77 (s, 1H), 8.67 (s, 4H).

Bis(perfluorophenyl) 4-(3,5-bis((perfluorophenoxy)carbonyl)phenyl)pyridine-2,6-dicarboxylate (3)

Compound **2** (0.22 g, 0.7 mmol) was dissolved in a mixture of triethylamine (0.55 g, 5.4 mmol) and acetonitrile (15 mL) and cooled in an ice bath. Pentafluorophenyl trifluoroacetate (1.14 g, 4.1 mmol) in acetonitrile (1 mL) was added dropwise to the solution that was stirred at 0 °C. After all was added, the ice bath was removed, and the reaction mixture was stirred for overnight at room temperature. The formed white precipitate was collected by vacuum filtration and washed with cooled acetonitrile. The product was dried under vacuum at 50 °C for at least 12 h to afford compound **3** (0.50 g, yield: 74%). ^{1}H NMR (400 MHz, $CDCl_3$): *δ* 9.18 (t, 1H), 8.90–8.86 (m, 4H). ^{19}F NMR (376 MHz, $CDCl_3$): *δ* −151.6 (m, 4F), −152.11 (m, 4F), −156.29 (m, 4F), −161.36 (m, 8F).

4-(3,5-Bis(((*S*)-3,7-dimethyloctyl)carbamoyl)phenyl)-*N*^{2},*N*^{6}-bis((*S*)-3,7-dimethyloctyl)pyridine-2,6-dicarboxamide ((*S*)-PhePyTA).

Compound **3** (0.25 g, 0.3 mmol) was dissolved in 9 mL dry THF in a 50 mL flask equipped with a magnetic stir bar and the solution was cooled with an ice bath. (*S*)-3,7-Dimethyloctan-1-amine (0.32 g, 2.0 mmol) and triethylamine (0.28 mL, 2.0 mmol) were diluted with 4 mL dry THF and added dropwise to the solution of compound **3** at 0 °C. The reaction mixture was heated up to 50 °C and the mixture was stirred for 16 h at 50 °C. After cooling to room temperature, the solvent was removed by rotary evaporation and the crude solid was dissolved in chloroform (50 mL). This solution was washed with 1 M NaOH, and brine. The organic phase was dried over $MgSO_4$ and filtered. The solvent was removed by rotary evaporation, and the resulting solid was purified by flash column chromatography (silica, mixture of MeOH and $CHCl_3$, gradient from 0 to 5vol% of MeOH). **(*S*)-PhePyTA** was isolated as a white solid (0.20 g, yield: 91%) after the product was dried under vacuum at 50 °C for at least 12 h. The material was stored in a desiccator loaded with $CaSO_4$. ^{1}H NMR (400 MHz, $CDCl_3$): *δ* 8.54 (s, 2H), 8.28 (t, 1H, *J* = 1.6 Hz), 8.18 (d, 2H, *J* = 1.6 Hz), 7.75 (t, 2H, *J* = 6.0), 6.49 (t, 2H, *J* = 5.6 Hz), 3.57–3.49 (m, 8H), 1.73–1.65 (m, 4H), 1.59–1.45 (m, 12H), 1.39–1.12 (m, 24H), 0.97 (q, 12H, *J* = 5.3 Hz), 0.86 (q, 24H, *J* = 3.1 Hz). ^{13}C NMR (100 MHz, $CDCl_3$): *δ* 165.76, 163.17, 149.93, 149.90, 137.80, 136.40, 128.16, 126.77, 122.62, 39.26, 38.64, 38.00, 37.20, 37.16, 36.82, 36.71, 30.99, 30.83, 27.96, 24.71, 24.64, 22.71, 22.70, 22.61, 19.61, 19.55. MALDI–TOF MS: calculated m/z for $C_{55}H_{93}N_5O_4$: 887.72, found: 888.75 ($[M + H]^+$), 910.74, ($[M + Na]^+$), 926.74 ($[M + K]^+$).

4-(3,5-Bis(((*R*)-3,7-dimethyloctyl)carbamoyl)phenyl)-*N*[2],*N*[6]-bis((*S*)-3,7-dimethyloctyl)pyridine-2,6-dicarboxamide ((*R*)-PhePyTA).

A procedure was applied similar to the procedure used for the synthesis of **(*S*)-PhePyTA** until after the flash column chromatography. Subsequently, the product was dissolved in minimum amount of CH_2Cl_2. This concentrated solution was precipitated in acetonitrile. The white precipitate was collected by vacuum filtration and subsequently drying under vacuum at 50 °C for at least 12 h to afford **(*R*)-PhePyTA** as a white solid (0.12 g, yield: 77%). The material was stored in a desiccator loaded with $CaSO_4$. ^{1}H NMR (400 MHz, $CDCl_3$): δ 8.54 (s, 2H), 8.29 (s, 1H), 8.19 (s, 2H), 7.78 (t, 2H, J = 6.0), 6.57 (s, 2H), 3.58-3.47 (m, 8H), 1.75–1.65 (m, 4H), 1.58–1.44 (m, 12H), 1.38-1.11 (m, 24H), 0.97 (q, 12H, J = 5.1 Hz), 0.86 (q, 24H, J = 3.1 Hz). ^{13}C NMR (100 MHz, $CDCl_3$): δ 165.88, 163.22, 149.92, 149.84, 137.68, 136.38, 128.18, 126.82, 122.57, 39.26, 38.66, 38.02, 37.21, 37.16, 36.80, 36.69, 31.00, 30.86, 27.95, 24.70, 24.65, 22.71, 22.70, 22.60, 19.58, 19.54. MALDI–TOF MS: calculated m/z for $C_{55}H_{93}N_5O_4$: 887.72, found: 888.82 ($[M + H]^+$), 910.82, ($[M + Na]^+$).

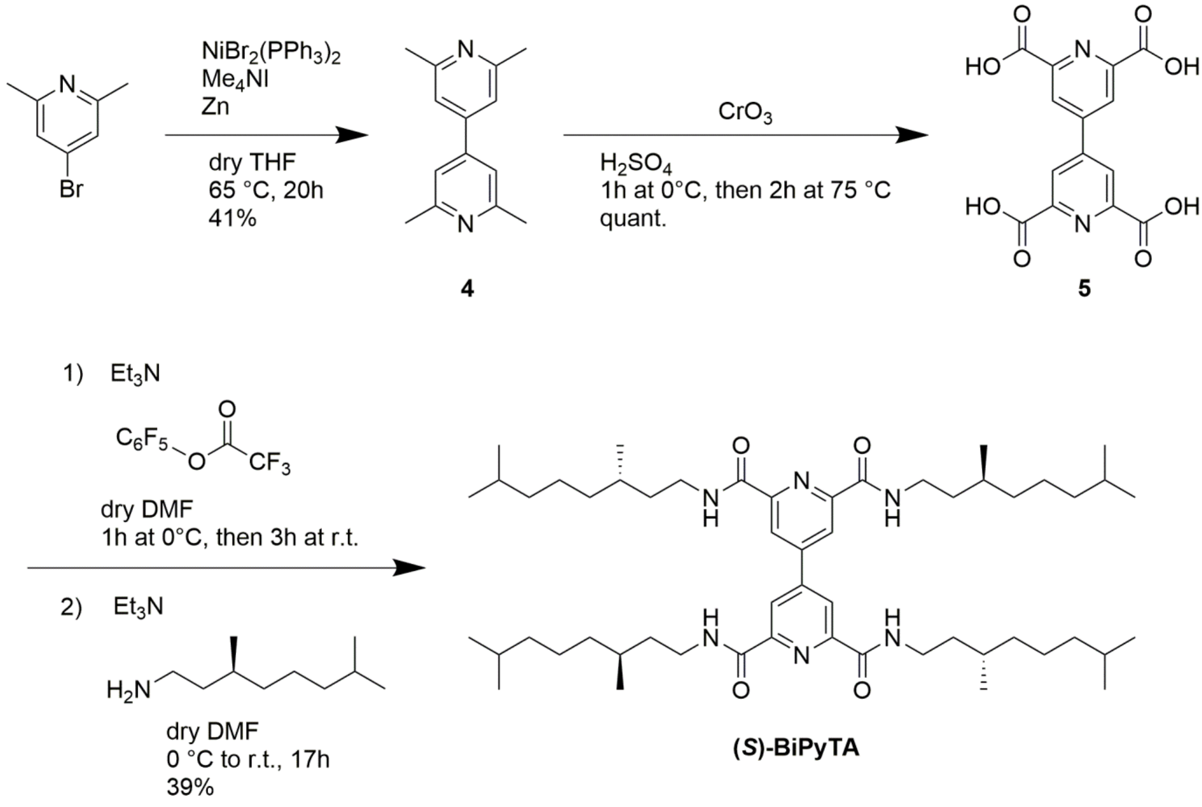

Scheme S2 Synthetic route to **(*S*)-BiPyTA**.

2,2′,6,6′-Tetramethyl-4,4′-bipyridine (4)

This procedure was taken from reference 8.

2,6-Dimethyl-4-bromopyridine (1.51 g, 8.1 mmol) was dissolved in dry THF (20 mL). $NiBr(PPh_3)_2$ (1.91 g, 2.4 mmol), Me_4NI (1.64 g, 8.1 mmol) and Zn dust (0.87 g, 13.3 mmol) were added. The reaction mixture was heated up to 65 °C and stirred for 20 h. Then the mixture was filtered over Celite and the filtrate was evaporated by rotary evaporator. The crude product was dissolved in $CHCl_3$ (50 mL) and washed with 1 M Na_2CO_3. Then 1M HCl was added to the organic layer and the aqueous phase was collected. A white precipitate was formed after the aqueous solution was basified to pH 10 by adding 1N NaOH. Subsequently, the suspension was extracted three times with an equal volume of $CHCl_3$. The organic phase was dried over $MgSO_4$ and filtered. The filtrate was concentrated by rotary

evaporator and the solid was dried under vacuum at room temperature for at least 2 h to afford compound **4** (0.70 g, yield: 41%). ^{1}H NMR (400 MHz, $CDCl_3$): δ 7.17 (s, 4H), 2.60 (s, 12H).

2,2′,6,6′-Tetracarboxy-4,4′-bipyridine (5)

This procedure was taken from reference 9.

Compound **4** (0.40 g, 1.9 mmol) was dissolved in concentrated sulfuric acid (15 mL). Chromium trioxide (2.81 g, 28.1 mmol) was added portionwise to the solution at 0 °C over 1 h. The reaction mixture was stirred at room temperature for 1 h and then at 75 °C for another 3 h. The mixture was poured into water (250 mL) and stirred at 0 °C for 30 min. The white precipitate was collected by centrifugation. The solid was dried under vacuum at 50 °C for at least 12 h to afford compound **5** (0.74 g, yield: quantitative). ^{1}H NMR (400 MHz, DMSO): δ 8.62 (s, 4H).

N^{2},$N^{2\prime}$,N^{6},$N^{6\prime}$-tetra((*S*)-3,7-dimethyloctyl)-[4,4′-bipyridine]-2,2′,6,6′-tetracarboxamide ((*S*)-BiPyTA)

Compound **5** (119.2 mg, 0.4 mmol) was dissolved in dry DMF (5 mL). Then triethylamine (0.5 mL, 3.6 mmol) was added. The solution was cooled to 0 °C using an ice bath. In a second round bottom flask, pentafluorophenyl trifluoroacetate (607.0 mg, 2.2 mmol) and triethylamine (0.05 mL, 0.4 mmol) were dissolved in dry DMF (3 mL). This solution was added dropwise to the mixture containing compound **5**, stirred at 0 °C for 1 h and then at room temperature for another 3 h. As a next, (*S*)-3,7-dimethyloctan-1-amine (452.0 mg, 2.9 mmol) and triethylamine (0.05 mL, 0.4 mmol) were diluted with 4 mL dry DMF and added to the solution of compound **5** dropwise at 0 °C. The reaction mixture was stirred at room temperature for 17 h. The mixture was diluted with EtOAc/hexane (1/1) and the organic layer was washed with 1M NaOH and brine. The organic phase was dried over $MgSO_4$, filtrated and evaporated *in vacuo*. The residue was purified by flash column chromatography (silica, mixture of MeOH and $CHCl_3$, gradient from 0 to 5vol% of MeOH). After recrystallization from CH_2Cl_2/diethylether (1/10), a white solid was obtained, that was isolated by centrifugation. The white solid was dried under vacuum at room temperature for at least 2 h to afford **(*S*)-BiPyTA** as a white solid (123.6 mg, yield: 39%). The material was stored in a desiccator loaded with $CaSO_4$. ^{1}H NMR (400 MHz, $CDCl_3$): δ 8.73 (s, 4H), 7.63 (t, 4H, *J* = 6.0 Hz), 3.60–3.54 (m, 8H), 1.75–1.68 (m, 4H), 1.57–1.48 (m, 12H), 1.38–1.13 (m, 24H), 0.99 (d, 12H, *J* = 6.4 Hz), 0.86 (d, 24H, *J* = 6.6 Hz). ^{13}C NMR (100 MHz, $CDCl_3$): δ 162.77, 150.33, 147.67, 122.75, 39.25, 38.02, 37.19, 36.81, 30.99, 27.96, 24.70, 22.70, 22.61, 19.62. MALDI–TOF MS: calculated m/z for $C_{54}H_{92}N_6O_4$: 888.72, found: 889.73 ($[M + H]^+$), 911.73, ($[M + Na]^+$).

POM images of **(*S*)-PhePyTA** and **(*S*)-BiPyTA**

(*S*)-PhePyTA

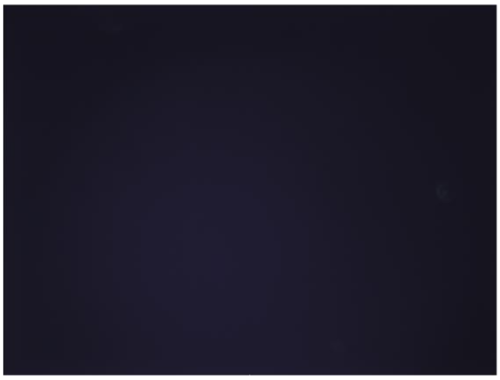

(*S*)-BiPyTA

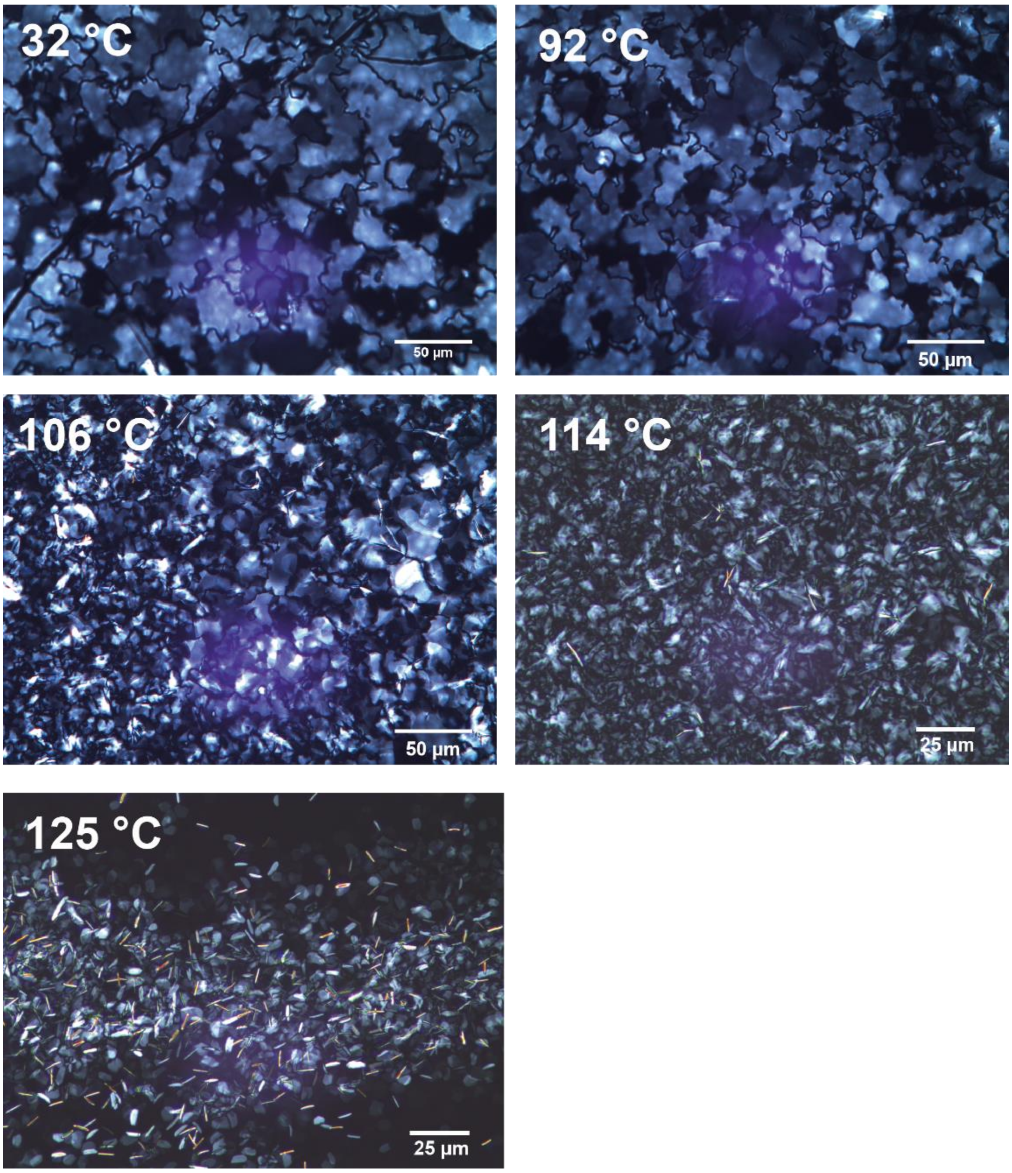

Figure S1 POM image of **(*S*)-PhePyTA** and **(*S*)-BiPyTA**. Materials were placed between two glass plates and the material was heated to the isotropic state.

DSC traces of **(*S*)-PhePyTA** and **(*S*)-BiPyTA**

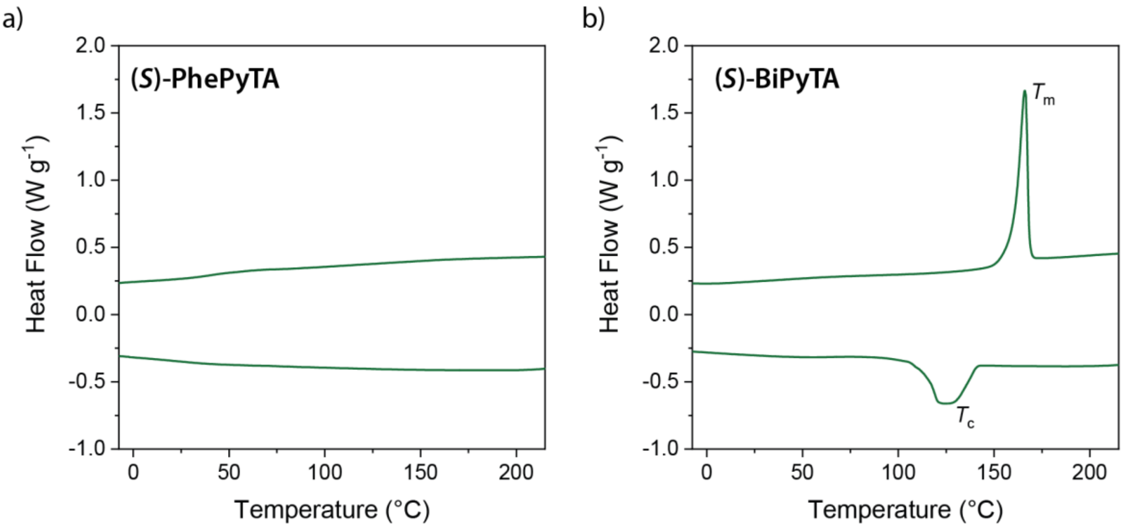

Figure S2 DSC results of a) **(*S*)-PhePyTA** and b) **(*S*)-BiPyTA**

Solid state SAXS results of **(*S*)-PhePyTA** and **(*S*)-BiPyTA**

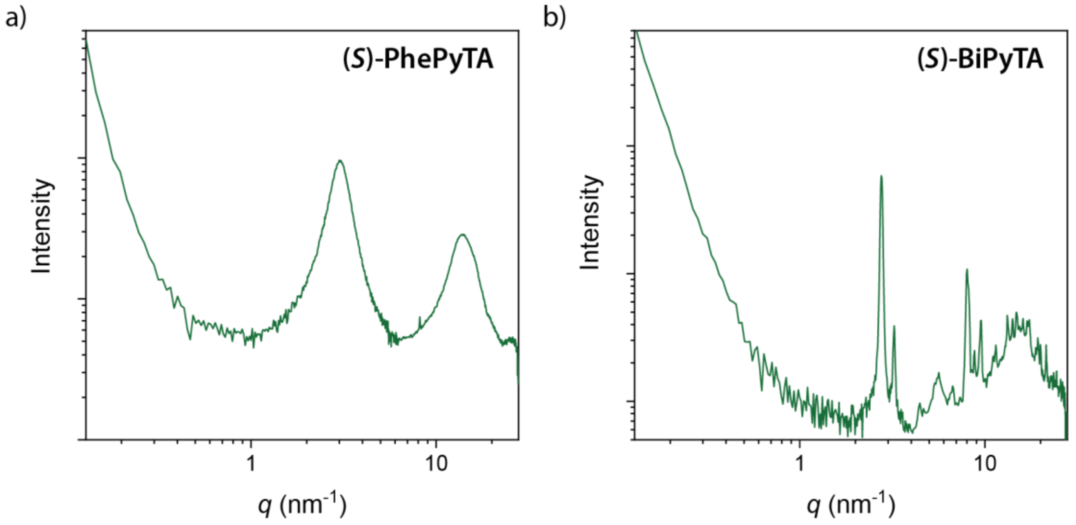

Figure S3 1D transmission scattering data of a) **(*S*)-PhePyTA** and b) **(*S*)-BiPyTA** measured as bulk powder in a capillary (1 mm diameter).

Bulk IR spectra of **(*S*)-BPTA**, **(*S*)-PhePyTA** and **(*S*)-BiPyTA**

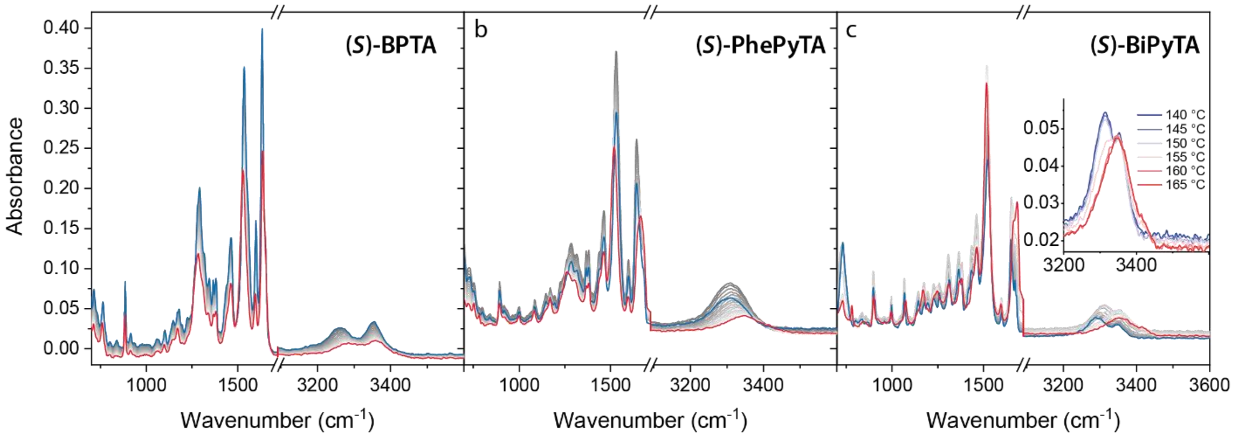

Figure S4 Variable-temperature IR spectra of **(*S*)-BPTA** (a), **(*S*)-PhePyTA** (b) and **(*S*)-BiPyTA** (c). The sample was heated from 30 °C (blue) to 205 °C (red) with a heating rate of 5 °C/min and full spectra recorded in intervals of 5 °C. The inset in c) shows a zoom in of the amide region.

Solution state SAXS measurements on **(*S*)-PhePyTA**

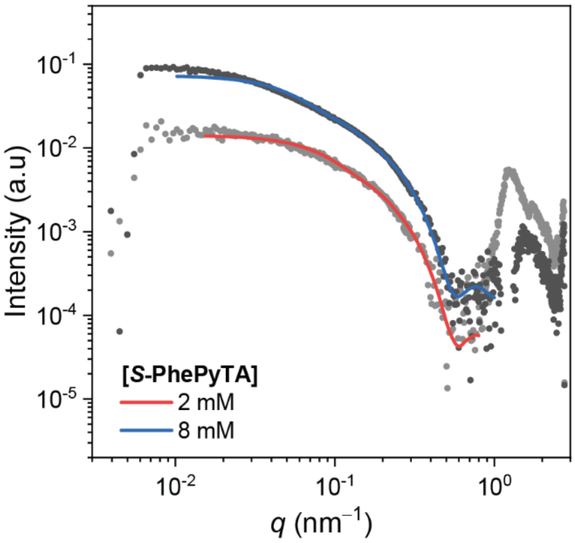

Figure S5 Scattering intensity as a function of the magnitude of the scattering vector measured for **(*S*)-PhePyTA** in MCH containing 32 ppm H_2O at 2 and 8 mM and 20 °C. A model of a rigid cylinder was fitted to the data to determine the radius and length. The lengths obtained from the fit are given in Table S1.

Table S1 Radius and length determined by fitting a model of a rigid cylinder to the data.

Concentration (mM)	Radius (nm)	Length (nm)
2	6.53±0.003	53.9±0.03
8	6.59±0.001	86.5±0.02

AFM micrographs of **(*S*)-PhePyTA**

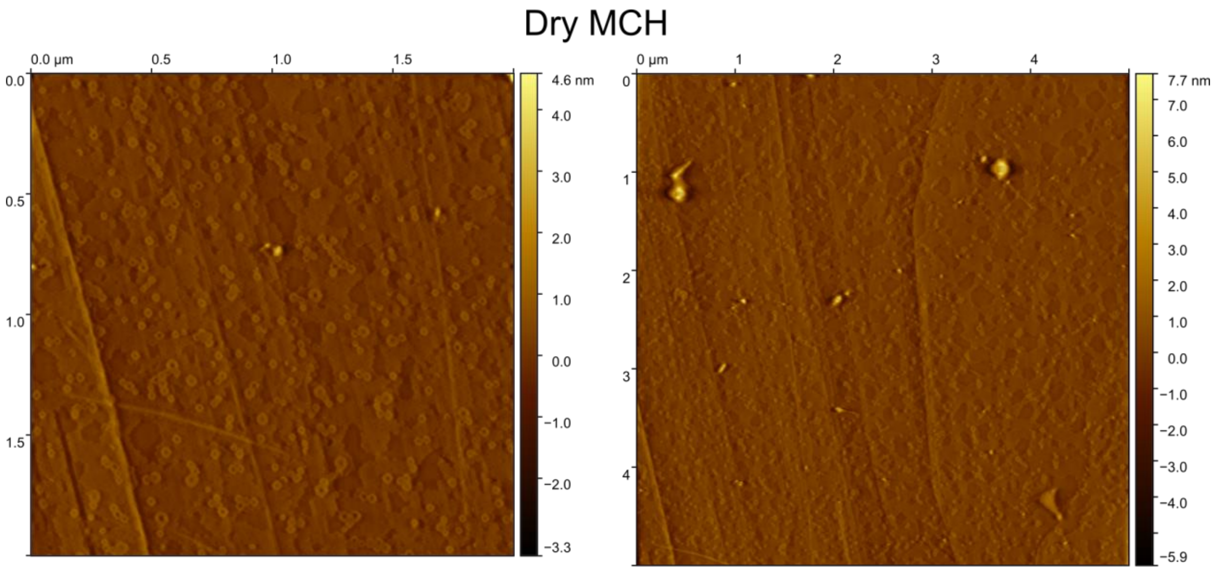

Figure S6 AFM micrographs of **(*S*)-PhePyTA** on HOPG surfaces dropcasted from 15 µM solutions in dry MCH containing 10 ppm H_2O. The toroidal structures are very likely surface artefacts, since the scattering pattern expected for toroidal structures is not observed in SAXS.[10]

VT-UV absorbance traces

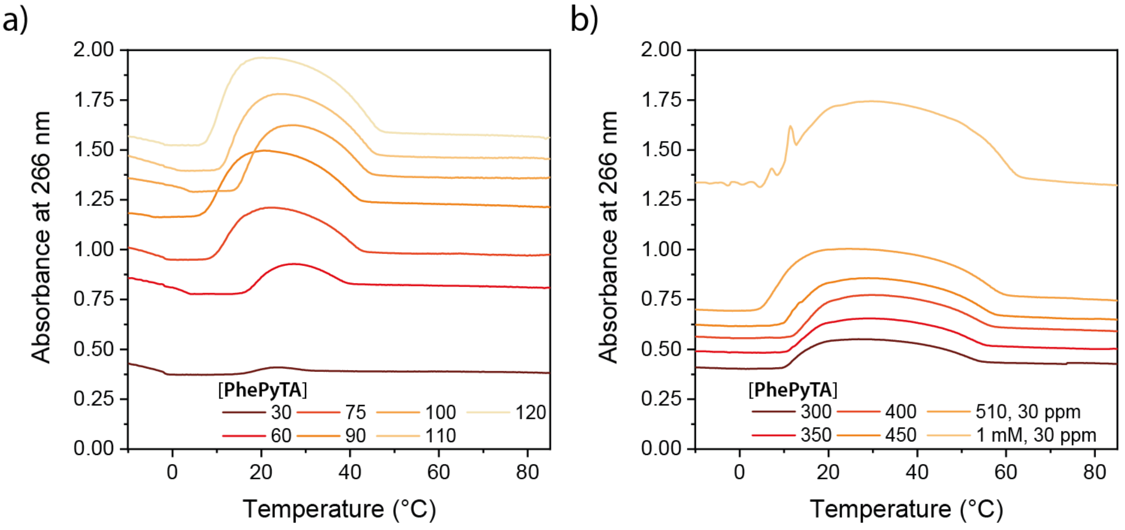

Figure S7 VT-UV traces of solutions in ambient MCH containing between 21 and 32 ppm H_2O with various concentrations of **(*S*)-PhePyTA** between a) 30 and 120 µM and b) 300 µM and 1 mM. The samples containing 510 µM and 1 mM **(*S*)-PhePyTA** contained 30 ppm H_2O.

Comparison of experimental IR and VCD spectra with calculated spectra of helically stacked geometry

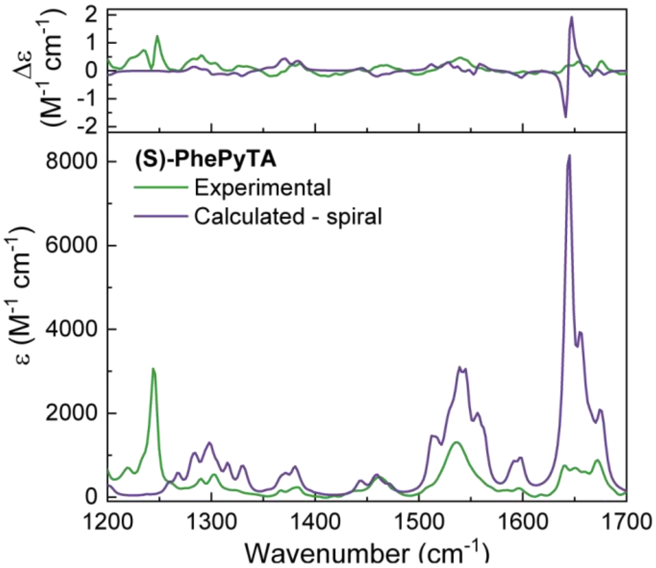

Figure S8 Experimental (green) and calculated (purple) VCD (top panel) and IR spectra (lower panel).

^{1}H and NOESY NMR of PhePyTA at 80 °C in d_{14}-MCH

a)

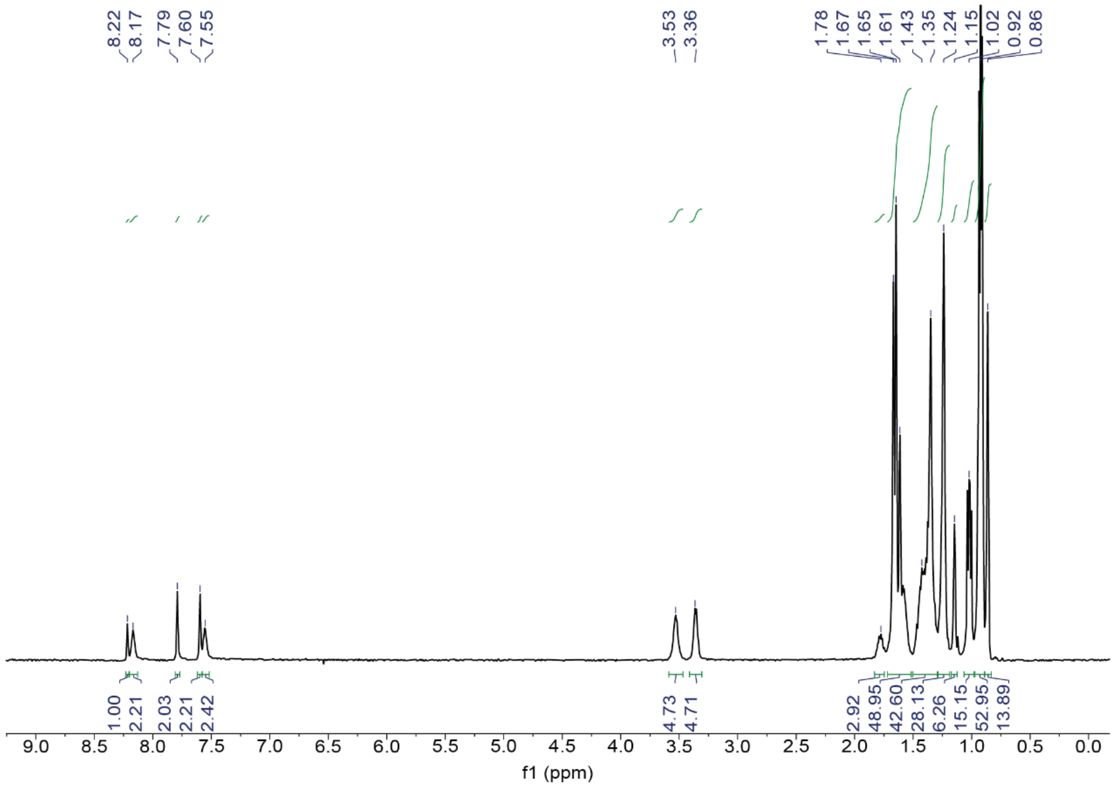

b)

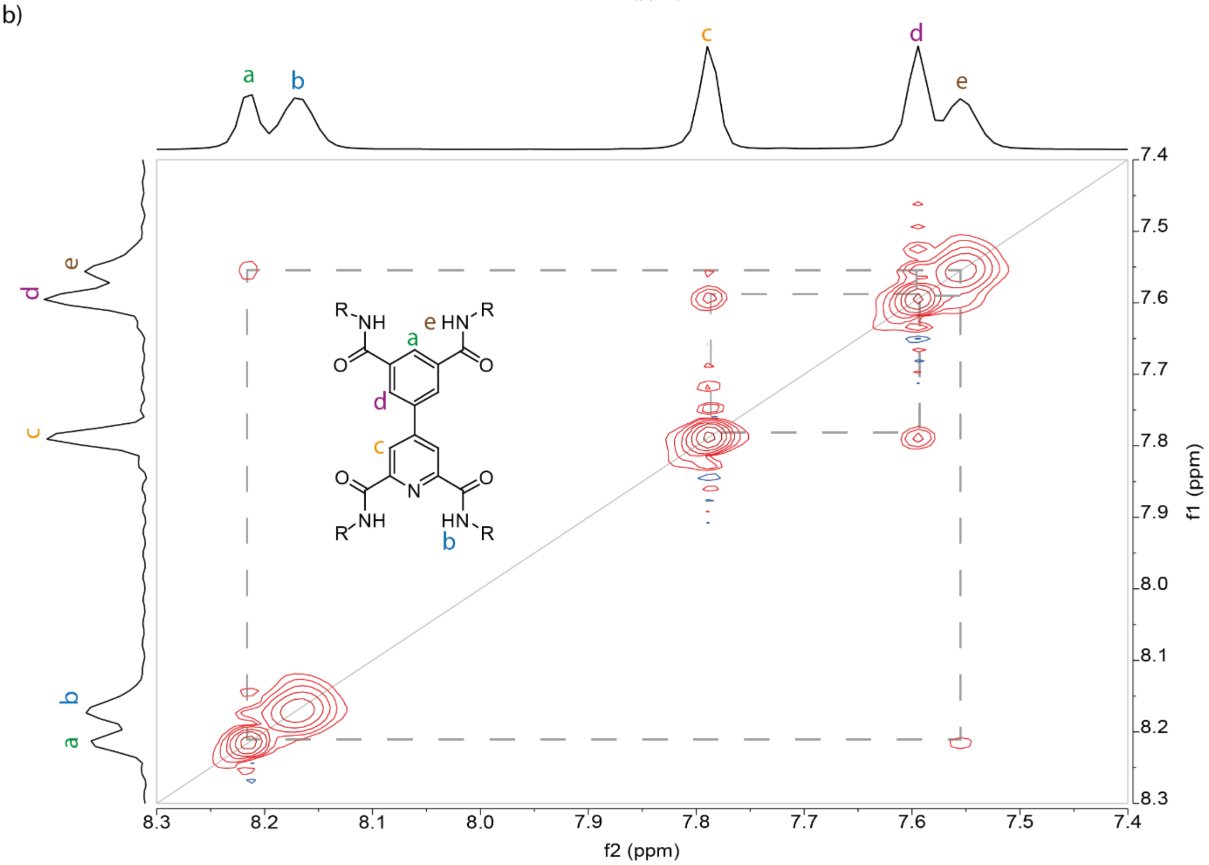

Figure S9 ^{1}H (a) and NOESY (b) NMR of a 5 mM solution of **(*S*)-PhePyTA** in d_{14}-MCH. The mixing time of the NOESY experiment was 250 ms. Both experiments were conducted at 80 °C.

VT-CD traces of **(*S*)-** and **(*R*)-PhePyTA**

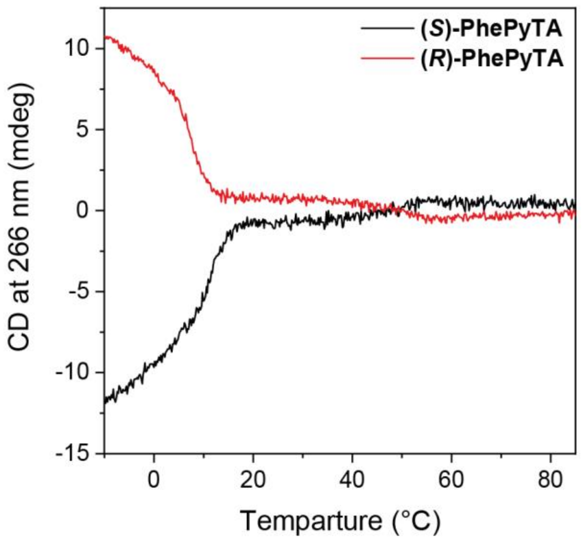

Figure S10 VT-CD signal at 266 nm of solutions containing 300 µM **(*S*)-PhePyTA** and **(*R*)-PhePyTA** in ambient MCH, containing 32 ppm water.

Van 't Hoff analysis of the **(*S*)-PhePyTA** homopolymerization into $\mathbf{A_{PhePyTA}}$

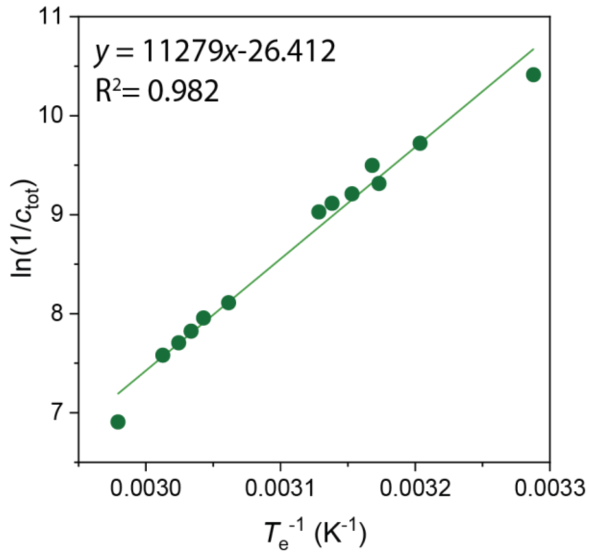

Figure S11 Van 't Hoff analysis of the homopolymerization of **(*S*)-PhePyTA** into $\mathbf{A_{PhePyTA}}$. The elongation temperatures at the various concentrations, indicated by the circles, are obtained from the data in Figure 3 in the main text. The line represent the linear fit through the data. From the fit, an enthalpy and entropy of −94 kJ/mol and −220 J/mol/K are obtained.

Temperature and angle-dependent scattering results for **(*S*)-PhePyTA**

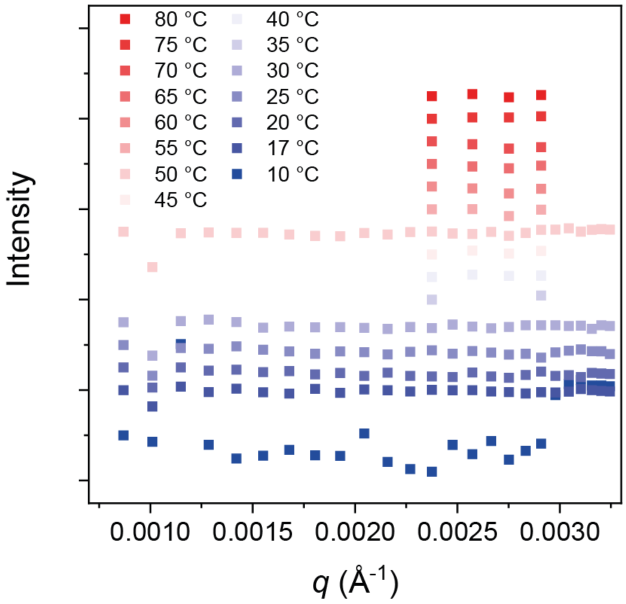

Figure S12 Angle-dependent SLS results for 2 mM **(*S*)-PhePyTA** solutions in MCH saturated with water. The traces for each temperature are vertically offset for clarity.

AFM micrograph of **(*S*)-BiPyTA**

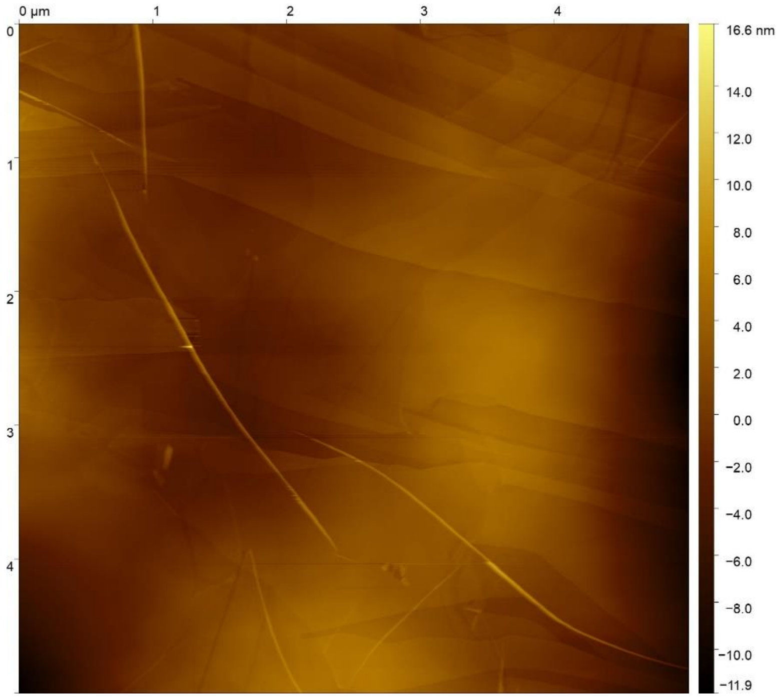

Figure S13 AFM micrograph of **(*S*)-BiPyTA** on HOPG surfaces dropcasted from a dry 15 µM MCH solution, containing less than 10 ppm H_2O.

Rate dependency of VT-CD results of **(*S*)-BiPyTA**

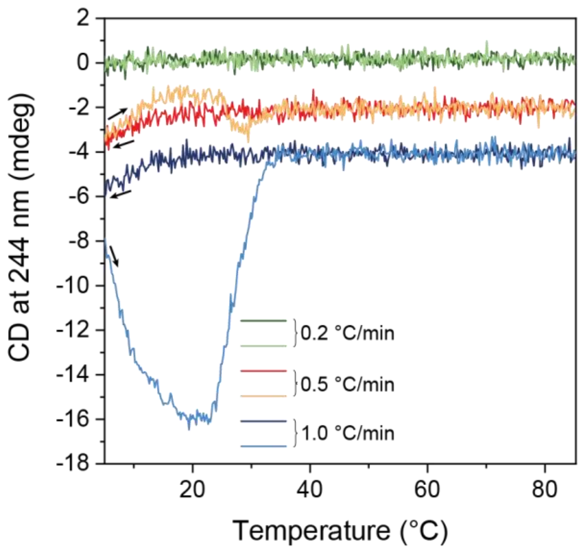

Figure S14 VT-CD intensity at 244 nm for samples containing 15 µM **(*S*)-BiPyTA** in ambient MCH containing 23 ppm water. The traces were collected at various cooling (dark shades) and heating (light shades) rates.

VT-CD and UV spectra of the copolymerization of **(*S*)-BPTA** and **(*S*)-PhePyTA**

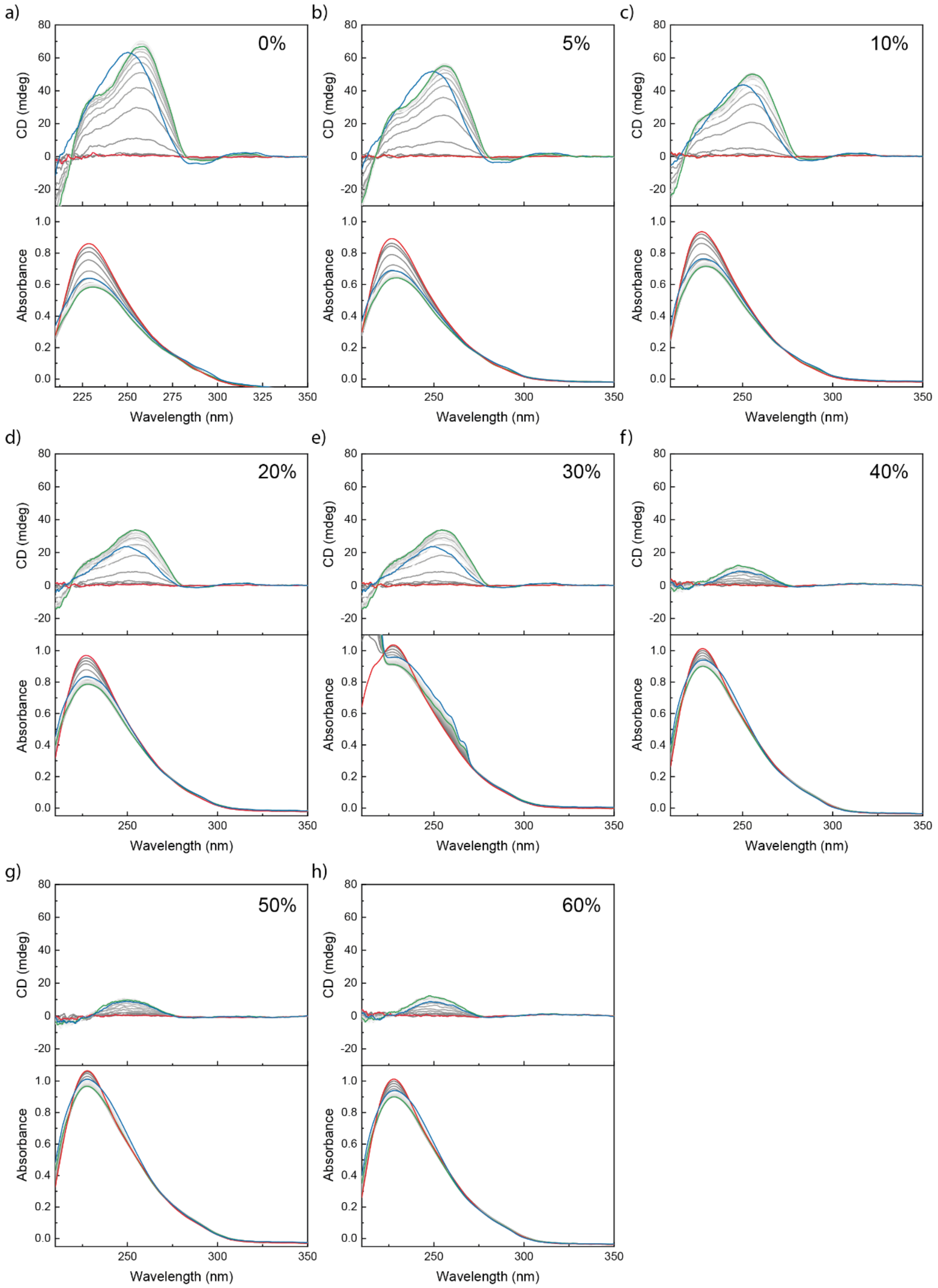

Figure S15 VT-CD (top panels) and UV (bottom panels) spectra of the copolymerizations of **(*S*)-BPTA** with **(*S*)-PhePyTA** with a total monomer concentration of 30 µM and (a) 0 mol% **(*S*)-PhePyTA**, (b) 5 mol% **(*S*)-PhePyTA**, (c) 10 mol% **(*S*)-PhePyTA**, (d) 20 mol% **(*S*)-PhePyTA**, (e) 30 mol% **(*S*)-PhePyTA**, (f) 40 mol% **(*S*)-PhePyTA**, (g) 50 mol% **(*S*)-PhePyTA** and (h) 60 mol% **(*S*)-PhePyTA** in MCH containing less than 10 ppm H_2O.

VT-CD of **(*S*)-BPTA** and **(*S*)-PhePyTA** copolymers upon heating and cooling

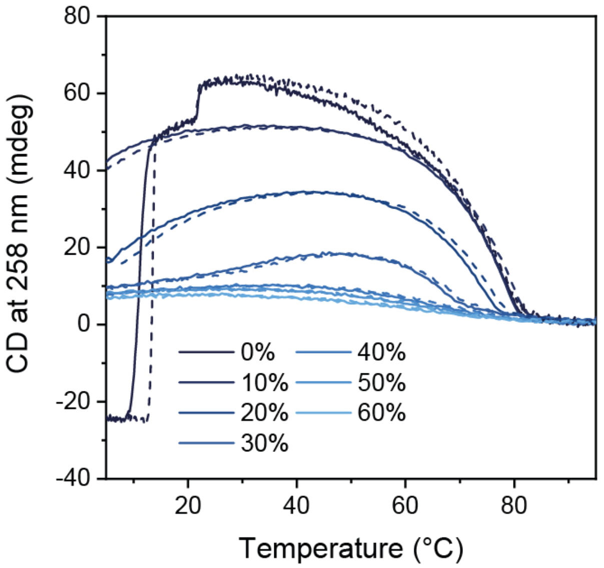

Figure S16 VT-CD traces of 30 μM samples containing various ratios of **(*S*)-BPTA** and **(*S*)-PhePyTA** upon cooling (solid lines) and heating (dashed lines). The samples were cooled and heated at a rate of 0.5 °C/min in MCH containing less than 10 ppm H_2O.

Correlations in the optimized fit parameters of the fit to the sequestration model

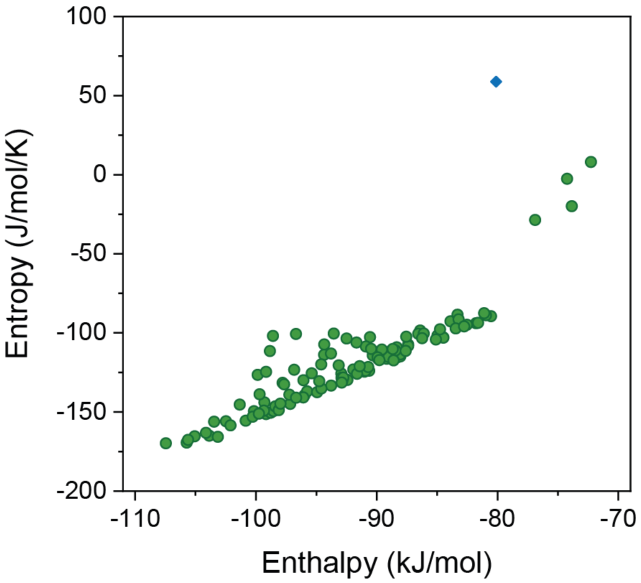

Figure S17 Entropy-enthalpy combinations of the sequestration of **(*S*)-BPTA** by **(*S*)-PhePyTA** as obtained from randomly sampled starting parameters in the fit of the mass-balance model to the experimental data (Figure 6b in the main text). The resulting fit of each entropy-enthalpy combination has a norm of the residual cost vector (see details below) less than 1% above the best fit, which is indicated by the solid blue diamond.

Results of fitting of VT-CD results **(*S*)-BPTA**:**(*S*)-PhePyTA** copolymerization over extended temperature range

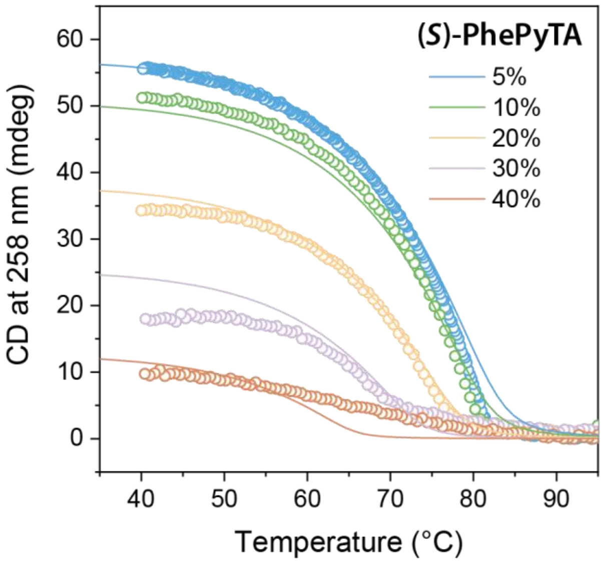

Figure S18 Result of the fitting of the VT-CD results of the copolymerization of 100 µM solutions containing various amounts of **(*S*)-BPTA** and **(*S*)-PhePyTA**. Details on the fitting routine can be found below.

Angle-dependent SLS results of mixtures of **(*S*)-BPTA** and **(*S*)-PhePyTA**

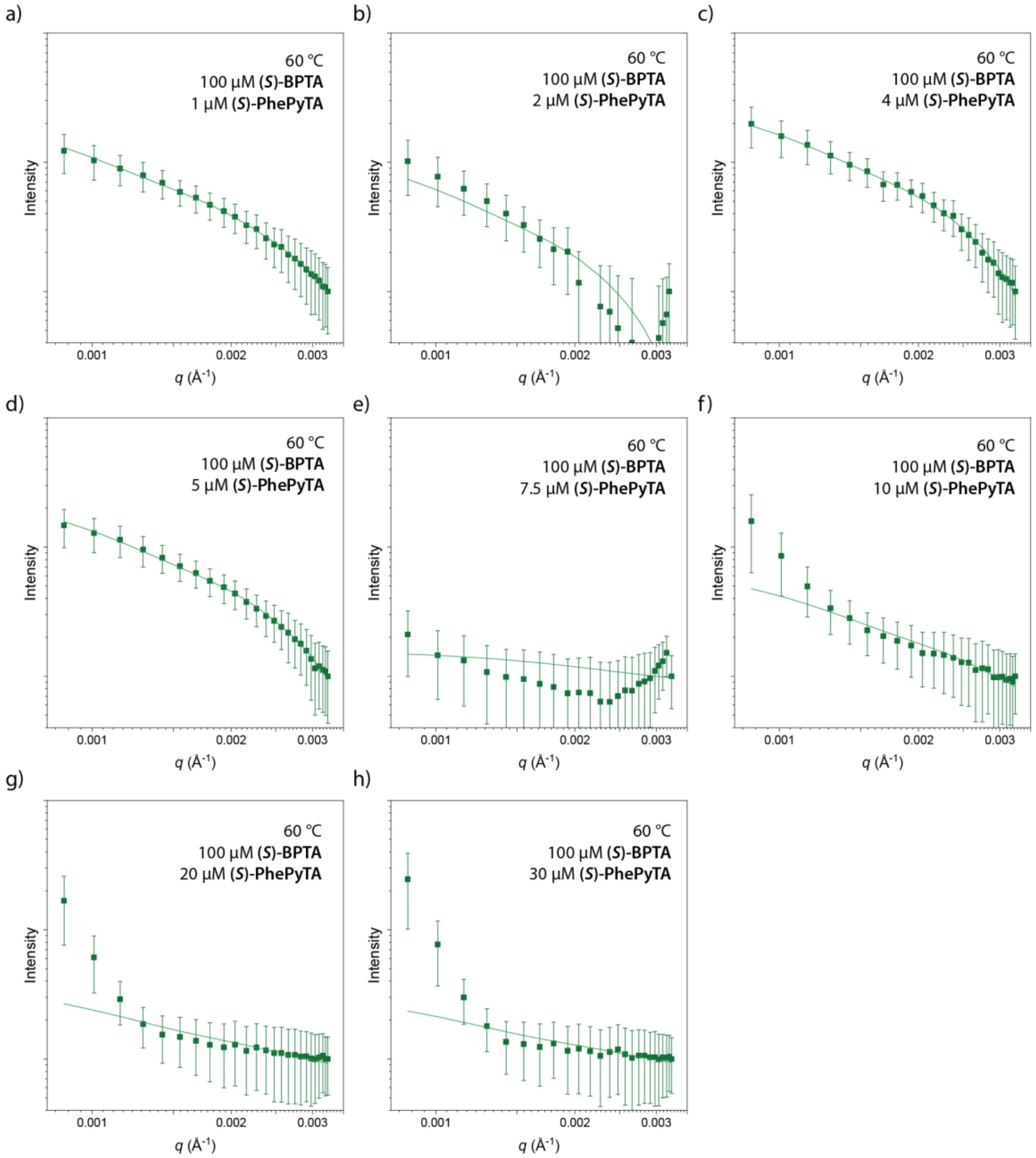

Figure S19 SLS results of solutions in dry MCH containing 100 μM **(*S*)-BPTA** and a) 1, b) 2, c) 4, d) 5, e) 7.5, f) 10, g) 20 and h) 30 μM **(*S*)-PhePyTA** at 60 °C. The squares indicate the data and the line indicates a fit to a cylindrical model.

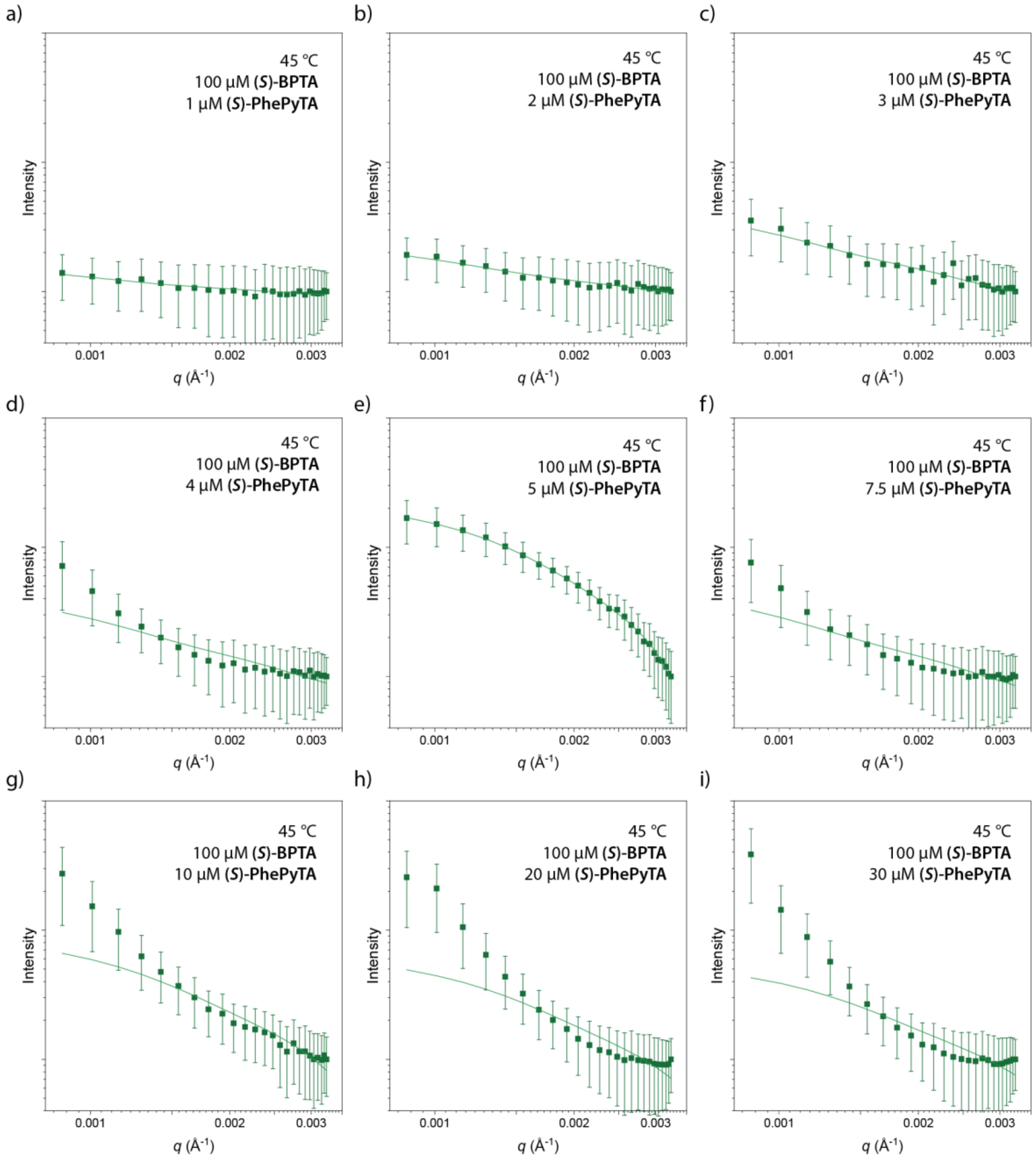

Figure S20 SLS results of solutions in dry MCH containing 100 μM ***(S)*-BPTA** and a) 1, b) 2, c) 3, d) 4, e) 5, f) 7.5, g) 10, h) 20, and i) 30 μM ***(S)*-PhePyTA** at 45 °C. The squares indicate the data and the line indicates a fit to a cylindrical model.

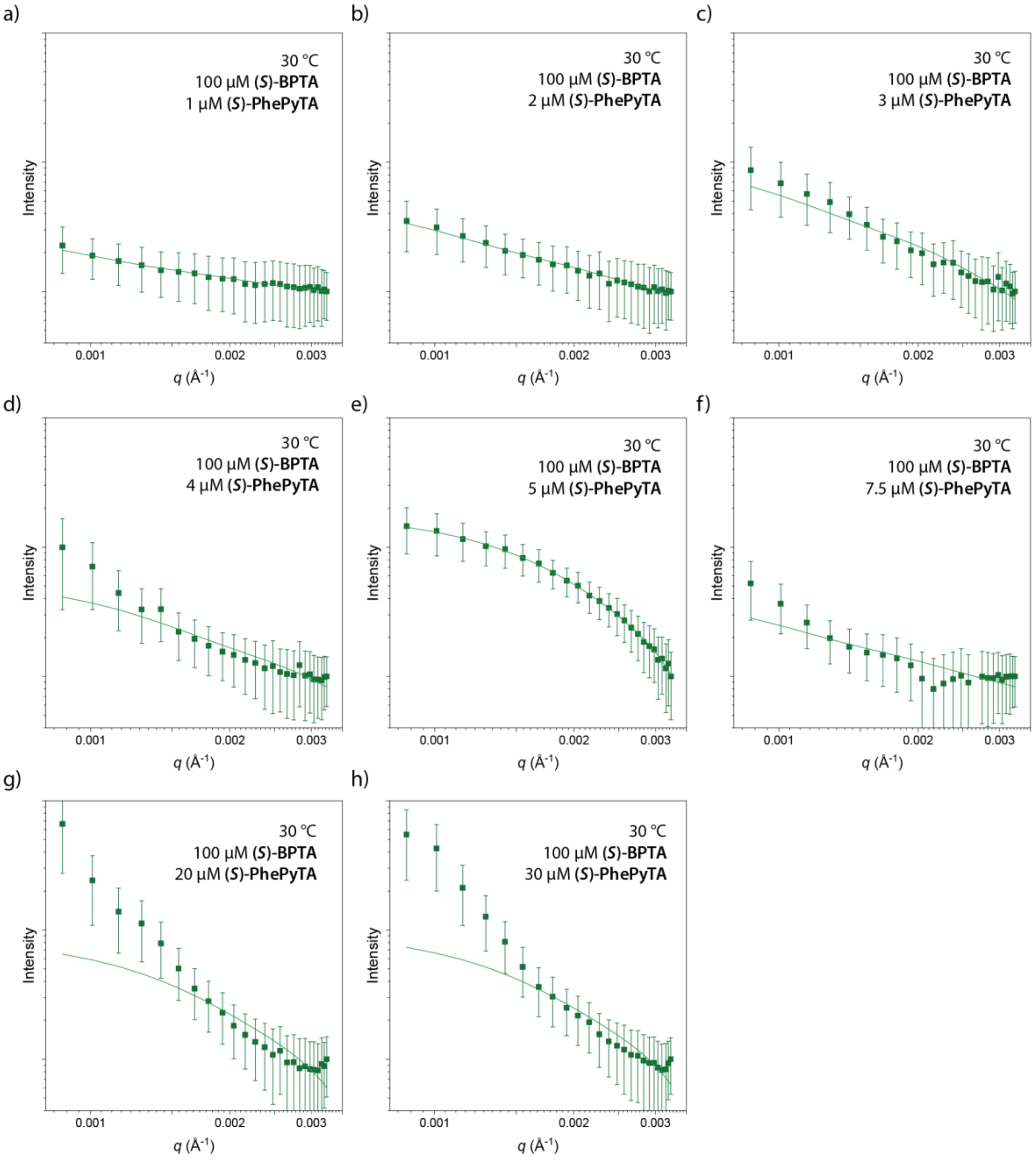

Figure S21 SLS results of solutions in dry MCH containing 100 µM **(*S*)-BPTA** and a) 1, b) 2, c) 3, d) 4, e) 5, f) 7.5, g) 20 and h) 30 µM **(*S*)-PhePyTA** at 30 °C. The squares indicate the data and the line indicates a fit to a cylindrical model.

Fitted parameters of the angle-dependent scattering of **(*S*)-PhePyTA**

Angle-dependent scattering data was fitted with SasView using a cylindrical model. The sld, sld_solvent and radius parameter were fixed at $1\cdot10^{-6}\cdot Å^{-1}$, $4\cdot10^{-6}\cdot Å^{-1}$ and 65.8 Å, respectively.

**(*S*)-PhePyTA**, 30 °C	1 mol%	2 mol%	3 mol%
Length (Å)	5771.1±19669	5253.6±9201.2	5240.1±6336.6
Background (cm^{-1})	-0.5841±0.31048	-1.1189±0.37909	-2.2284±0.6133
Scale	3.8393-05±2.0192-05	8.7441-05±2.7543-05	0.00020292±4.8702-05
q-range (nm^{-1})	0.00087 - 0.00324	0.00087 - 0.00324	0.00087 - 0.00324
χ^2	0.0055942	0.010938	0.092651
	4 mol%	5 mol%	7.5 mol%
Length (Å)	4013.8±4748.6	3078.6±784.09	4455.6±8114.3
Background (cm^{-1})	-1.6168±0.59338	-3.1371±0.95135	-0.27988±0.40598
Scale	0.00013272±4.5631-05	0.00065782±7.6614e-05	6.8739-05±2.8113-05
q-range (nm^{-1})	0.00087 - 0.00324	0.00087 - 0.00324	0.00087 - 0.00324
χ^2	0.1447	0.0078642	2.8258
	10 mol%	20 mol%	30 mol%
Length (Å)	3078.6±784.09	3476.6±3051.6	3468.2±2769.8
Background (cm^{-1})	-3.1371±0.95135	-2.5536±0.87547	-3.0464±0.88271
Scale	0.00065782±7.6614-05	0.0002644±6.5256-05	0.00030018±6.6645-05
q-range (nm^{-1})	0.00087 - 0.00324	0.00087 - 0.00324	0.00087 - 0.00324
χ^2	0.0078642	0.51941	0.56078

**(*S*)-PhePyTA**, 45 °C	1 mol%	2 mol%	3 mol%
Length (Å)	5285.9±34941	4534.7±9718.9	5060.9±10717
Background (cm^{-1})	-0.33748±0.2781	-0.55482±0.3459	-0.81795±0.41789
Scale	1.4568-05±1.7361-05	3.4088-05±2.2928-05	7.554-05±2.9914-05
q-range (nm^{-1})	0.00087 - 0.00324	0.00087 - 0.00324	0.00087 - 0.00324
χ^2	0.0070643	0.0098941	0.033827
	4 mol%	5 mol%	7.5 mol%
Length (Å)	4680.9±8651.3	3593.7±1160.7	5014.3±9911.9
Background (cm^{-1})	-1.1298±0.43992	-3.6663±1.0195	-1.0731±0.46477
Scale	8.3813-05±3.1154-05	0.00069749±8.4909-05	8.781-05±3.3343-05
q-range (nm^{-1})	0.00087 - 0.00324	0.00087 - 0.00324	0.00087 - 0.00324
χ^2	0.14893	0.0083916	0.14802
	10 mol%	20 mol%	30 mol%
Length (Å)	3831.6±3273.6	3660.1±3743.8	3701.2±4304.4
Background (cm^{-1})	-1.4407±0.80977	-1.6372±0.69168	-1.2644±0.6363
Scale	0.00024072±6.4429e-05	0.00018087±5.2486-05	0.00015037±4.7646-05
q-range (nm^{-1})	0.00087 - 0.00324	0.00087 - 0.00324	0.00087 - 0.00324
χ^2	0.24399	0.40653	0.41876

**(*S*)-PhePyTA**, 60 °C	1 mol%	2 mol%	4 mol%
Length (Å)	5793.9±4125.6	5462.7±6167	5128.3±2839.7
Background (cm^{-1})	-1.7569±0.76179	0.22678±0.74995	-3.2234±0.90323
Scale	0.00042513±6.0274-05	0.0028263±0.00062688	0.00066279±7.2311-05
q-range (nm^{-1})	0.00087 - 0.00324	0.00087 - 0.0032	0.00087 - 0.00324
χ^2	0.0070103	0.27099	0.025939

	5 mol%	7.5 mol%	10 mol%
Length (Å)	5201±3066.4	4591.5±6088.7	4591.5±6088.7
Background (cm^{-1})	-2.3422±0.77862	-0.57018±0.56651	-0.57018±0.56651
Scale	0.00053507±6.148-05	0.00014761±4.1733-05	0.00014761±4.1733-05
q-range (nm^{-1})	0.00087 - 0.00324	0.00087 - 0.00324	0.00087 - 0.00324
χ^2	0.012838	0.15523	0.15523

	20 mol%	30 mol%
Length (Å)	4935.6±12545	4828.2±15027
Background (cm^{-1})	-0.24442±0.43094	-0.1818±0.43617
Scale	6.3925-05±2.719-05	5.2092-05±2.7469-05
q-range (nm^{-1})	0.00087 - 0.00324	0.00087 - 0.00324
χ^2	0.23496	0.26593

VT-CD and UV spectra of the copolymerization of **(*S*)-BPTA** and **(*S*)-BiPyTA**

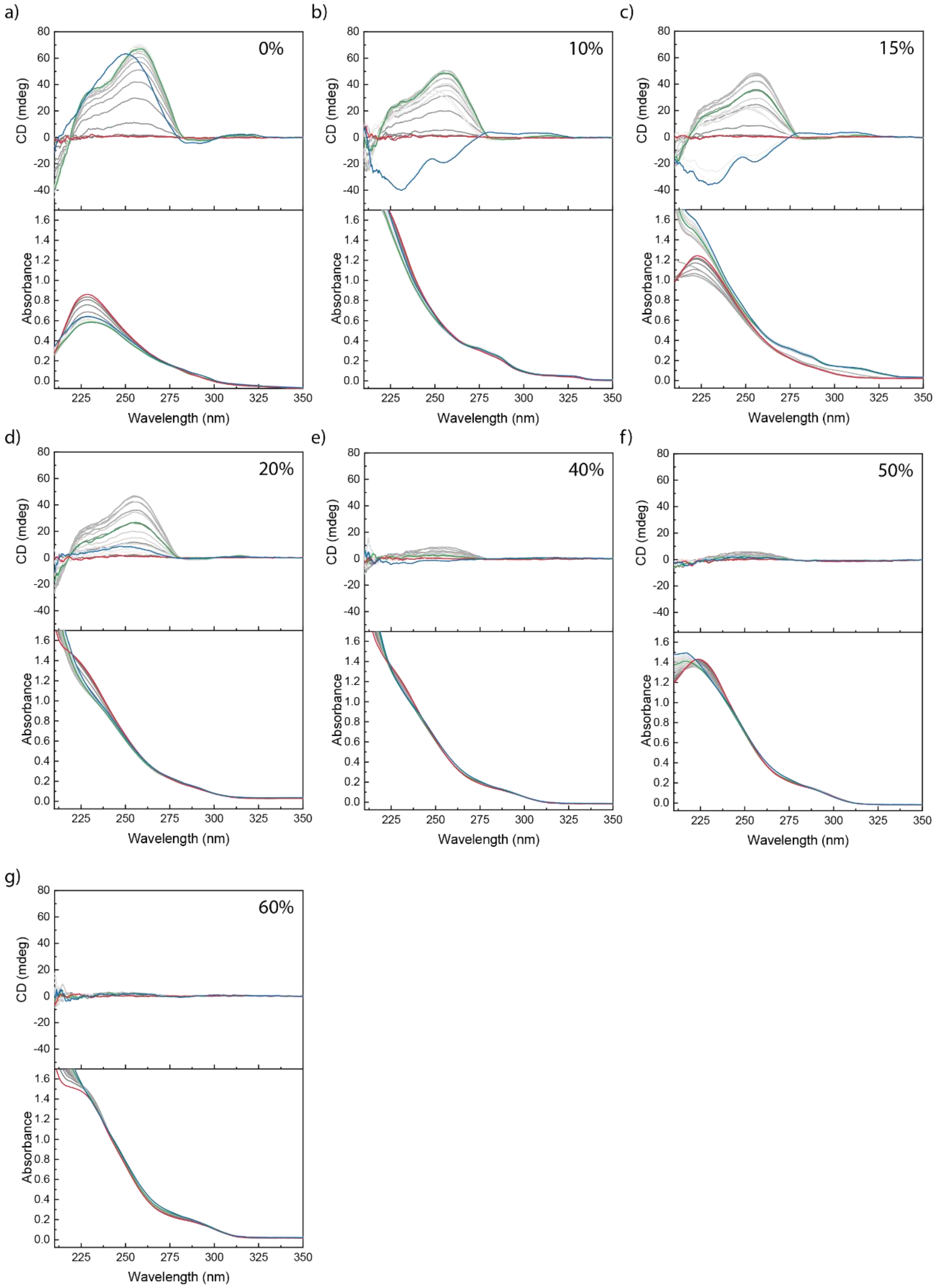

Figure S22 VT-CD (top panels) and UV (bottom panels) spectra of the copolymerizations of **(*S*)-BPTA** with **(*S*)-BiPyTA** with a total monomer concentration of 30 µM and (a) 0 mol% **(*S*)-BiPyTA**, (b) 10 mol% **(*S*)-BiPyTA**, (c) 15 mol% **(*S*)-BiPyTA**, (d) 20 mol% **(*S*)-BiPyTA**, (e) 40 mol% **(*S*)-BiPyTA**, (f) 50 mol% **(*S*)-BiPyTA** and (g) 60 mol% **(*S*)-BiPyTA** in MCH containing less than 10 ppm H_2O. The high absorbance in the sample containing 5 mol% **(*S*)-BiPyTA** is presumably caused by minute precipitates.

VT-CD of **(*S*)-BPTA** and **(*S*)-BiPyTA** copolymers upon heating and cooling

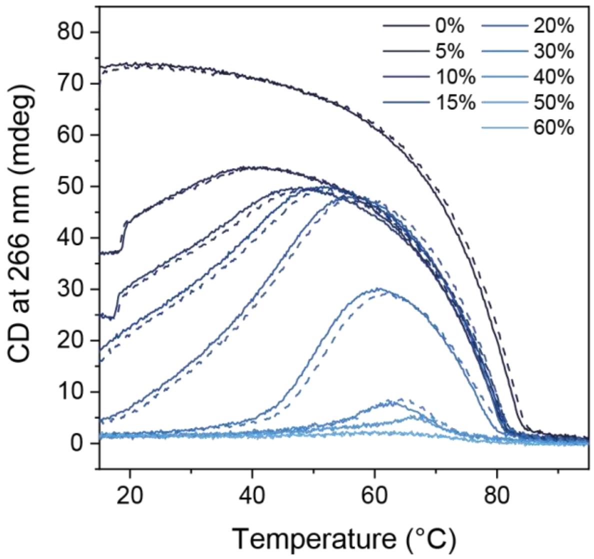

Figure S23 VT-CD traces of 30 µM samples containing various ratios of **(*S*)-BPTA** and **(*S*)-BiPyTA** upon cooling (solid lines) and heating (dashed lines). The samples were cooled and heated at a rate of 0.5 °C/min.

VT-CD of **(*S*)-BPTA** and 4,4'-bipyridine mixtures

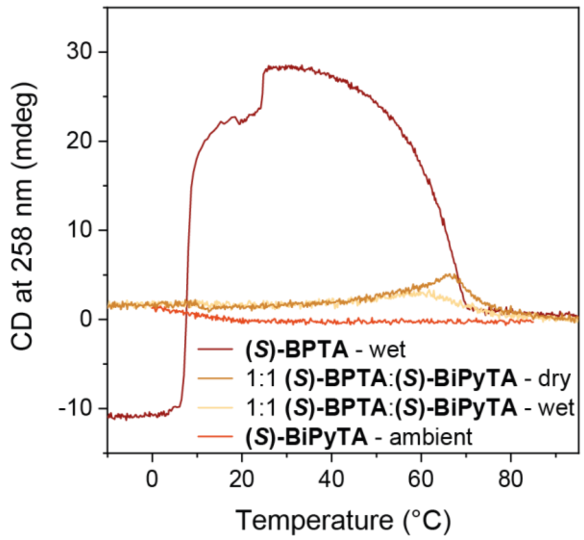

Figure S24 VT-CD traces of wet and dry MCH solutions, containing 60, 23 and less than 10 ppm water, respectively, and 15 µM **(*S*)-BPTA** and/or **(*S*)-BiPyTA**.

Angle-dependent SLS results of mixtures of **(*S*)-BPTA** and **(*S*)-BiPyTA**

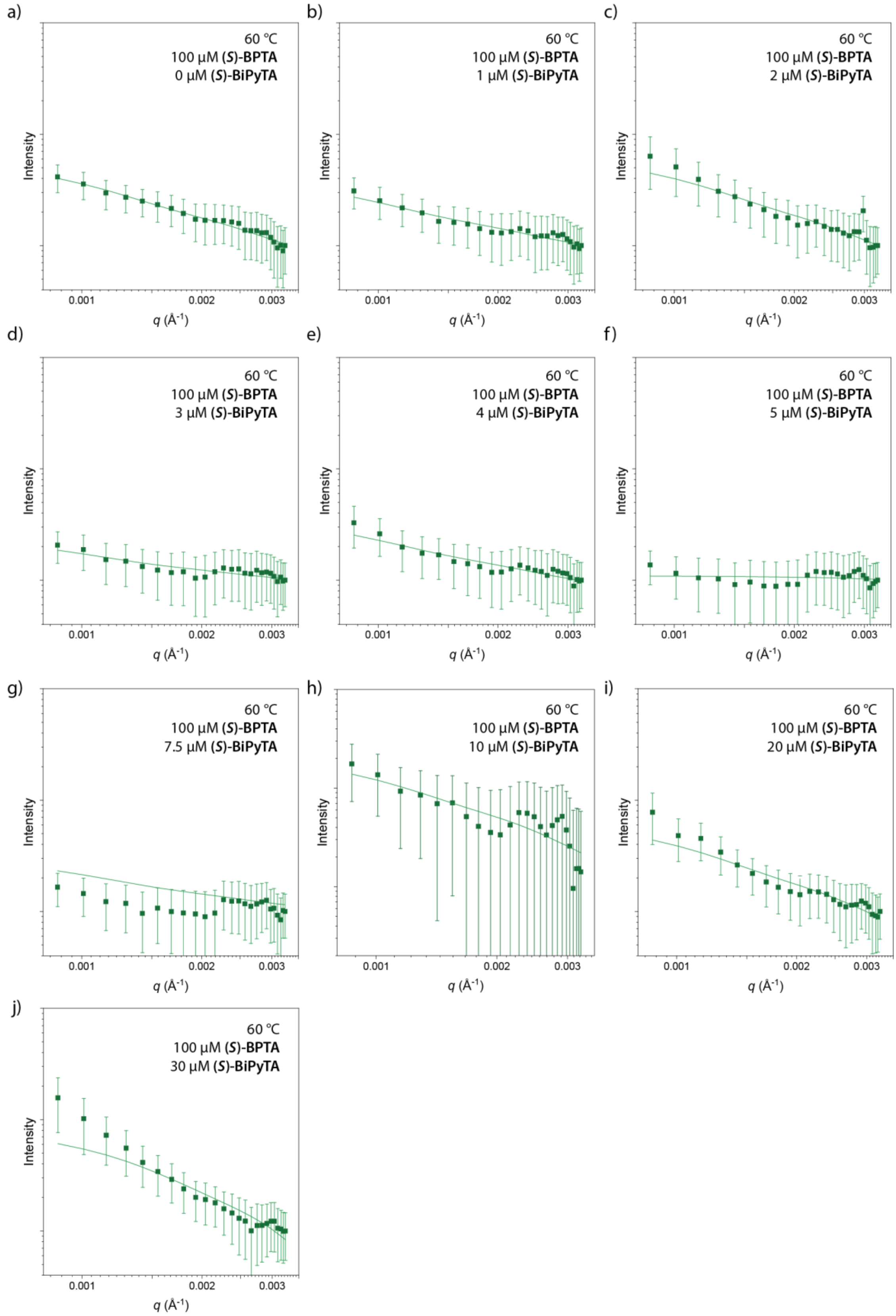

Figure S25 SLS results of solutions in dry MCH containing 100 µM **(*S*)-BPTA** and a) 0, b) 1, c) 2, d) 3, e) 4, f) 5, g) 7.5, h) 10, i) 20 and j) 30 µM **(*S*)-BiPyTA** at 60 °C. The squares indicate the data and the line indicates a fit to a cylindrical model.

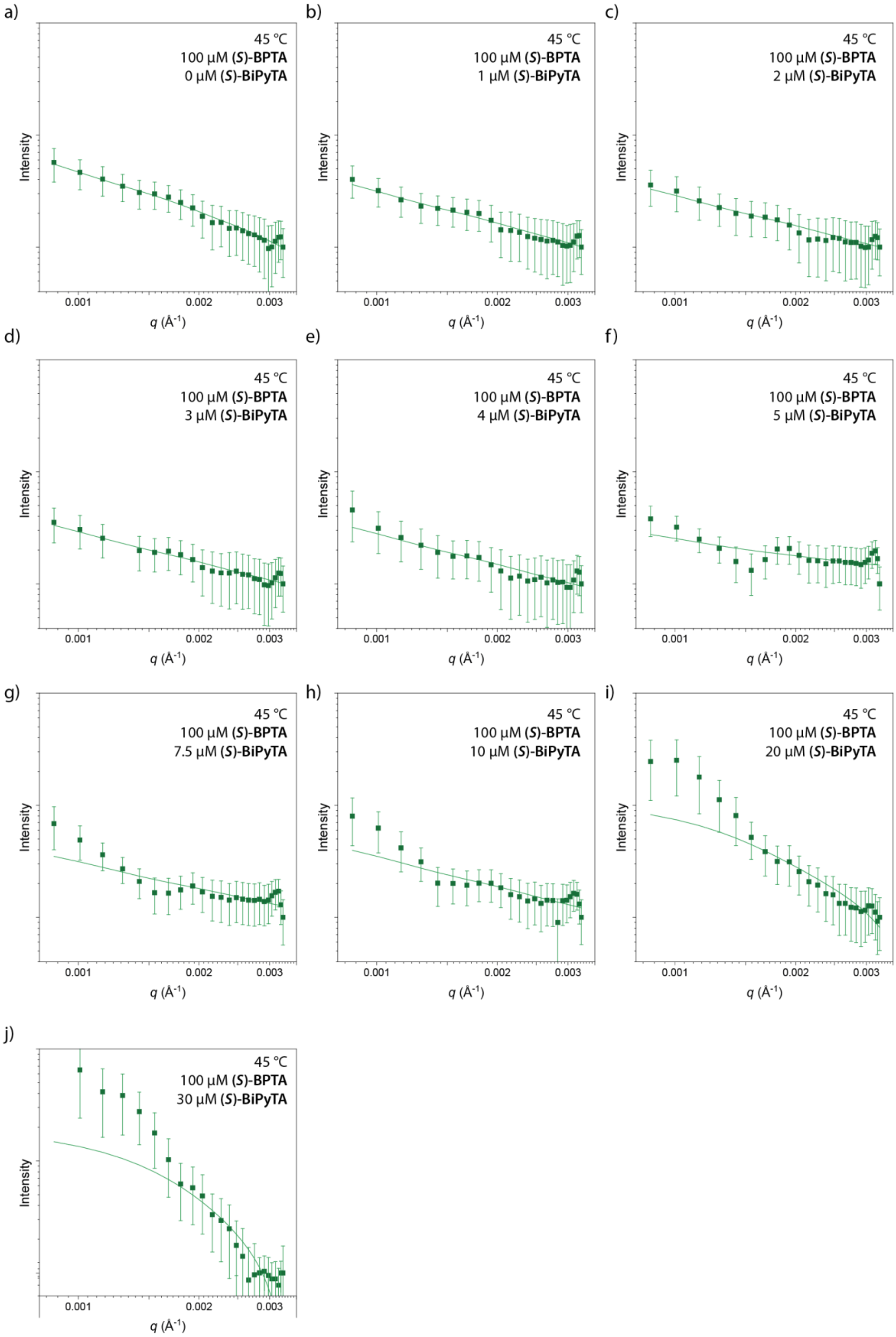

Figure S26 SLS results of solutions in dry MCH containing 100 µM **(*S*)-BPTA** and a) 0, b) 1, c) 2, d) 3, e) 4, f) 5, g) 7.5, h) 10, i) 20 and j) 30 µM **(*S*)-BiPyTA** at 45 °C. The squares indicate the data and the line indicates a fit to a cylindrical model.

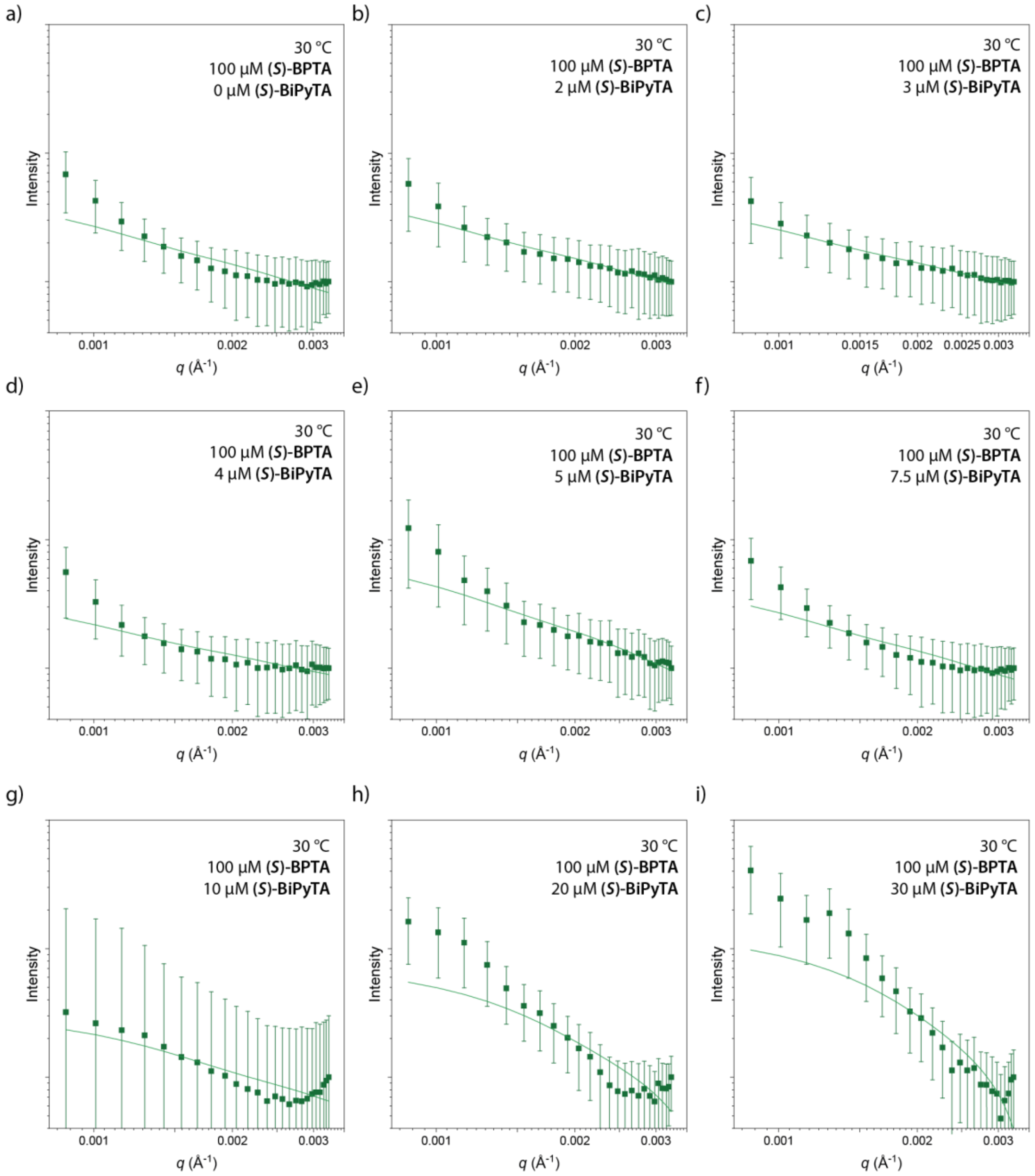

Figure S27 SLS results of solutions in dry MCH containing 100 µM **(*S*)-BPTA** and a) 0, b) 2, c) 3, d) 4, e) 5, f) 7.5, g) 10, h) 20 and i) 30 µM **(*S*)-BiPyTA** at 30 °C. The squares indicate the data and the line indicates a fit to a cylindrical model.

Fitted parameters of the angle-dependent scattering of **(*S*)-BiPyTA**

Angle-dependent scattering data was fitted with SasView using a cylindrical model. The sld, sld_solvent and radius parameter were fixed at $1\cdot10^{-6}\cdot\text{Å}^{-1}$, $4\cdot10^{-6}\cdot\text{Å}^{-1}$ and 65.8 Å, respectively.

**(*S*)-BiPyTA**, 30 °C	0 mol%	2 mol%	3 mol%
Length (Å)	5531.7±11690	5217.1±11280	5186.3±12152
Background (cm^{-1})	0.29582±0.59208	-0.88682±0.44802	-0.81264±0.40345
Scale	0.00010865±4.5456-05	8.2079-05±3.1947-05	6.875-05±2.8309-05
q-range (nm^{-1})	0.00073 - 0.00324	0.00087 - 0.00324	0.00087 - 0.00324
χ^2	0.22338	0.051661	0.030072
	4 mol%	5 mol%	7.5 mol%
Length (Å)	5215.7±14373	4802.2±7591	5010.5±9501.3
Background (cm^{-1})	-1.0583±0.39253	-1.0751±0.65427	-1.5666±0.41347
Scale	5.5582-05±2.6714e-05	0.00014571±5.0813-05	8.1062-05±2.8904-05
q-range (nm^{-1})	0.00087 - 0.00324	0.00087 - 0.00324	0.00087 - 0.00324
χ^2	0.10485	0.1022	0.16146
	10 mol%	20 mol%	30 mol%
Length (Å)	3763.3±4429	321.56±12737	3389±2652.5
Background (cm^{-1})	-3.6904±0.41801	-83.087±5080.8	-7.6149±1.3042
Scale	7.1207-05±3.0388-05	0.021166±2.1285	0.00042792±0.00010441
q-range (nm^{-1})	0.00087 - 0.00324	0.00087 - 0.00324	0.00087 - 0.00324
χ^2	0.15518	0.88884	0.51528

**(*S*)-BiPyTA**, 45 °C	0 mol%	1 mol%	2 mol%
Length (Å)	6538.3±8423.4	5978.1±10066	5592.7±8910.1
Background (cm^{-1})	-0.47069±0.45969	-0.40152±0.37734	-1.0574±0.63017
Scale	0.00015832±3.3626-05	9.1861-05±2.5972-05	0.00015887±4.9921-05
q-range (nm^{-1})	0.00087 - 0.00324	0.00087 - 0.00324	0.00087 - 0.00324
χ^2	0.040385	0.051584	0.095037
	3 mol%	4 mol%	5 mol%
Length (Å)	5735.7±11077	5587.9±12132	5352.7±14558
Background (cm^{-1})	-0.20975±0.39739	-0.37971±0.43048	-0.39093±0.32353
Scale	8.2057-05±2.8373-05	7.9739-05±3.1049-05	4.6402-05±2.0489-05
q-range (nm^{-1})	0.00087 - 0.00324	0.00087 - 0.00324	0.00087 - 0.00324
χ^2	0.044833	0.090053	0.36492

	7.5 mol%	10 mol%	20 mol%
Length (Å)	5666±12780	5299.8±9950.1	3550.8±2477.5
Background (cm^{-1})	-0.52846±0.31274	-1.0138±0.4684	-3.4604±0.88898
Scale	5.6592-05±1.998-05	9.8677-05±3.546-05	0.0003266±7.0759-05
q-range (nm^{-1})	0.00087 - 0.00324	0.00087 - 0.00324	0.00087 - 0.00324
χ^2	0.15468	0.30153	0.38901

	30 mol%
Length (Å)	3201.5±2649.3
Background (cm^{-1})	-7.1664±2.3102
Scale	0.00070693±0.00015951
q-range (nm^{-1})	0.00087 - 0.00324
χ^2	0.63705

**(*S*)-BiPyTA**, 60 °C	0 mol%	1 mol%	2 mol%
Length (Å)	4555.5±4218.9	5282.8±11229	4453.3±5782.5
Background (cm^{-1})	-0.2738±0.40959	-0.42754±0.33693	-1.0773±0.56591
Scale	0.00011235±2.8565-05	6.1173-05±2.2428-05	0.00013216±4.3237-05
q-range (nm^{-1})	0.00087 - 0.00324	0.00087 - 0.00324	0.00087 - 0.00324
χ^2	0.025882	0.035799	0.1302

	3 mol%	4 mol%	5 mol%
Length (Å)	5096.6±16649	5149.4±12893	10.739±
Background (cm^{-1})	-0.40918±0.30955	-0.46642±0.36594	-9.8206±
Scale	3.0068-05±2.0372-05	5.6026-05±2.4597-05	0.076155±
q-range (nm^{-1})	0.00087 - 0.00324	0.00087 - 0.00324	0.00087 - 0.00324
χ^2	0.04054	0.051064	0.067169

	7.5 mol%	10 mol%	20 mol%
Length (Å)	5403.3±40057	4770.6±11359	4495.7±5582.8
Background (cm^{-1})	-0.082468±0.2894	-0.2659±0.35485	-1.2776±0.51997
Scale	1.767±1.951e8	4.3265-05±2.3935-05	0.00013273±3.9239-05
q-range (nm^{-1})	0.00087 - 0.00324	0.00087 - 0.00324	0.00087 - 0.00324
χ^2	0.08577	0.055845	0.13212

	30 mol%
Length (Å)	3798.3±3140.7
Background (cm^{-1})	-2.2183±0.72688
Scale	0.00021958±5.7682-05
q-range (nm^{-1})	0.00087 - 0.00324
χ^2	0.21834

Details on the mass-balance model and fitting routine

The hypothesis that **(*S*)-PhePyTA** acts as a sequestrator in the polymerization with **(*S*)-BPTA** is studied quantitatively using a mass-balance model. In this mass-balance model, the supramolecular polymerization of **(*S*)-BPTA** is described through the formation of a dimeric nucleus that elongates into supramolecular polymers through sequential monomer additions to the chain end:

$$\mathrm{BPTA} + \mathrm{BPTA} \xrightleftharpoons{K_{\mathrm{n,\,BPTA}}} \mathrm{BPTA}_2$$
$$\mathrm{BPTA}_2 + \mathrm{BPTA} \xrightleftharpoons{K_{\mathrm{n,\,BPTA}}} \mathrm{BPTA}_3$$
$$\mathrm{BPTA}_3 + \mathrm{BPTA} \xrightleftharpoons{K_{\mathrm{e,\,BPTA}}} \mathrm{BPTA}_4$$
$$\vdots$$
$$\mathrm{BPTA}_i + \mathrm{BPTA} \xrightleftharpoons{K_{\mathrm{e,\,BPTA}}} \mathrm{BPTA}_{i+1}$$
$$\vdots$$

with $K_{\mathrm{n,\,BPTA}}$ and $K_{\mathrm{e,\,BPTA}}$ the equilibrium constants of nucleation and elongation of the **(*S*)-BPTA** homopolymerization.

The polymerization of **(*S*)-PhePyTA** is described in a similar way:

$$\mathrm{PhePyTA} + \mathrm{PhePyTA} \xrightleftharpoons{K_{\mathrm{n,\,PhePyTA}}} \mathrm{PhePyTA}_2$$
$$\mathrm{PhePyTA}_2 + \mathrm{PhePyTA} \xrightleftharpoons{K_{\mathrm{n,\,PhePyTA}}} \mathrm{PhePyTA}_3$$
$$\mathrm{PhePyTA}_3 + \mathrm{PhePyTA} \xrightleftharpoons{K_{\mathrm{e,\,PhePyTA}}} \mathrm{PhePyTA}_4$$
$$\vdots$$
$$\mathrm{PhePyTA}_i + \mathrm{PhePyTA} \xrightleftharpoons{K_{\mathrm{e,\,PhePyTA}}} \mathrm{PhePyTA}_{i+1}$$
$$\vdots$$

with $K_{\mathrm{n,\,PhePyTA}}$ and $K_{\mathrm{e,\,PhePyTA}}$ the equilibrium constants of nucleation and elongation of the **(*S*)-PhePyTA** homopolymerization.

The concentration of monomers *i* in the polymerized aggregates is given by:

$$[i]_{\mathrm{polymerized}} = \frac{\sigma_i [i]}{(1 - K_i[i])^2} - \sigma_i [i] \qquad (0.1)$$

with σ_i and [*i*] the cooperativity parameter and concentration of species *i*. The cooperativity parameter σ_i is defined as $\sigma_i = K_{\mathrm{n},i} K_{\mathrm{e},i}^{-1}$ and relates to the enthalpic nucleation penalty *NP* through $\sigma_i = \exp(NP(RT)^{-1})$.

Furthermore, in the model, **(*S*)-BPTA** and **(*S*)-PhePyTA** can aggregate into a heterodimeric complex **BPTA·PhePyTA** in which both monomers are sequestrated and cannot take part in the polymerization:

$$\mathrm{BPTA} + \mathrm{PhePyTA} \xrightleftharpoons{K_{\mathrm{seq}}} \mathrm{BPTA} \cdot \mathrm{PhePyTA}$$

with K_{seq} the equilibrium constant of sequestration into **BPTA·PhePyTA**. The concentration of **(*S*)-BPTA** or **(*S*)-PhePyTA** is given by:

$$[i] = K_{\mathrm{seq}}[\mathrm{BPTA}][\mathrm{PhePyTA}] \qquad (0.2)$$

The total concentration of **(*S*)-BPTA** and **(*S*)-PhePyTA** in the system can then be expressed as:

$$
\begin{aligned}
[\mathbf{BPTA}]_{tot} &= [\mathrm{BPTA}] + [\mathrm{BPTA}]_{polymerized} + [\mathrm{BPTA}]_{seq} \\
&= (1-\sigma_{\mathbf{BPTA}})[\mathbf{BPTA}] + \frac{\sigma_{\mathbf{BPTA}}[\mathbf{BPTA}]}{(1-K_{\mathbf{BPTA}}[\mathbf{BPTA}])^2} + K_{seq}[\mathbf{BPTA}][\mathbf{PhePyTA}]
\end{aligned} \tag{0.3}
$$

And

$$
\begin{aligned}
[\mathbf{PhePyTA}]_{tot} &= [\mathbf{PhePyTA}] + [\mathbf{PhePyTA}]_{polymerized} + [\mathbf{PhePyTA}]_{seq} \\
&= (1-\sigma_{\mathbf{PhePyTA}})[\mathbf{PhePyTA}] + \frac{\sigma_{\mathbf{PhePyTA}}[\mathbf{PhePyTA}]}{(1-K_{\mathbf{PhePyTA}}[\mathbf{PhePyTA}])^2} + K_{seq}[\mathbf{BPTA}][\mathbf{PhePyTA}]
\end{aligned} \tag{0.4}
$$

for **(*S*)-BPTA** and **(*S*)-PhePyTA**, respectively. Further details on the general derivation of the mass-balance equations can be found elsewhere.[11] The above described system of two equations is solved in an iterative manner using a nested binary search algorithm.[12]

To describe the temperature-dependent behavior of the aggregation pathways, the equilibrium constants of aggregation processes *i* are dependent on the temperature through the Van 't Hoff equation:

$$
K_i = \exp\left(\frac{-\Delta H_i}{RT} - \frac{\Delta S_i}{R}\right) \tag{0.5}
$$

The fitting of the model to the data is done in Matlab 2019a using the Levenberg-Marquardt algorithm and the lsqnonlin function. The thermodynamic values of the homopolymerization of **(*S*)-BPTA** is obtained from previous work[13,14] and the values of the homopolymerization of **(*S*)-PhePyTA** are obtained from the Van 't Hoff. The nucleation penalty of **(*S*)-PhePyTA** through the Van 't Hoff plot and is therefore assumed equal to the nucleation penalty of **(*S*)-BPTA**. Since at the concentrations at which the sequestration is measured hardly any **(*S*)-PhePyTA** polymers are present, this assumption does not have a significant impact on our calculations. The molar ellipticities of the homopolymers of **(*S*)-BPTA** and **(*S*)-PhePyTA** are obtained from the CD intensity at 26.7 °C in the cooling curves of the homopolymerizations and determined at $2.11 \cdot 10^6$ and $1.33 \cdot 10^5$ L mol^{-1} cm^{-1}, respectively. The CD intensity of **BPTA·PhePyTA** is set at 0. The enthalpy and entropy of the sequestration equilibrium, K_{seq}, are used as fitting parameters. Using Latin hypercube sampling, 200 random starting sets consisting of an enthalpy value between −70 and −100 kJ/mol and an entropy value between −100 and −200 J/mol/K are obtained and subsequently optimized. In the fitting routine, the composition of the system is calculated using mass-balance equations (1.3) and (1.4) and translated into a calculated CD trace. In the optimization, a cost vector containing the difference between the experimental and calculated CD intensity for every experimentally probed temperature is calculated. Optimization of each of the 200 initial parameter sets is done by minimizing the sum of the squared components of the cost vector, after which an optimized dataset is obtained. The optimized dataset with the lowest norm of the residual cost vector is selected as the best fit.

NMR spectra

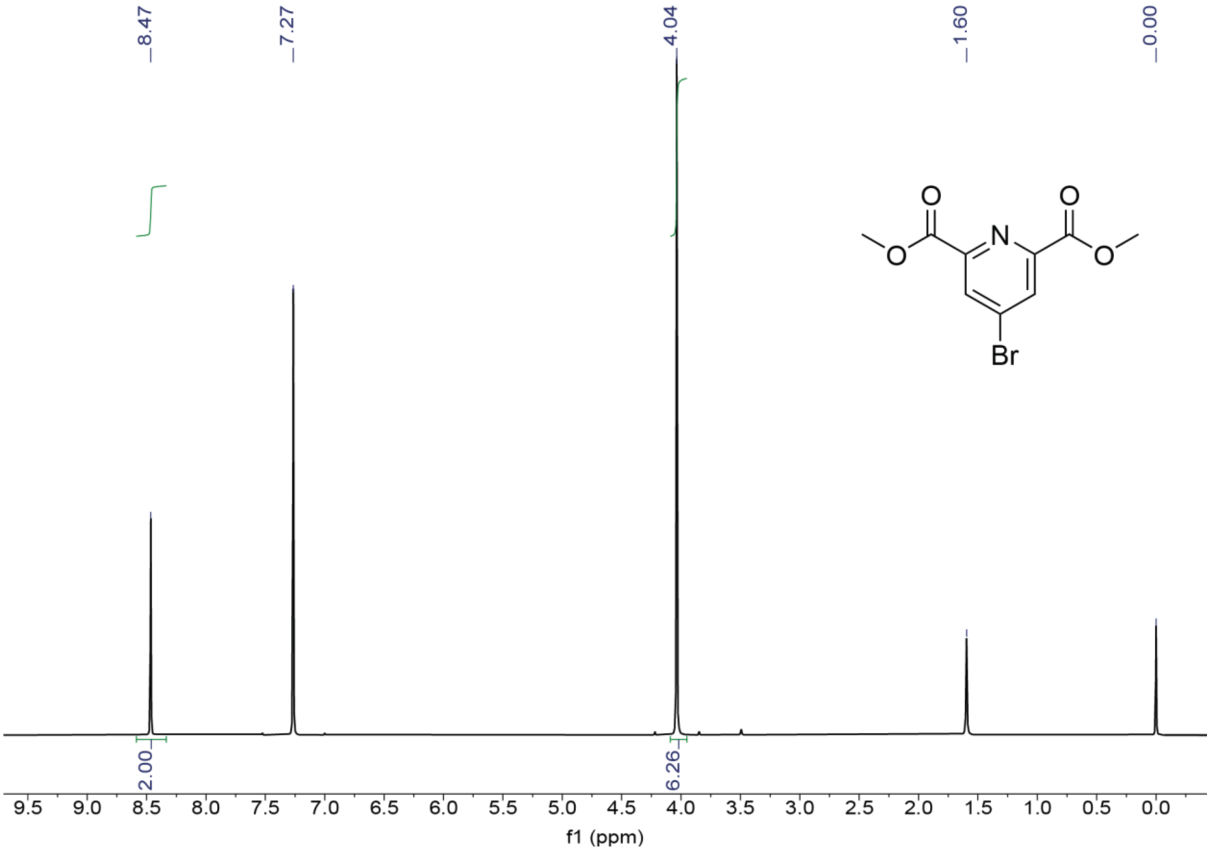

Figure S28 ^{1}H NMR spectrum of **1.**

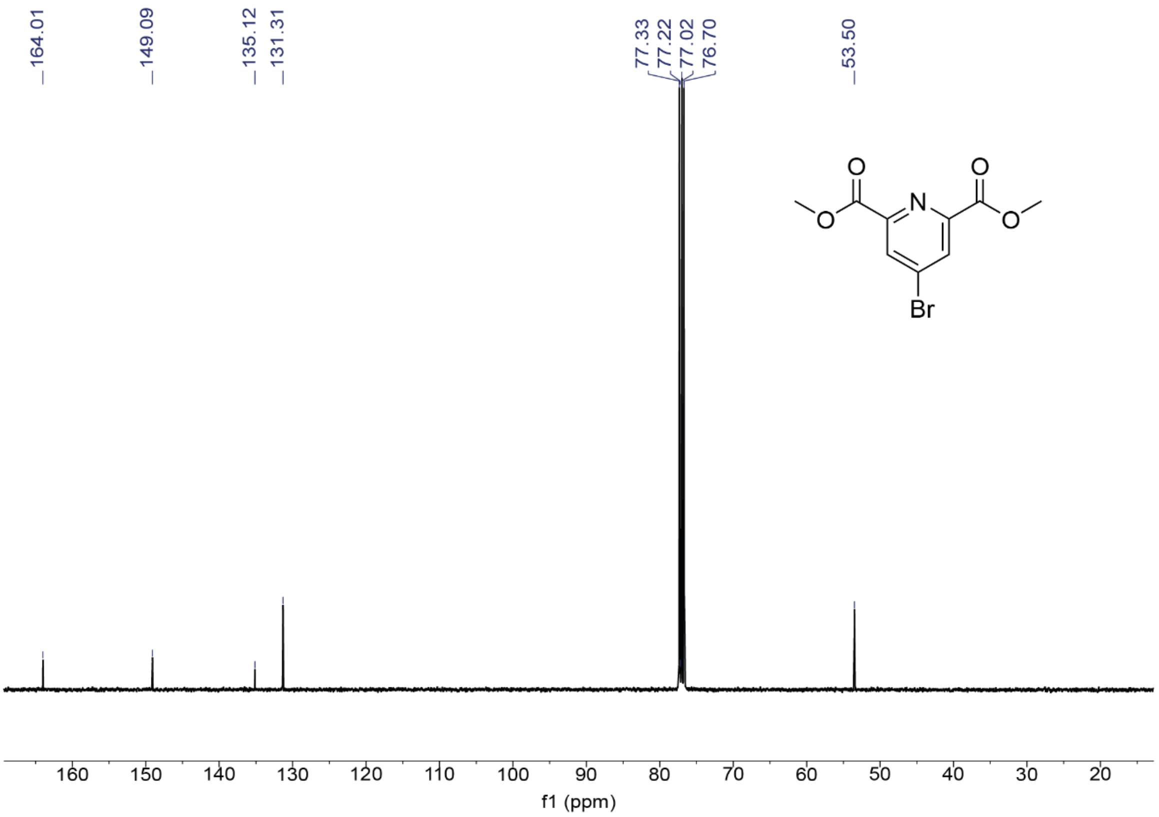

Figure S29 ^{13}C NMR spectrum of **1**.

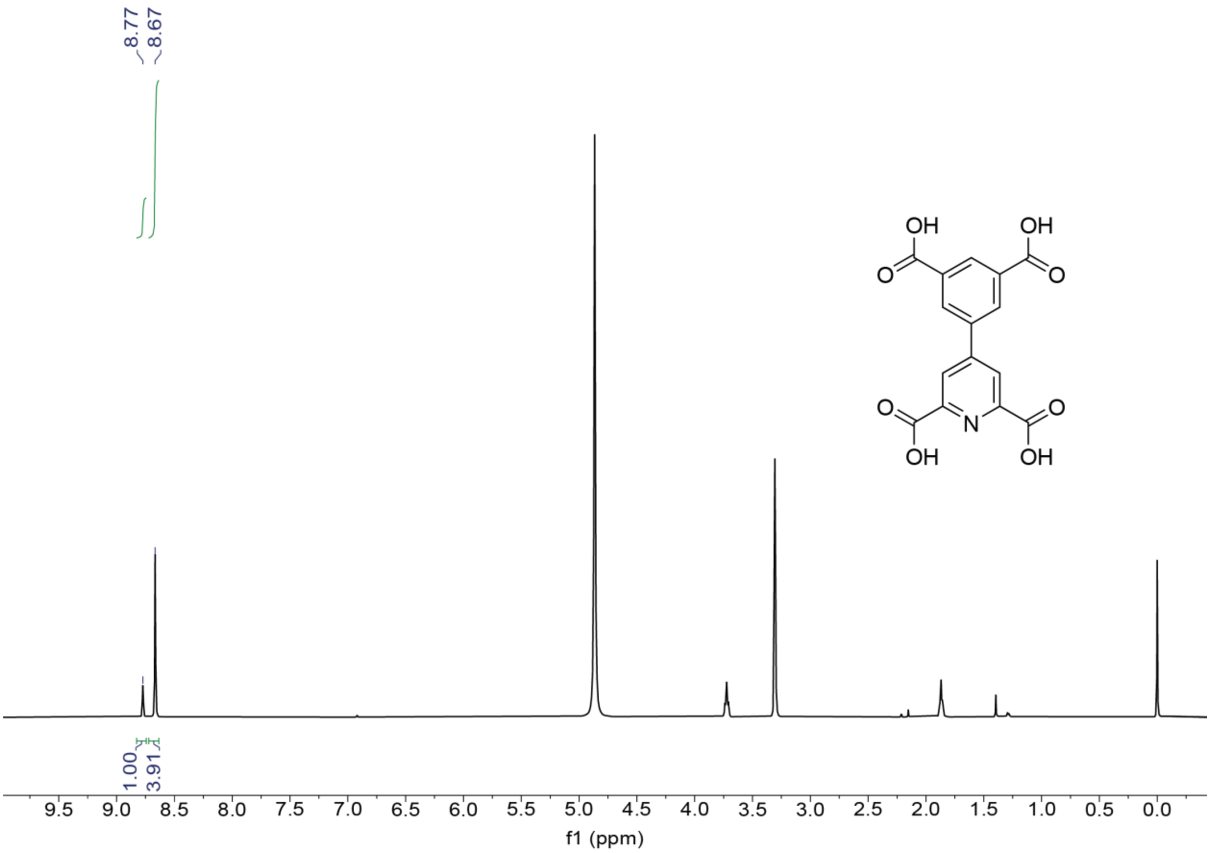

Figure S30 ^{1}H NMR spectrum of **2**.

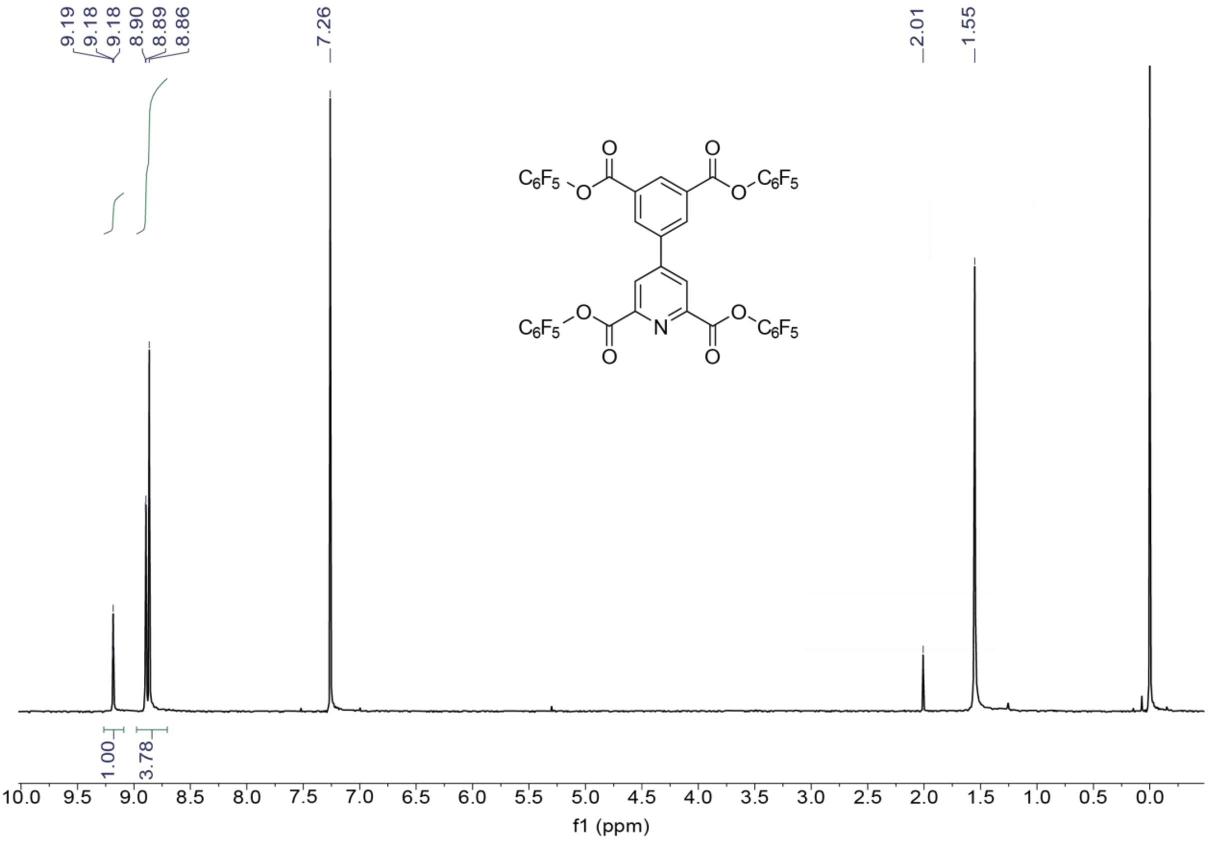

Figure S31 ^{1}H NMR spectrum of **3**.

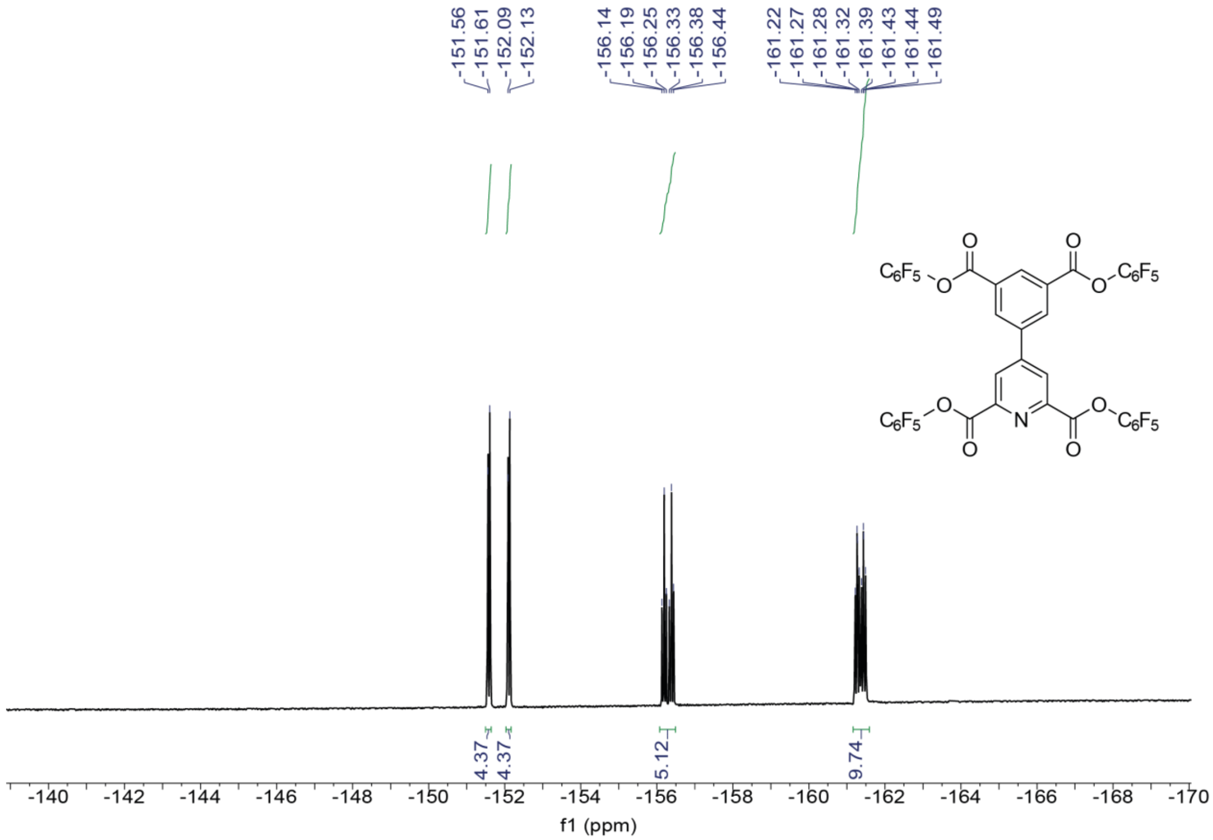

Figure S32 ^{19}F NMR spectrum of **3**.

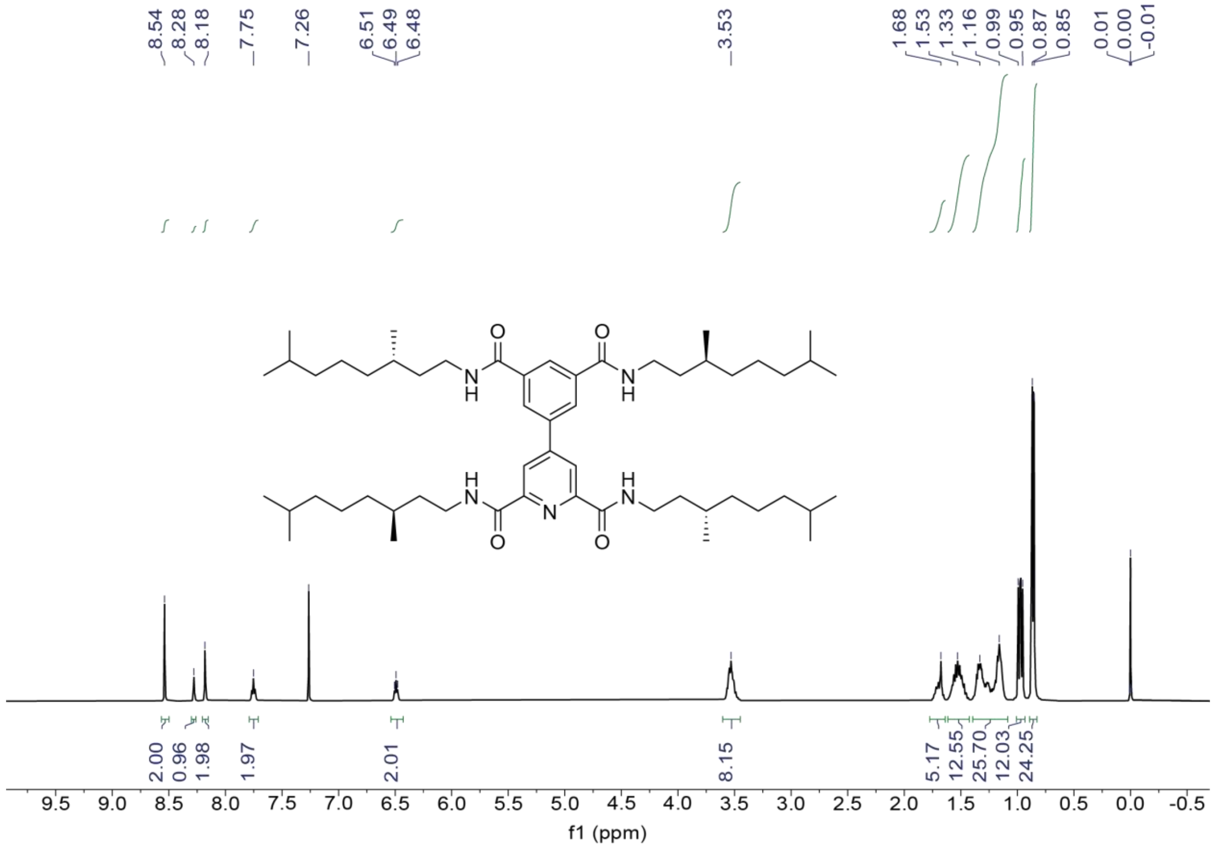

Figure S33 ^{1}H NMR spectrum of **(*S*)-PhePyTA**.

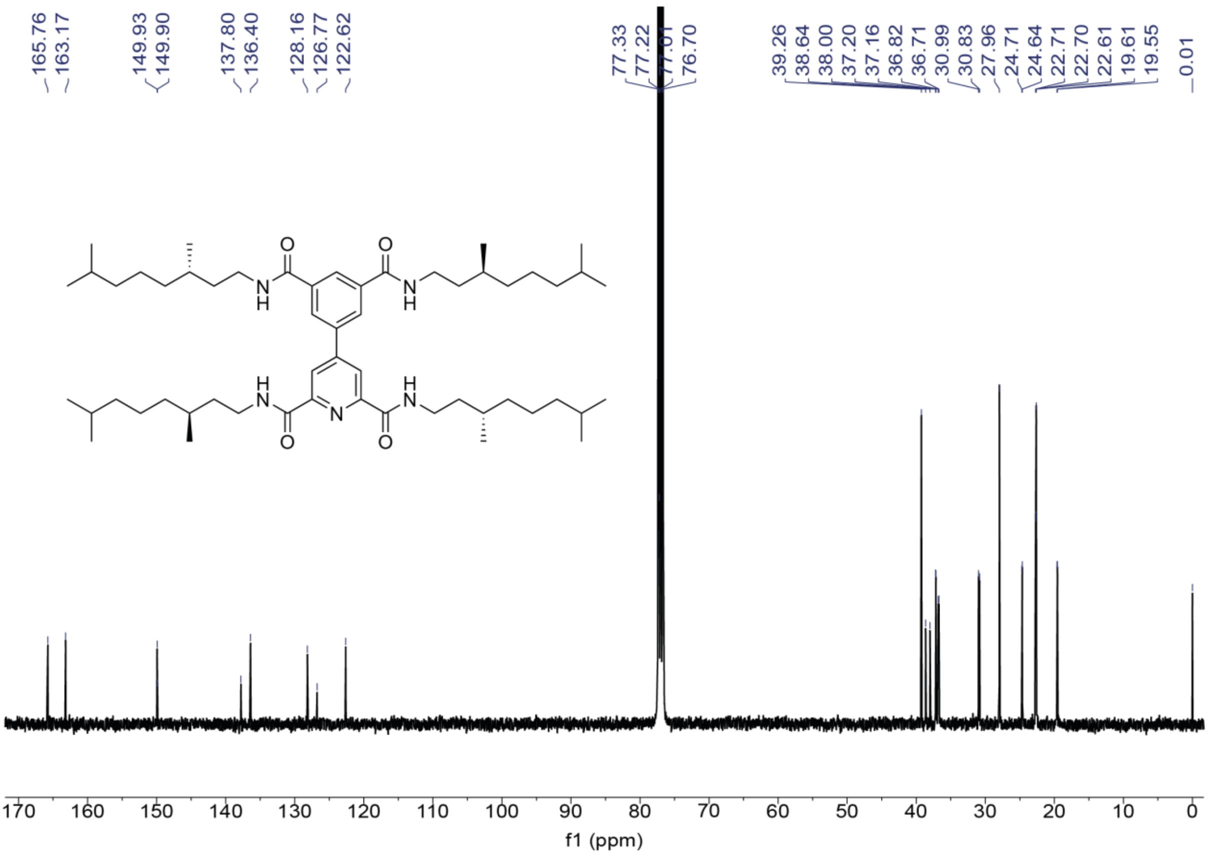

Figure S34 ^{13}C NMR spectrum of **(*S*)-PhePyTA**.

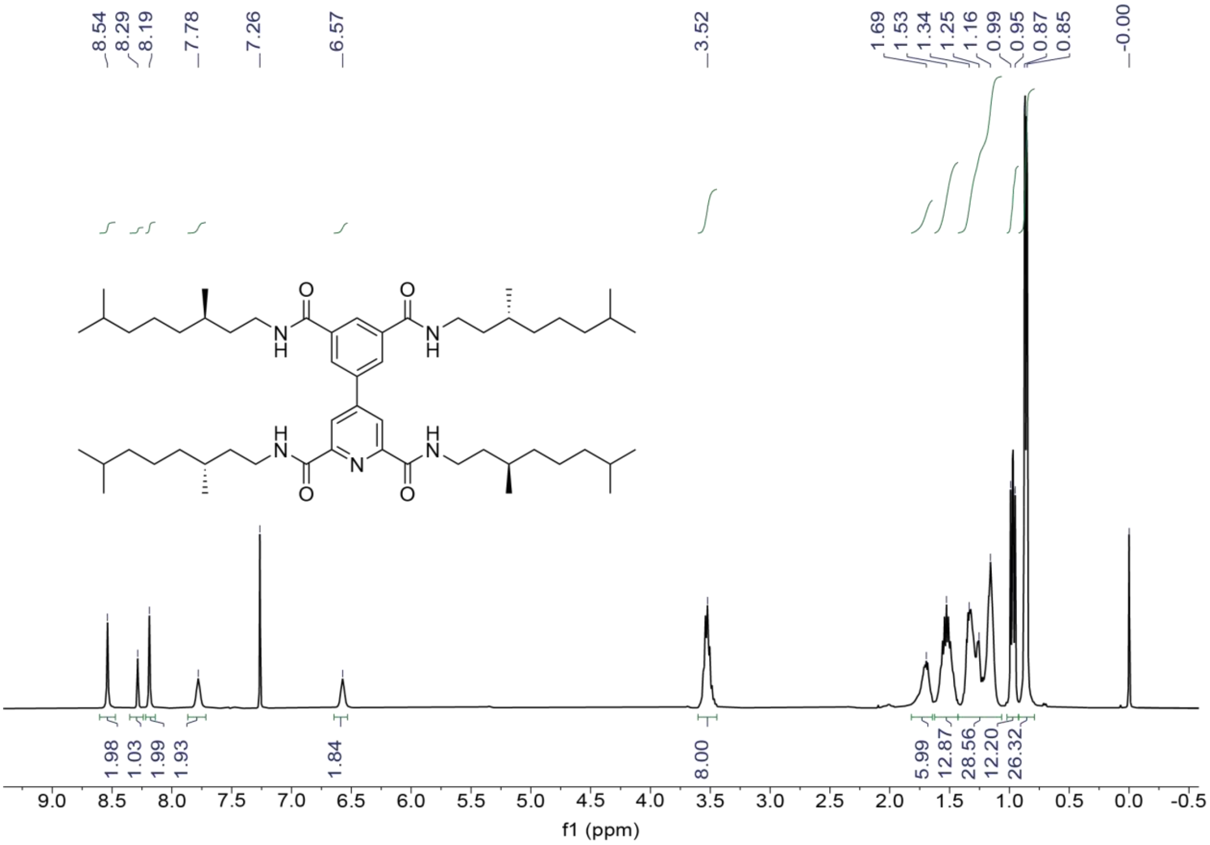

Figure S35 ^{1}H NMR spectrum of **(*R*)-PhePyTA**.

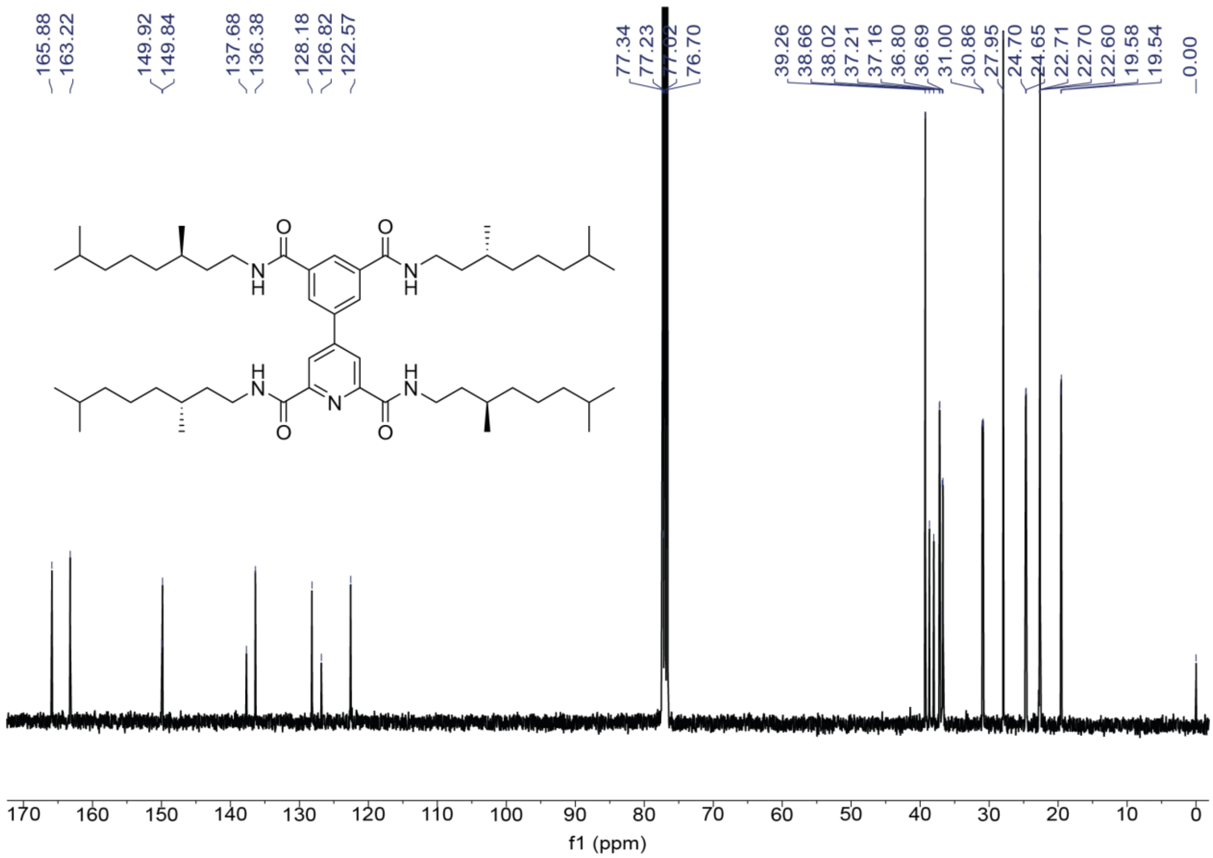

Figure S36 ^{13}C NMR spectrum of **(*R*)-PhePyTA**.

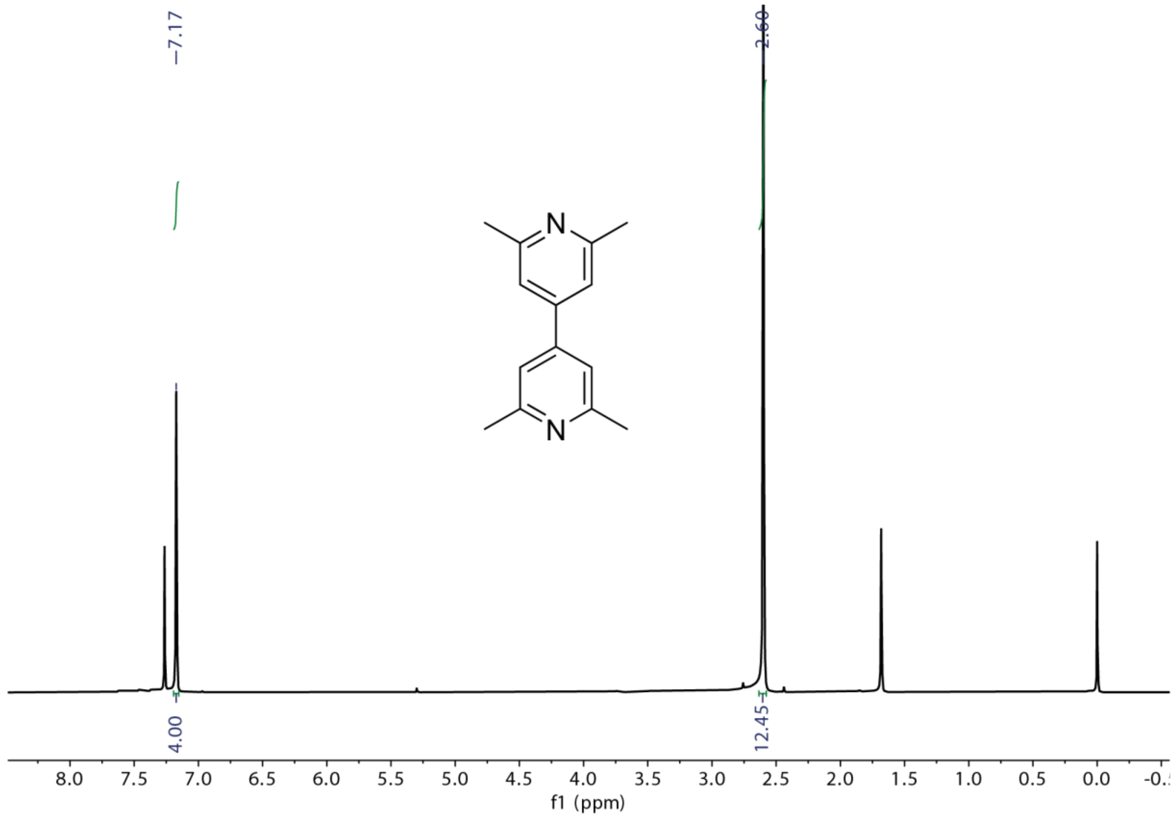

Figure S37 ^{1}H NMR spectrum of **4**.

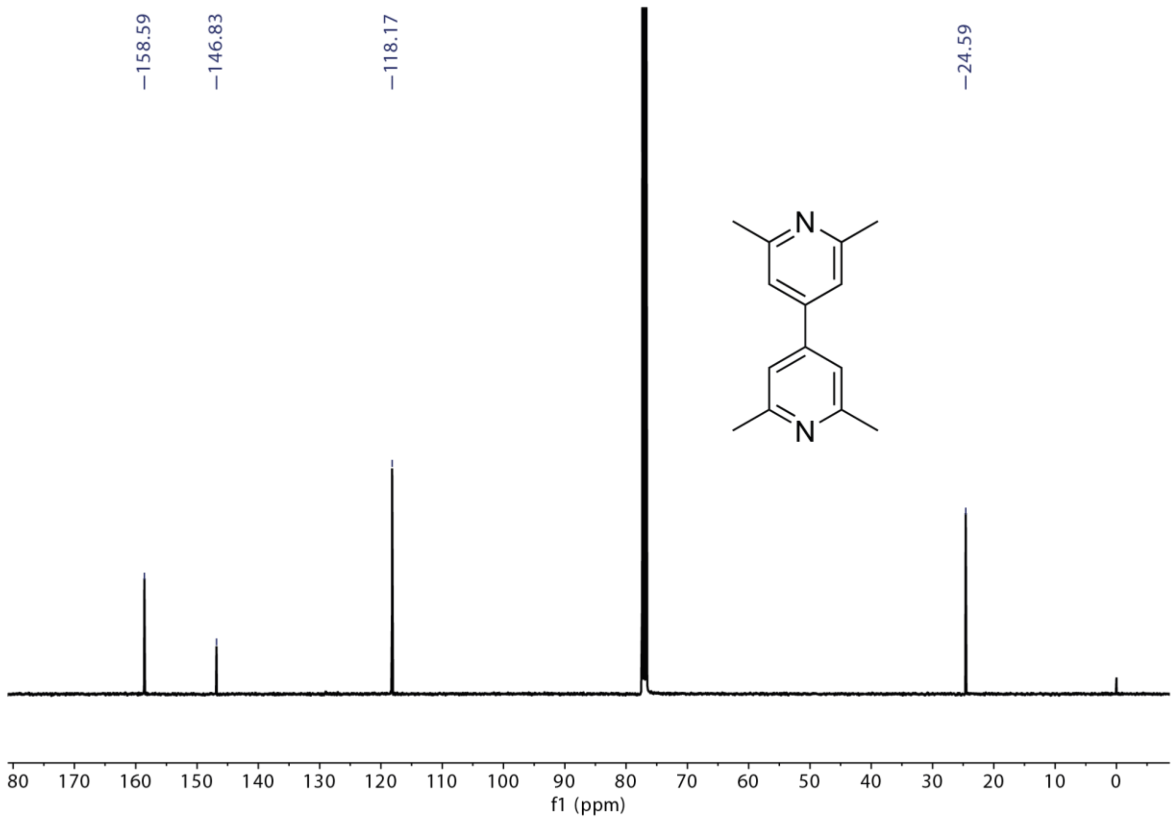

Figure S38 ^{13}C NMR spectrum of **4**.

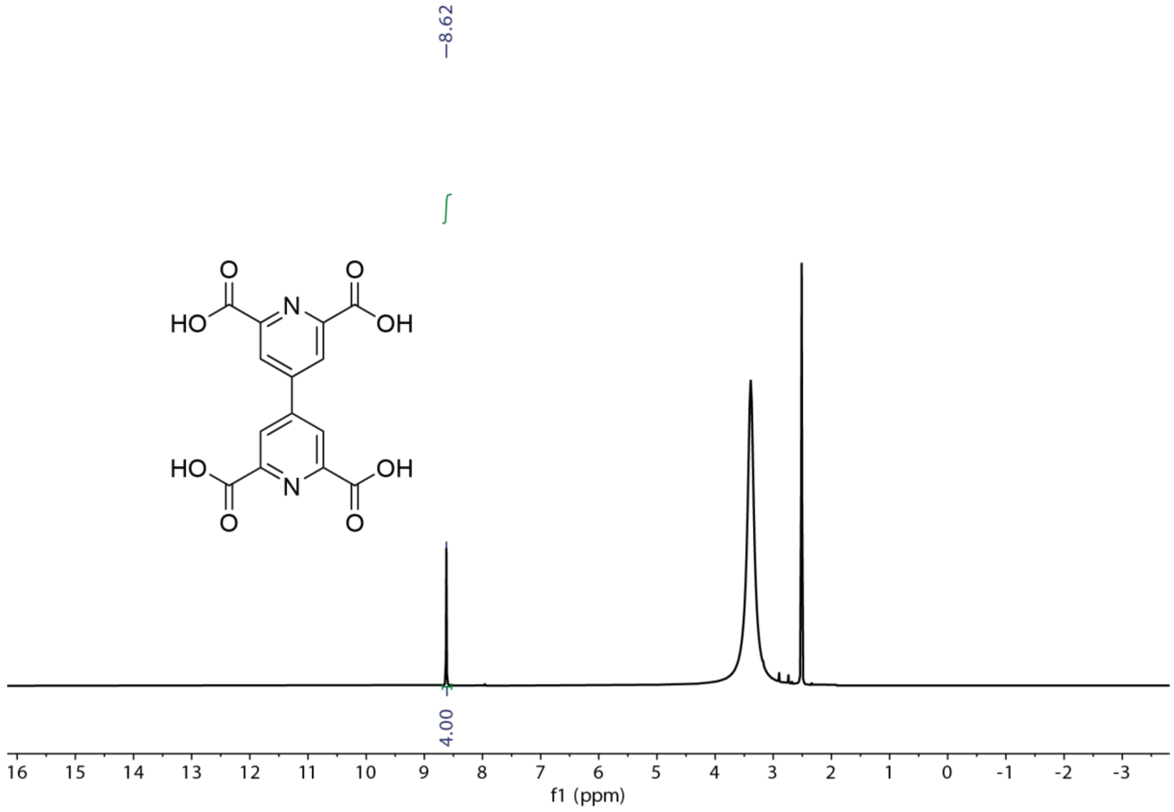

Figure S39 ^{1}H NMR spectrum of **5**.

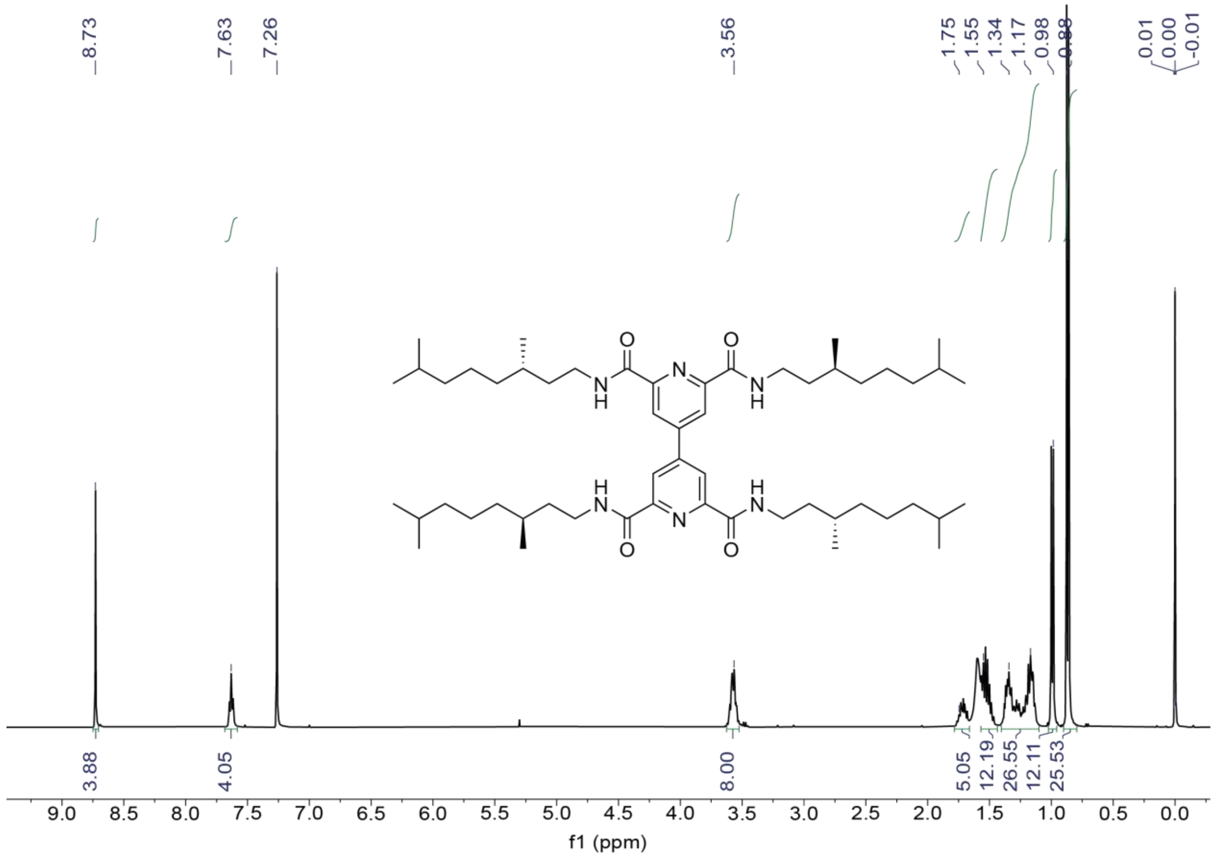

Figure S40 ^{1}H NMR spectrum of **(*S*)-BiPyTA**.

MALDI-ToF spectra

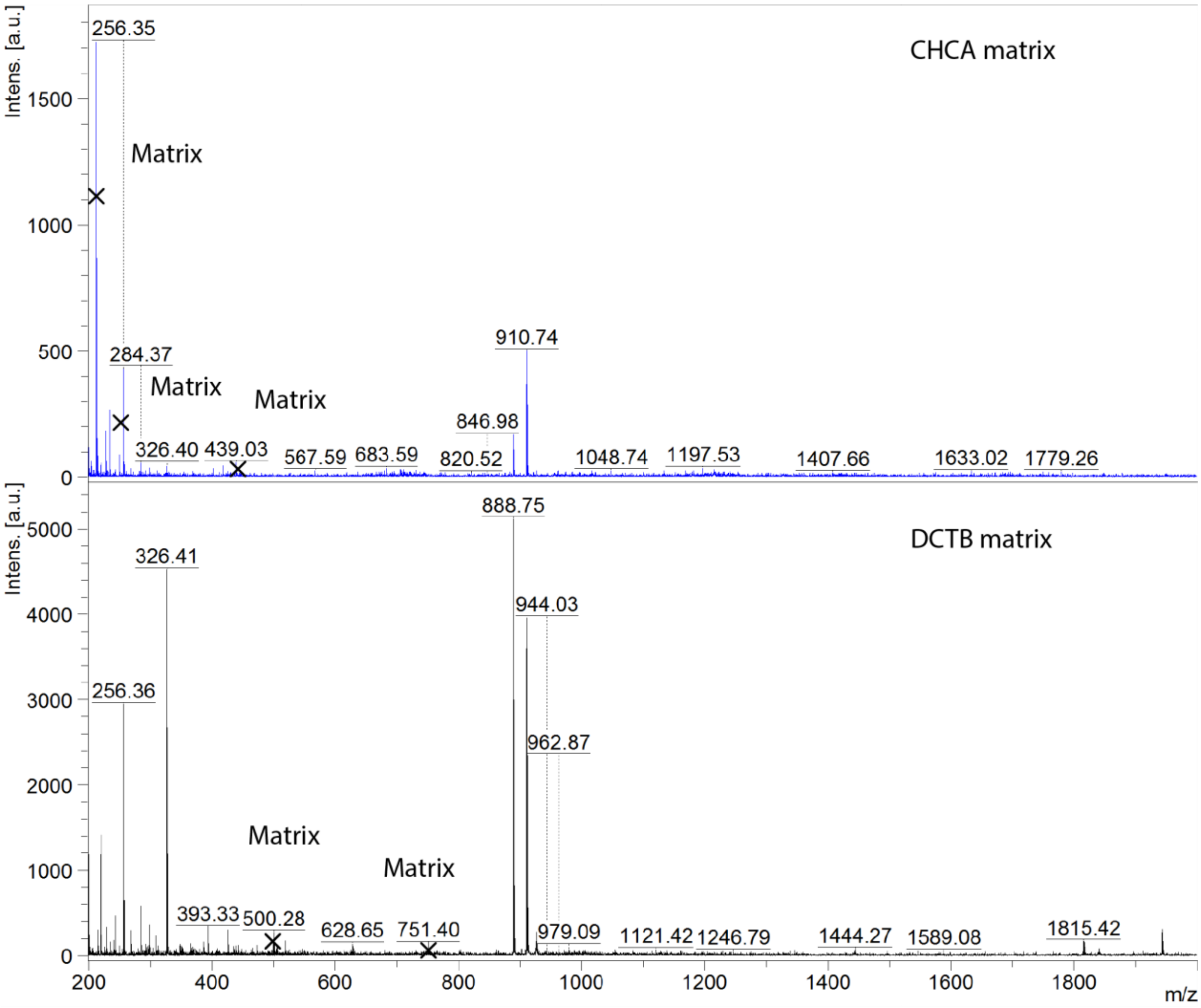

Figure S41 MALDI-ToF spectra with CHCHA and DCTB matrices of ***(S)*-PhePyTA**.

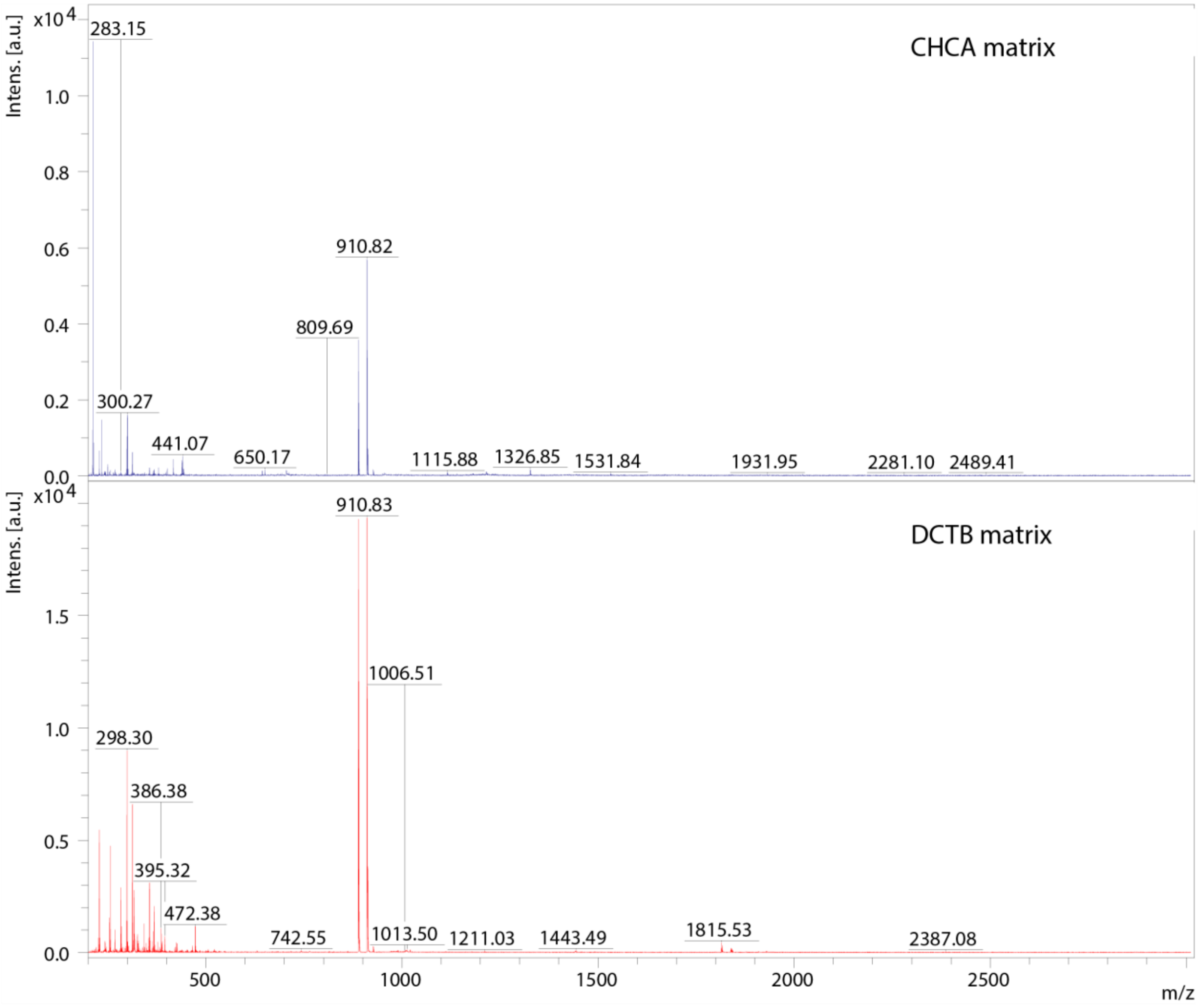

Figure S42 MALDI-ToF spectra with CHCHA and DCTB matrices of **(*R*)-PhePyTA**.

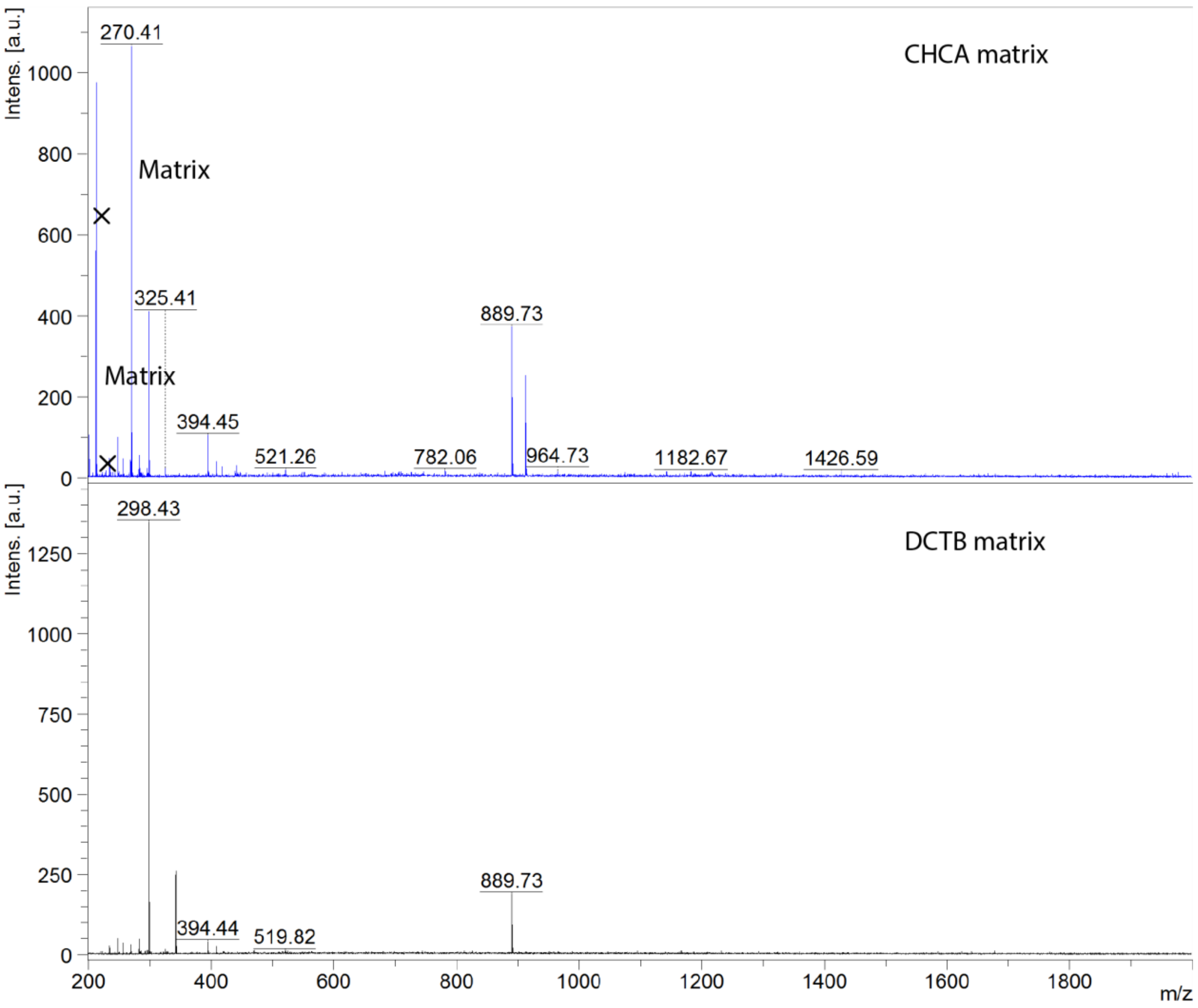

Figure S43 MALDI-ToF spectra with CHCHA and DCTB matrices of **(*S*)-BiPyTA**.

Supplementary references

(1) Ślęczkowski, M. L.; Meijer, E. W.; Palmans, A. R. A. Cooperative Folding of Linear Poly(Dimethyl Siloxane)s via Supramolecular Interactions. *Macromol. Rapid Commun.* **2017**, *38* (24), 1700566.

(2) Stals, P. J. M.; Smulders, M. M. J.; Martín-Rapún, R.; Palmans, A. R. A.; Meijer, E. W. Asymmetrically Substituted Benzene-1,3,5-tricarboxamides: Self-Assembly and Odd–Even Effects in the Solid State and in Dilute Solution, *Chem. Eur. J.* **2009**, *15* (9), 2071 – 2080

(3) SasView 5.0.3 http://www.sasview.org/.

(4) te Velde, G.; Bickelhaupt, F. M.; Baerends, E. J.; Fonseca Guerra, C.; van Gisbergen, S. J. A.; Snijders, J. G.; Ziegler, T. Chemistry with ADF. *J. Comput. Chem.* **2001**, *22* (9), 931–967.

(5) Swart, M.; Bickelhaupt, F. M. QUILD: QUantum-Regions Interconnected by Local Descriptions. *J. Comput. Chem.* **2008**, *29* (5), 724–734.

(6) Nicu, V. P.; Neugebauer, J.; Wolff, S. K.; Baerends, E. J. A Vibrational Circular Dichroism Implementation within a Slater-Type-Orbital Based Density Functional Framework and Its Application to Hexa- and Hepta-Helicenes. *Theor. Chem. Acc.* **2008**, *119* (1–3), 245–263.

(7) Shen, J.; Zhu, C.; Reiling, S.; Vaz, R. A Novel Computational Method for Comparing Vibrational

Circular Dichroism Spectra. *Spectrochim. Acta Part A Mol. Biomol. Spectrosc.* **2010**, *76* (3–4), 418–422.

(8) Yoshizawa, M.; Nakagawa, J.; Kumazawa, K.; Nagao, M.; Kawano, M.; Ozeki, T.; Fujita, M. Discrete Stacking of Large Aromatic Molecules within Organic-Pillared Coordination Cages. *Angew. Chem. Int. Ed.* **2005**, *44* (12), 1810–1813.

(9) Soto, E.; MacDonald, J. C.; Cooper, C. G. F.; McGimpsey, W. G. A Non-Covalent Strategy for the Assembly of Supramolecular Photocurrent-Generating Systems. *J. Am. Chem. Soc.* **2003**, *125* (10), 2838–2839.

(10) Suzuki, A.; Aratsu, K.; Datta, S.; Shimizu, N.; Takagi, H.; Haruki, R.; Adachi, S.; Hollamby, M.; Silly, F.; Yagai, S. Topological Impact on the Kinetic Stability of Supramolecular Polymers. *J. Am. Chem. Soc.* **2019**, *141* (33), 13196–13202.

(11) Zhao, D.; Moore, J. S. Nucleation-Elongation: A Mechanism for Cooperative Supramolecular Polymerization. *Org. Biomol. Chem.* **2003**, *1* (20), 3471–3491.

(12) Rao, K. V.; Mabesoone, M. F. J.; Miyajima, D.; Nihonyanagi, A.; Meijer, E. W.; Aida, T. Distinct Pathways in “Thermally Bisignate Supramolecular Polymerization”: Spectroscopic and Computational Studies. *J. Am. Chem. Soc.* **2020**, *142* (1), 598–605.

(13) Van Zee, N. J.; Adelizzi, B.; Mabesoone, M. F. J.; Meng, X.; Aloi, A.; Zha, R. H.; Lutz, M.; Filot, I. A. W.; Palmans, A. R. A.; Meijer, E. W. Potential Enthalpic Energy of Water in Oils Exploited to Control Supramolecular Structure. *Nature* **2018**, *558* (7708), 100–103.

(14) Van Zee, N. J.; Mabesoone, M. F. J.; Adelizzi, B.; Palmans, A. R. A.; Meijer, E. W. Biasing the Screw-Sense of Supramolecular Coassemblies Featuring Multiple Helical States. *J. Am. Chem. Soc.* **2020**, *142* (47), 20191–20200.
